# Prognosis of neonates receiving invasive mechanical ventilation in low-resource settings: a systematic review and prognostic meta-analysis

**DOI:** 10.1007/s00431-026-07016-z

**Published:** 2026-05-07

**Authors:** Vijay Kumar Krishnegowda, Viraraghavan Vadakkencherry Ramaswamy, Prathik Bandiya, Tapas Bandyopadhyay, Thangaraj Abiramalatha, Abdul Kareem Pullattayil, Daniele Trevisanuto

**Affiliations:** 1 Department of Neonatology, Institute of Medical Sciences and SUM Hospital, Bhubaneswar, Odisha India; 2 Department of Neonatology, Fernandez Foundation, Hyderabad, Telangana India; 3 Department of Neonatology, Ankura Hospital for Women and Children, Hyderabad, Telangana India; 4 Department of Neonatology, Indira Gandhi Institute of Child Health, Bengaluru, Karnataka India; 5 Vardhman Mahavir Medical College and Safdarjung Hospital, New Delhi, India; 6 Department of Neonatology, Kovai Medical Center and Hospital (KMCH), Coimbatore, Tamil Nadu India; 7 Health Sciences Librarian Queen’s University, Kingston, Canada; 8 Department of Woman’s and Child’s Health, University of Padova, Padua, Italy

**Keywords:** Preterm, Neonates, Mechanical ventilation, Mortality, Respiratory distress, Low- and middle-income countries

## Abstract

Provision of invasive mechanical ventilation (IMV) in the neonatal intensive care has seen a steady rise in low-esource settings (LRS). However, outcomes among those exposed to IMV remain under-reported, with the current evidence base being restricted to single-centre observational studies, thus limiting comparative analyses and effective healthcare planning. This study aims to estimate the pooled proportion of mortality and morbidity among neonates exposed to IMV in low-resource settings. Medline, Embase, and CENTRAL were searched until 22 August 2025. Randomised and non-randomised studies were included. Two reviewers, blinded to each other, extracted data independently. Proportion-based meta-analyses using random-effects model were performed. Risk of bias was assessed using ROBINS-E, and evidence-certainty was evaluated using the GRADE approach. One hundred of 117 studies were included, with most conducted in South Asia. In-hospital mortality was reported in 68 studies (7193 neonates), with a pooled estimate of 45% (39%–50%), evidence-certainty being very low. Among the secondary outcomes, the pooled rates were as follows: bronchopulmonary dysplasia, 10% (5%–18%); intraventricular haemorrhage (any grade), 10% (5%–19%); necrotising enterocolitis (any stage), 14% (6%–31%); retinopathy of prematurity (any stage), 33% (22%–46%); ventilator-associated pneumonia, 21% (14%–29%); sepsis, 32% (25%–40%) and pulmonary haemorrhage, 9% (6%–14%). Evidence-certainty for all the secondary outcomes was also very low. Subgroup analysis comparing two distinct time epochs revealed a significant difference in mortality, 43% (36%–50%) (*I*^2^ = 93.5%) in the post-2010 epoch compared to 55% (48%–63%) (*I*^2^ = 82.9%) in the pre-2010 epoch (*p* = 0.004). Heterogeneous outcome definitions and predominance of unadjusted analyses across studies limit the existing evidence.

*Conclusions*: In LRS, the mortality and morbidity rates among neonates receiving IMV remain substantially high with a modest improvement in survival in the past decade. Improving outcomes mandates moving beyond access to the provision of IMV to investing in comprehensive training and scaling up critical auxiliary resources. Future research must adopt standardised outcome definitions and adjusted analyses to precisely quantify the impact of IMV in LRS.

**Supplementary Information:**

The online version contains supplementary material available at 10.1007/s00431-026-07016-z.

## Introduction

Neonatal mortality accounts for a substantial share of global under-5 deaths. In 2023, an estimated 2.3 million newborns died, with the majority of these deaths occurring in low-resource settings (LRS) across South Asia and sub-Saharan Africa. The global neonatal mortality rate (NMR) has decreased markedly over the past decades, from 37 per 1000 live births in 1990 to 17 per 1000 live births in 2023 [1]. This observed trend, though promising, is not equitably distributed across all settings. Furthermore, recent data indicate that the reduction in neonatal mortality has slowed in the past decade. Consequently, a fourfold acceleration in the implementation of proven maternal and neonatal interventions is required to achieve the Sustainable Development Goal (SDG) target of fewer than 12 neonatal deaths per 1000 live births by 2030 [2, 3].

To meet the SDG NMR target by 2030, international organisations such as the United Nations Children’s Fund, the World Health Organization, and the United Nations Population Fund emphasise strengthening care across the continuum of maternal and newborn health, with a particular focus on providing timely and optimal care for small and sick neonates [4, 5]. However, expansion of facility-based neonatal intensive care services and its translation into improved neonatal outcomes in resource constrained settings has not been adequately studied.

Prematurity, perinatal asphyxia, and neonatal sepsis remain the leading causes of neonatal mortality in LRS [6]. These conditions often require the provision of respiratory support, including invasive mechanical ventilation (IMV). However, the implementation of IMV requires considerable resources, including healthcare providers with expertise. Though access to IMV across LRS has bettered over the past decade [7], outcomes of neonates who receive IMV remain highly variable and are influenced by multiple factors. Despite the widespread use of IMV in LRS in recent years, data on mortality and various morbidities among neonates who were ventilated remain fragmented and are largely derived from single-center observational studies evaluating neonates with widely varying sickness profiles. The absence of a comprehensive analysis of available data limits benchmarking, comparison, and planning for neonatal intensive care services across all tiers of the health system, from facility level to global policy.

Therefore, we conducted this systematic review and meta-analysis to quantify in-hospital mortality and critical morbidities among neonates receiving IMV in LRS. The objective of this systematic review was to quantify the burden of the aforementioned outcomes, thereby informing and facilitating stakeholder efforts to improve neonatal intensive care in LRS.

## Methods

This systematic review was conducted and reported in accordance with the PRISMA 2020 guidelines [8], and the protocol was registered in PROSPERO CRD420251143610 [9].

### Eligibility criteria

Randomised and non-randomised studies of different designs were eligible for inclusion if they reported outcomes among ventilated neonates from low-income and lower-middle-income countries, as categorised by the World Bank 2025–2026 per-capita income classifications [10]. Both preterm and term neonates needing ventilation for a varied aetiology were included. We excluded studies which included neonates who received ventilation for a brief period of time, such as after surfactant administration, (Intubate-surfactant-extubate (INSURE)). Studies that enrolled neonates requiring surgical interventions and received IMV were excluded. We also excluded studies that met all inclusion criteria but did not report extractable outcome data.

### Literature search

A comprehensive literature search of the electronic databases, namely, Medline, Embase, and the Cochrane Central Register of Controlled Trials (CENTRAL) was conducted from inception until 22 August 2025. No language restrictions were applied. Published full-text articles and conference abstracts were eligible for inclusion. Three reviewers (VK, PB, and TB), blinded to each other, screened titles and abstracts and assessed full texts for eligibility in duplicates using the Covidence platform (Veritas Health Innovation, Melbourne), with disagreements resolved by consensus [11]. The reference lists of included studies and relevant review articles were screened for additional eligible studies. The search strategy is provided in Supplemental file 2, Appendix 1.

### Data collection process and data items

Data were extracted from eligible studies independently by two reviewers (VK and PB) using a predefined data sheet. Disagreements were resolved by a third reviewer (VVR). Study authors were not contacted for additional information. Non-English studies were translated using Google Translate (Google, California). The primary outcome was in-hospital mortality. Secondary outcomes included bronchopulmonary dysplasia (BPD) (as defined by study authors), intraventricular haemorrhage (IVH) (any grade and grade ≥ 2), periventricular leukomalacia (PVL) (as defined by authors), necrotising enterocolitis (NEC) (any stage and stage ≥ 2), retinopathy of prematurity (ROP) (of any severity and that requiring intervention), sepsis (as defined by authors), ventilator-associated pneumonia (VAP) (as defined by authors), and pulmonary haemorrhage.

### Risk of bias assessment

Risk of bias (ROB) was assessed using the Risk of Bias in Non-randomised Studies–of Exposure (ROBINS-E) tool, considering IMV as an exposure or clinical need rather than an intervention. Key potential confounders were prespecified and considered during the assessment.

### Data syntheses

Proportional meta-analyses were performed using the *Metaprop* function from the *meta* package in R programme (version 4.5.1) (R Foundation for Statistical Computing, Vienna) [12]. To stabilise variances and to appropriately handle proportions close to 0 or 1, proportions were logit transformed. Pooled estimates with their 95% confidence intervals (CI) were calculated using random-effects models by the inverse variance method and subsequently back-transformed to proportions. Random-effects models were chosen as we anticipated heterogeneity across studies. Between-studies heterogeneity parameter tau-squared was quantified using the restricted maximum likelihood (REML) estimator. Heterogeneity was also assessed using the *I*^2^ statistic. For outcomes reported as odds ratios (OR), meta-analysis was performed, provided there were three or more studies using a random-effects model with generic inverse variance, and the DL estimator to quantify tau-squared. Publication bias was assessed using funnel plots and Begg’s rank test [13].

### Subgroup analysis

A priori subgroup analyses were performed based on gestational age category of the included neonates (< 34 weeks, < 37 weeks and < 40 weeks), by country, geographical region, and underlying respiratory disease etiology (respiratory distress syndrome, meconium aspiration syndrome, and pulmonary hypertension).

### Sensitivity analysis

Prespecified sensitivity analyses were performed by excluding studies with fewer than 50 participants, by excluding studies with a high overall ROB and by comparing survival between two epochs (recruitment before 2010 and after 2010).

### Certainty of evidence assessment

Grading of Recommendations Assessment, Development, and Evaluation (GRADE) approach was utilised to assess the certainty of evidence (CoE) [14]. As ROB was assessed using ROBINS-E, with confounding and selection bias being two amongst the other domains, the CoE started as ‘high’ and was further downrated if needed for ROB, inconsistency, indirectness, imprecision, and publication bias [14]. CoE was uprated by one level in scenarios of plausible confounding, large effects or dose–response relationship, provided the CoE has not been downrated for risk of bias or imprecision. CoE was assessed separately for each type of analysis, including pooled proportional outcomes and pooled odds ratios. Narrative evidence was graded separately only where it could meaningfully change the CoE beyond the quantitative findings.

## Results

**Fig. 1:**
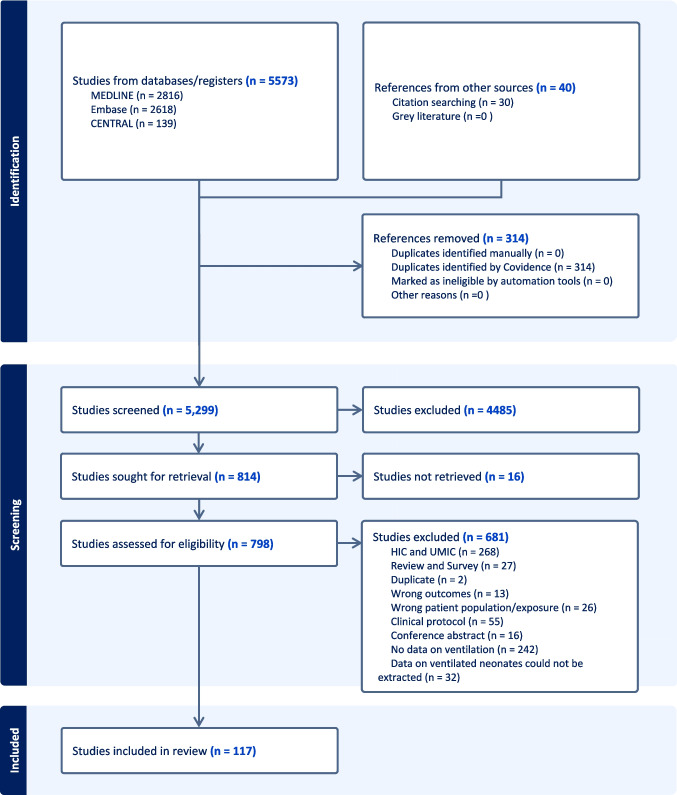
PRISMA diagram of literature search

A total of 5259 studies were screened for inclusion, of which 784 were selected for full-text screening. One hundred and seventeen studies [15–131] were included in the meta-analysis (Fig. 1). The studies included in the analysis utilised various designs, including prospective cohort, retrospective, cross-sectional, and case–control methodologies, with a predominance of prospective cohort studies. Majority of studies were conducted in LMICs, with only three studies from LICs [15–17]. Country-wise, 63 studies were from India [19, 21, 24, 26, 28, 30, 33, 35, 36, 41, 43, 45–48, 50–52, 54, 56, 61, 63–66, 68, 70–72, 74–77, 79–81, 83, 84, 86–88, 90, 92, 93, 101, 102, 104, 105, 107, 108, 111, 112, 114, 116–119, 123, 125, 126, 129–131], 11 from Pakistan [18, 20, 39, 57, 58, 69, 78, 91, 97, 98, 119], 6 from Nepal,[25, 31, 32, 62, 67, 82], 7 from Bangladesh [27, 34, 37, 42, 49, 53, 132], 13 from Egypt [2, 29, 40, 44, 55, 59, 85, 100, 103, 110, 115, 120, 133], 3 each from Ethiopia [15–17], Kenya [60, 95, 99], and Tunisia [89, 127, 128], 2 each from Palestine [109, 121] and Tanzania [23, 96], and 1 each from Jordan [73], Morocco [122], Syria [94], and Vietnam [106]. The study populations were diverse, comprising both preterm and term neonates who required IMV and were diagnosed with various disease pathologies. These included confirmed or suspected sepsis [23, 30, 87, 88, 119, 121, 123, 124, 127–130], respiratory distress syndrome [44, 60, 64, 66, 77, 132], and meconium aspiration syndrome [20], among others. The characteristics of the included studies are summarised in Supplemental file 2, Table S1.

### Risk of bias

**Fig. 2:**
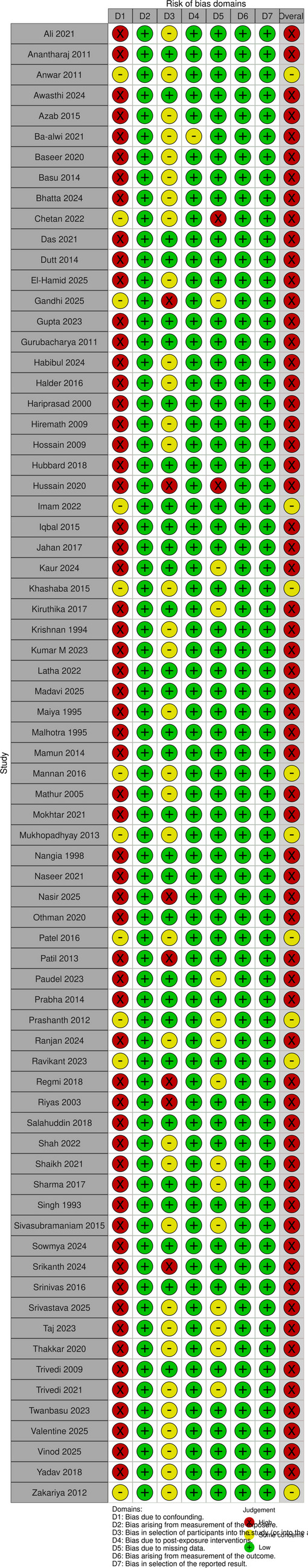
ROBINS-E risk of bias assessment of individual studies for outcome in-hospital mortality among neonates receiving mechanical ventilation

ROBINS-E assessments were done separately for the primary and secondary outcomes. For the primary outcome of mortality, 63 studies were judged to have high overall ROB [17–19, 21–39, 41–43, 45–52, 54–59, 61–63, 65, 67–85, 87], and 9 had some concerns overall ROB[20, 40, 44, 53, 60, 64, 66, 86, 88]. The studies with serious risk of bias predominantly had limitations arising from confounding and selection bias (Fig. 2).

For the secondary outcomes, BPD, IVH, NEC, ROP, Sepsis, VAP, and pulmonary haemorrhage, most studies were rated as having high ROB, predominantly due to concerns in the domains of confounding and selection bias. Individual study level and summary ROB assessments are presented in Supplemental file 1, Figure S1–S15.

### Primary outcome

#### In-hospital mortality

**Fig. 3:**
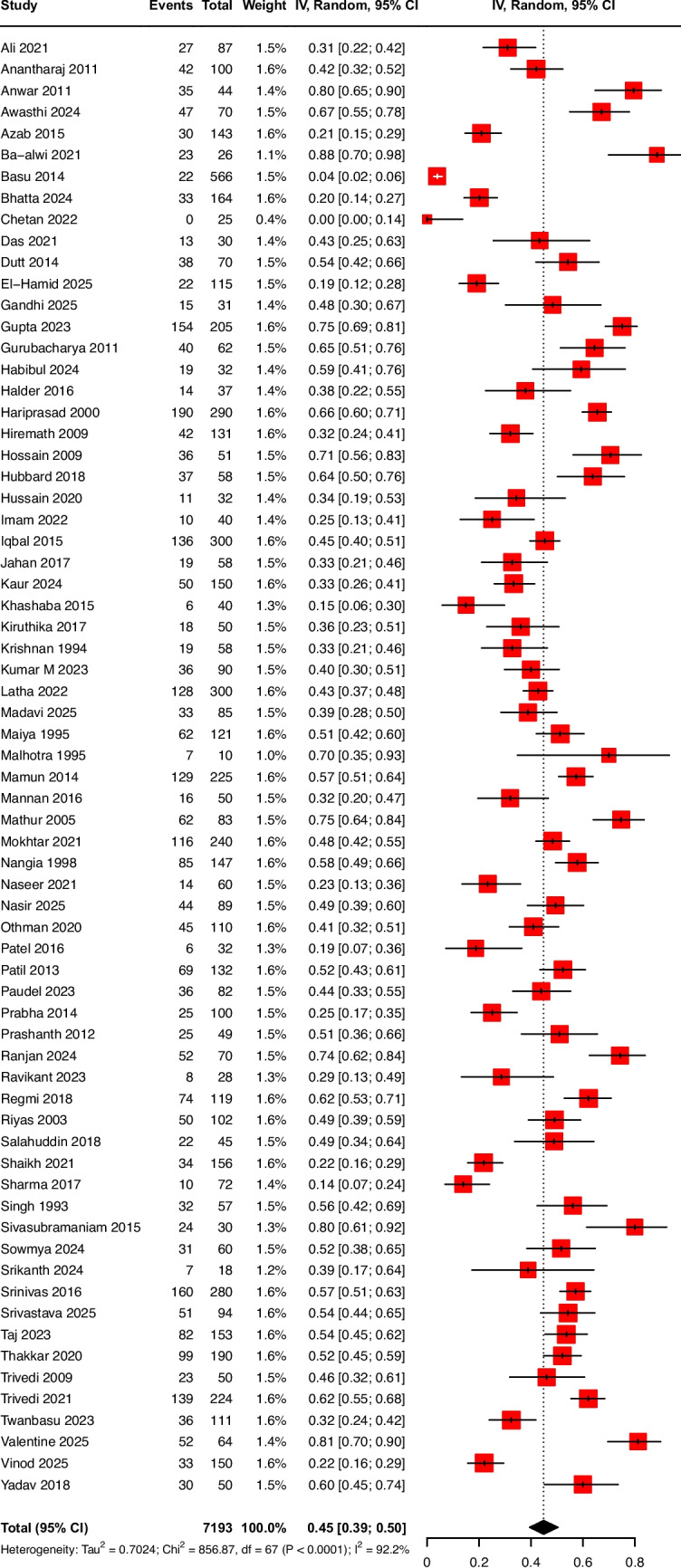
Forrest plot for pooled proportion of in-hospital mortality among neonates receiving mechanical ventilation

A total of 68 studies (7193 neonates) reported in-hospital mortality [17–84]. Mortality rate was 45% (95% CI, 39%–50%); CoE being very low, downgraded by two levels for ROB and one level for inconsistency (Table 1, Fig. 3). Of the 68 studies, only one study was from a low-income country [17].

**Table 1:** GRADE certainty of evidence for the primary and secondary outcomes based on pooled proportions and narrative evidence

Certainty assessment							Summary of findings				
Participants (studies) Follow-up	Risk of bias	Inconsistency	Indirectness	Imprecision	Publication bias	Overall certainty of evidence	Study event rates (%)		Relative effect (95% CI)	Anticipated absolute effects	
							With [comparison]	With ventilation		Risk with [comparison]	Risk difference with ventilation
In-hospital mortality											
7193 (72 non-randomised studies)	Very serious^a^	Serious^b^	Not serious	Not serious	None	⨁◯◯◯ Very low^a,b^	Baseer et al. [85] prospectively enrolled neonates with respiratory distress and assessed outcomes by aetiology. The study reported an unadjusted odds ratio of 29.3 (95% CI, 8.65–141) and was judged to be at high ROB, as it did not account for confounding factors Mukhopadhyay et al. [86], in a prospective study, included very low birth weight neonates to assess risk factors for mortality and identified IMV as an independent risk factor (aOR 4.10; 95% CI, 1.64–10.28). This study was judged to have some concerns due to the possibility of residual confounding Shah et al. [87] retrospectively evaluated the association between ventilation and mortality in neonates with sepsis and found that IMV was associated with increased mortality (OR 7.73; 95% CI, 2.40–24.91). This study did not account for confounding and was judged to be at high ROB Zakariya et al. [88], in a prospective study from India, identified IMV as an independent risk factor associated with mortality (aOR 3.58; 95% CI, 1.16–11.07) and was judged to have some concerns due to residual confounding				
Bronchopulmonary dysplasia											
1206 (15 non-randomised studies)	Very serious^c^	Serious^b^	Not serious	Not serious	None	⨁◯◯◯ Very low^b,c^	Patel et al. [92], in a case–control study from India, assessed risk factors for BPD, and reported an odds ratio of 28.6 (95% CI, 13–62.8) among ventilated preterm neonates born at less than 32 weeks’ gestation Mishra et al. [93], a case–control study from India, reported an unadjusted odds ratio of 6.18 (95% CI, 2.15–17.8) for BPD in neonates born at less than 32 weeks’ gestation Both studies were judged to be at high ROB as the studies did not account for known confounders				
Intraventricular haemorrhage											
1503 (16 non-randomised studies)	Very serious^d^	Serious^b^	Not serious	Not serious	Strongly suspected^e^	⨁◯◯◯ Very low^b,d,e^					
Necrotising enterocolitis											
378 (7 non-randomised studies)	Very serious^f^	Serious^b^	Not serious	Not serious	None	⨁◯◯◯ Very low^b,f^	Gitau et al. [99], in a retrospective study from Kenya, assessed risk factors for NEC stage ≥ 2 and identified IMV as an independent risk factor (OR 2.17; 95% CI, 1.24–3.79). The study was judged to be of some concerns				
Retinopathy of prematurity											
1148 (17 non-randomised studies)	Very serious^g^	Serious^h^	Not serious	Not serious	None	⨁◯◯◯ Very low^g,h^	Gaber et al. [100], in a cross-sectional retrospective study from Egypt, assessed the risk factor for any stage ROP in preterm neonates below 34 weeks and found an increased odds of 13.6 (95% CI, 5.6–33.1).The study was judged to be of high ROB for confounding and patient selection				
Ventilatory associated pneumonia											
2135 (22 non-randomised studies)	Very serious^i^	Serious^b^	Not serious	Not serious	Strongly suspected^j^	⨁◯◯◯ Very low^b,i,j^					
Sepsis											
3638 (36 non-randomised studies)	Very serious^k^	Serious^b^	Not serious	Not serious	None	⨁◯◯◯ Very low^b,k^	Rani et al. [130 reported higher odds of healthcare-associated infection among ventilated neonates (OR 2.11; 95% CI, 1.24–2.59). The study was judged to be at high ROB as the study did not account for confounders				
Pulmonary haemorrhage											
2170 (22 non-randomised studies)	Very serious^l^	Serious^b^	Not serious	Not serious	Strongly suspected^m^	⨁◯◯◯ Very low^l,m^					

*CI*, confidence interval; *RR,* risk ratio; *ROB*, risk of bias; *OR*, odds ratio; *aOR*, adjusted odds ratio; *BPD*, bronchopulmonary dysplasia; *IMV*, invasive mechanical ventilation; *ROP*, retinopathy of prematurity

*Explanations*

^a^Risk of bias assessment showed high risk of bias in 63 of 72 studies, primarily due to confounding and selection of participants

^b^CoE was downgraded by one level, as study estimates were consistent with overlapping confidence intervals

^c^Risk of bias showed a high risk of bias in all 15 studies, primarily due to the selection of participants and confounding

^d^Risk of bias assessment showed high risk of bias in 15 of 19 studies, primarily due to confounding and selection of participants

^e^Publication bias was suspected based on funnel plot asymmetry (Kendall’s tau =  − 0.5; *p* = 0.0006)

^f^Risk of bias assessment showed a high risk of bias in 6 of 7 studies, primarily due to confounding

^g^Risk of bias assessment showed a high risk of bias in 10 of 17 studies due to confounding and the selection of participants

^i^.isk of bias assessment showed a high risk of bias in 19 of 22 studies, primarily due to the selection of participants

^j^Publication bias was suspected based on funnel plot asymmetry (Kendall’s tau =  − 0.4025; *p* = 0.0083)

^k^Risk of bias assessment showed a high risk of bias in 32 of 39 studies, primarily due to confounding, confounding and selection of participants

^l^Risk of bias assessment showed high risk of bias in 20 of 22 studies, primarily due to confounding

^m^Publication bias was suspected based on funnel plot asymmetry (Kendall’s tau =  − 0.552; *p* = 0.0003)

Four studies reported the association between IMV and mortality among neonates [85–88]. However, these estimates were not pooled because of clinical heterogeneity and differences in adjustment for confounders. In a prospective cohort study from Egypt, Baseer et al. included all neonates admitted with respiratory distress and reported higher odds of mortality among ventilated neonates (OR 29.3; 95% CI, 8.65–141) [85]. Mukhopadhyay et al., in a prospective study from India, reported IMV as an independent predictor of mortality after adjustment for birth weight, antenatal steroid exposure, and perinatal asphyxia (adjusted odds ratio (aOR) 4.10; 95% CI, 1.64–10.28) [86]. Two additional studies from India evaluated predictors of mortality in neonates with sepsis and reported an increased risk of death associated with IMV, with an adjusted odds ratio of 3.58 (95% CI, 1.16–11.07) [88] and an unadjusted odds ratio of 7.73 (95% CI, 2.40–24.91) [87].

### Secondary outcomes

#### BPD

**Fig. 4:**
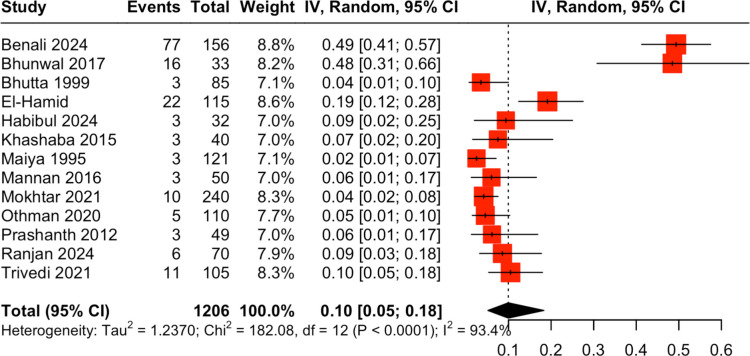
Forrest plot for pooled proportion of bronchopulmonary dysplasia (BPD) among ventilated neonates

Thirteen studies [29, 33, 44, 51, 53, 59, 64, 65, 80, 89–91] including 1206 neonates, reported the outcome BPD, with a pooled rate of 10% (95% CI, 5%–18%); CoE being very low, downrated by two levels for ROB and by one level for inconsistency (Fig. 4).

Two eligible studies reported the association of BPD with IMV [92, 93]. Patel et al., in a case–control study from India, assessed risk factors for BPD, defined as the need for oxygen supplementation at 36 weeks’ postmenstrual age, and reported an OR of 28.6 (95% CI, 13–62.8) among ventilated very preterm neonates [92]. Mishra et al., in another case–control study from India, reported an unadjusted OR of 6.18 (95% CI, 2.15–17.8) for BPD in very preterm neonates [93].

### IVH

*Any-grade IVH:* Sixteen studies [27, 33, 34, 47, 48, 53, 56, 64, 65, 68, 74, 76, 81, 91, 94, 95] including 1503 neonates reported any-grade IVH, the rate being 10% (95% CI, 5%–19%) (Figure S1 in Supplemental file 2).

For studies reporting IVH, CoE was rated very low, downrated by two levels for ROB, and one level each for inconsistency, and publication bias (Table 1).

Three studies [96–98] reported aORs for any-grade IVH, with aOR of 3.94 (95% CI, 0.14–108.9); CoE for the association of any-grade IVH with IMV was rated very low (Figure S2 and Table S2 in Supplemental file 2).

### NEC

*Any-stage NEC*: Five studies [33, 59, 60, 64, 65] including 293 neonates reported any-stage NEC, with a pooled incidence of 17% (95% CI, 7%–38%) (Figure S3 in Supplemental file 2).

*NEC stage* ≥ *2:* One study (85 neonates) [91], reported NEC stage ≥ 2, with an incidence rate of 6% (95% CI, 2%–13%) (Figure S3 in Supplemental file 2).

The CoE for NEC was very low, downgraded by two levels for ROB, and one level for inconsistency (Table 1).

Gitau et al., in a retrospective study from Kenya, assessed risk factors for NEC stage ≥ 2 among preterm neonates below 32 weeks and identified IMV as an independent risk factor (OR 2.17; 95% CI, 1.24–3.79) [99].

### ROP

*Any-stage ROP:* 13 studies (1065 neonates) [33, 64, 65, 91, 101–109] reported any-stage ROP, with a pooled incidence of 33% (95% CI, 20%–49%) (Figure S4 in Supplemental file 2).

*ROP requiring treatment:* Three studies enrolling 83 neonates assessed the outcome ROP requiring treatment [110–112], with a pooled incidence of 32% (95% CI, 6%–77%) (Figure S4 in Supplemental file 2).

The CoE for ROP was rated very low, downgraded by two levels for ROB and one level for inconsistency (Table 1).

Gaber et al., in a cross-sectional study from Egypt, assessed the risk factor for any stage ROP in preterm neonates below 34 weeks and found an increased odds ratio of 13.6 (95% CI: 5.6–33.1) [100].

### VAP

Twenty-two studies [22, 29, 42, 45, 47, 50, 51, 53–55, 59, 64, 67, 74, 79, 81, 83, 113–117] including 2135 neonates reported VAP rate of 21% (95% CI, 14%–29%); CoE being very low, downrated by two levels for ROB, one level for inconsistency and one level for publication bias (Figure S5 in Supplemental file 2).

### Sepsis

Thirty-five studies including 3638 neonates [15, 27, 33, 34, 42, 45, 48, 50, 51, 53, 55–57, 59, 61, 63–65, 68, 70, 76, 77, 79, 81, 91, 118–127] reported the rates of sepsis among ventilated neonates, with a pooled incidence of 32% (95% CI, 25%–40%); CoE was very low, downrated by two levels for ROB and one for inconsistency (Figure S6 in Supplemental File 2).

Four studies reported an association between IMV and sepsis. Three studies [16, 128, 129] were pooled as they reported adjusted estimates. The pooled odds ratio was 4.93 (95% CI: 2.98–8.18); CoE for the association of sepsis with ventilation was rated as moderate (Figure S7 and Table S2 in Supplemental file 2).

Rani et al., a study from India, studied risk factors of healthcare-associated infection in neonates admitted to NICU and reported higher odds of healthcare-associated infection among ventilated neonates (OR 2.11; 95% CI, 1.24–2.59) [130].

### Pulmonary haemorrhage

Twenty-two studies [19, 27, 34, 42, 43, 45, 47, 50, 51, 53, 55–59, 61, 63, 64, 67, 68, 79, 81] including 2170 neonates reported pulmonary haemorrhage, with a pooled incidence of 9% (95% CI, 6%–14%); CoE was very low, downgraded by two levels for ROB and one level each for inconsistency and publication bias (Figure S8 in Supplemental file 2).

#### Subgroup analyses

Subgroup analyses were performed by country, geographical region, gestational age, and underlying aetiology. The pooled mortality proportion differed significantly across countries (test for subgroup differences: *Q* = 66.79, df = 8, *p* < 0.0001), geographical region (*Q* = 20.60, df = 3, *p* = 0.0001) and by aetiology (*Q* = 20.99, df = 2, *p* < 0.0001), but not by gestational age (*Q* = 1.31, df = 2, *p* = 0.5191). Details of individual subgroup analyses are provided in Appendix 2 and Figures S9–S12 in Supplemental file 2.

#### Sensitivity analyses

After exclusion of studies with small sample sizes (< 50 neonates) (51 studies, 6644 neonates) [17–19, 21, 22, 24, 25, 28, 29, 31, 32, 35–38, 41–43, 45–51, 53–59, 61–63, 65, 67, 68, 70–72, 74, 76–84], the pooled in-hospital mortality rate was 45% (95% CI, 39%–51%) (*Q* = 0.01, df = 1, *p* = 0.941) (Figure S13 in Supplemental file 2). Exclusion of studies with high ROB, studies with some concerns (7 studies, 283 neonates), showed a pooled mortality rate of 35% (95% CI, 17%–58%) (*Q* = 1.31, df = 1, *p* = 0.253) [20, 40, 44, 53, 60, 64, 66] (Figure S14 in Supplemental file 2). Studies recruiting neonates before 2010 [19, 20, 28, 32, 34–37, 46, 49, 51, 52, 54, 56, 64, 68, 72] (17 studies, 1637 neonates) reported a significantly higher pooled mortality rate of 55% (95% CI, 48%–63%) compared with studies conducted from 2010 onwards (51 studies, 5627 neonates), which reported a pooled mortality rate of 43% (95% CI, 36%–50%) (*Q* = 6.30, df = 1, *p* = 0.0121) (Figure S15 in Supplemental file 2).

#### Publication bias

Publication bias was assessed for the outcomes in-hospital mortality, ROP, and sepsis, for which no significant bias was adjudged. However, publication bias was suspected for the outcomes BPD, IVH, NEC, VAP, and pulmonary haemorrhage (Figures S16–S23 in Supplemental file 2).

## Discussion

The results of this systematic review and meta-analysis demonstrate a high burden of mortality among ventilated neonates in LRS, with the rate of in-hospital mortality ranging from 39 to 50%. In addition, ventilated neonates had a considerable burden of major morbidities, including BPD, ROP, IVH, NEC, pulmonary bleeding, sepsis, and VAP. To our knowledge, this is the first systematic review to comprehensively evaluate the mortality and morbidities among ventilated neonates in LRS.

A direct comparison with outcomes from high-income countries (HICs) is not feasible due to scarcity of data on ventilated neonates in HICs. Nevertheless, available benchmarks from HICs highlight a profound disparity. For instance, in a large population-based cohort study from the USA, neonatal mortality was exceptionally low at 0.02%, with neonatal intensive care unit (NICU) admission rates of 4.4% and IMV rates of 3% [134]. Even the mortality rate amongst sicker neonates born at gestational age of 27–32 weeks was 3% as reported in a study from the UK [135]. Similar low rates of all-cause mortality among very low birth weight neonates were reported from 11 HICs, which ranged from 4.2% to 13.5% [136]. There could be several reasons for the burden gap between LRS and HICs.

In addition, interpretation and direct comparison of mortality rates are constrained by significant differences in reporting frameworks and baseline patient characteristics. In HICs, neonatal outcomes are systematically captured through large neonatal networks or population-based databases, allowing for benchmarking, longitudinal trend analysis, and the impact of interventions. In contrast, evidence from LRS is predominantly derived from small single-centre studies with a serious risk of overall bias, restricting meaningful assessment of healthcare interventions and their impact. Notably, the cohorts in the included LRS studies consisted of relatively more mature neonates, yet mortality was markedly higher. Similarly, the observed lower rates of prematurity-related morbidities in the included studies from LRS are likely explained by the difference in NICU population characteristics and by competing risk, as mortality precludes the manifestation of these late-occurring morbidities in preterm survivors.

The results of our systematic review indicate that, even with IMV, mortality remains substantially high. This only reiterates that appropriate ancillary systems of care are also vital for improving survival amongst neonates requiring IMV. IMV or similar resource-intensive healthcare interventions should therefore not be viewed as ‘silver bullets’, but rather as one part of an optimised continuum of neonatal care. Such an approach, as evident from the evolution of neonatal care in HICs, would likely have a significant impact, translating into better neonatal outcomes for this cohort in LRS [2]. In line with the past few decades, large-scale implementation of policies aiming at strengthening basic maternal and newborn care, including improved accessibility to oxygen therapy and continuous positive airway pressure (CPAP), has occurred [7, 137]. While these interventions benefit many neonates, a subset will inevitably require timely escalation to invasive ventilation. Centres providing IMV should be equipped with the capacity to anticipate, monitor, and manage ventilation-associated complications. Scaling up care for small and sick newborns of which provision for IMV is essential to achieve the SDG goal for neonatal mortality by 2030 [138]. Capacity building should extend beyond ventilator availability to include teaching and training of healthcare providers, infection prevention and antimicrobial stewardship, ROP screening and treatment services, respiratory support for evolving BPD, point-of-care ultrasonography for IVH screening, and access to paediatric surgical services for management of NEC. In addition, strengthening referral and transport systems and regionalisation of neonatal intensive care are vital [5]. Finally, there is a critical need for high-quality, standardised neonatal data systems in LRS. Such systems are essential for evaluating interventions such as IMV, monitoring quality, and informing policy. Future research should also focus on long-term neurodevelopmental and respiratory outcomes among NICU graduates who required IMV.

This review represents the most comprehensive review on mortality and morbidity outcomes among ventilated neonates in LRS. In the absence of population-based or network-based cohorts, this review provides the best available evidence from these regions by incorporating data from multiple single-centre studies representing varying levels of care. The inclusion of core neonatal outcomes, outcome-wise assessment of ROB with a validated tool, a GRADE approach to assess evidence certainty, and evaluation of the robustness of our findings through multiple sensitivity analyses are key strengths of our review. There were limitations as well. Ours was a pragmatic review that included single-centre observational studies with substantial heterogeneity and variable reporting quality. Due to limitations in existing evidence, we had to rely on unadjusted estimates reported in most of these studies. Our findings are not reflective of outcomes of very low birth weight (VLBW) and extremely low birth weight neonates, thus limiting the generalisability of the findings. Our findings do not establish causality of IMV with respect to the reported outcomes. Because this is a proportional meta-analysis designed to describe the overall burden of outcomes across a specified population, the calculated estimates inherently do not measure intervention effect sizes. Therefore, the reported outcome estimates should be interpreted strictly as pooled proportions derived from a clinically heterogeneous cohort of ventilated neonates and should not be attributed to specific underlying conditions or varying illness severities, such as sepsis. While the prespecified application of a random-effects model appropriately accounts for this baseline clinical variance across studies [139, 140], the resulting data remain descriptive and underscore the combined burden of the underlying pathology and the intensive care required. The pooled estimates were disproportionately influenced by studies from the Indian subcontinent, limiting applicability across all LRS. Finally, since there are no uniformly agreed upon criteria for classifying ‘LRS’, we predominantly used data from lower-income and lower-middle-income countries. Although we adhered to a predefined protocol, some deviations were needed. Studies comparing LISA and INSURE were excluded post hoc as duration of ventilation following surfactant administration is not standardised in the INSURE group [141, 142]. This exclusion is supported by a review that highlights the potential for misclassification when LISA and INSURE are considered equivalent [143]. In certain studies, INSURE includes either brief controlled invasive ventilation or unrestricted ventilation with varying pressure, volume, and duration. Certain predefined outcomes, including patent ductus arteriosus, could not be analysed due to inconsistent definitions and unclear causality. Other planned outcomes, such as duration of ventilation, long-term respiratory outcomes, and neurodevelopmental outcomes, could not be assessed, as the data were not available in the majority of studies and, in some, could not be extracted from the included studies.

## Conclusion

In low-resource settings, neonates receiving invasive mechanical ventilation have a disproportionately high mortality and morbidity relative to their clinical profile. While IMV is an essential component of the care of sick neonates and recent trends show modest improvement in mortality amongst ventilated neonates in LRS, its effectiveness is contingent on the simultaneous implementation of supportive systems. These must include integrated investments in workforce training, referral networks, and robust data infrastructure. Finally, the methodological limitations of studies from LRS, particularly non-standardised reporting of outcomes and unadjusted analyses, as highlighted in this review, should inform the design of future rigorous studies evaluating neonatal ventilation in low-resource contexts.

## Supplementary Information

Below is the link to the electronic supplementary material.

Supplementary file1 (DOCX 11562 KB)

Supplementary file2 (DOCX 19308 KB)

## Data Availability

The data used in this systematic review is from published literature available publicly. No additional data was obtained from the authors of the included studies. Data extraction sheet can be shared on request.

## Abbreviations

LRS: Low-resource settings
NMR: Neonatal mortality rate
SDG: Sustainable Development Goal
IMV: Invasive mechanical ventilation
PRISMA: Preferred Reporting Items for Systematic Reviews and Meta-analyses
CENTRAL: Cochrane Central Register of Controlled Trials
BPD: Bronchopulmonary dysplasia
IVH: Intraventricular haemorrhage
PVL: Periventricular leukomalacia
NEC: Necrotising enterocolitis
ROP: Retinopathy of prematurity
VAP: Ventilator-associated pneumonia
ROB: Risk of bias
ROBINS-E: Risk of Bias in Non-randomised Studies—of Exposure
CI: Confidence intervals
REML: Restricted maximum likelihood
GRADE: Grading of Recommendations Assessment, Development, and Evaluation
CoE: Certainty of evidence
OR: Odds ratios
aOR: Adjusted odds ratio
NICU: Neonatal intensive care unit
CPAP: Continuous positive airway pressure
VLBW: Very low birth weight
