# Supplementary material for: Prognosis of neonates receiving invasive mechanical ventilation in low-resource settings: a systematic review and prognostic meta-analysis
Source: Eur J Pediatr. 2026 May 7;185(6):360. doi: 10.1007/s00431-026-07016-z (PMC13152968; doi:10.1007/s00431-026-07016-z)

**Supplementary File 1**

**Title: Prognosis of Neonates Receiving Invasive Mechanical Ventilation in Low-Resource Settings: A Systematic Review and Prognostic Meta-Analysis**

Figure S1: Summary ROBINS-E risk of bias assessment for in-hospital mortality among ventilated neonates

Figure S2: ROBINS-E risk of bias assessment of individual studies for bronchopulmonary dysplasia (BPD) among ventilated neonates.

Figure S3: Summary ROBINS-E risk of bias assessment for bronchopulmonary dysplasia (BPD) in ventilated neonates

Figure S4: ROBINS-E risk of bias assessment of individual studies for intraventricular hemorrhage (IVH) among ventilated neonates.

Figure S5: Summary ROBINS-E risk of bias assessment for intraventricular hemorrhage (IVH) in ventilated neonates.

Figure S6: ROBINS-E risk-of-bias assessment of individual studies for necrotizing enterocolitis (NEC) among ventilated neonates.

Figure S7: Summary ROBINS-E risk of bias assessment for necrotizing enterocolitis (NEC) in ventilated neonates.

Figure S8: ROBINS-E risk of bias assessment of individual studies for retinopathy of prematurity (ROP) among ventilated neonates.

Figure S9: Summary ROBINS-E risk of bias assessment for retinopathy of prematurity (ROP) in ventilated neonates.

Figure S10: ROBINS-E risk of bias assessment of individual studies for ventilatory associated pneumonia (VAP) among ventilated neonates.

Figure S11: Summary ROBINS-E risk of bias assessment for ventilatory associated pneumonia (VAP) in ventilated neonates.

Figure S12: ROBINS-E risk of bias assessment of individual studies for sepsis among ventilated neonates.

Figure S13: Summary ROBINS-E risk of bias assessment for sepsis in ventilated neonates.

Figure S14: ROBINS-E risk of bias assessment of individual studies for pulmonary hemorrhage

among ventilated neonates.

Figure S15: Summary ROBINS-E risk of bias assessment for pulmonary hemorrhage

in ventilated neonates.

Figure S1: Summary ROBINS-E risk of bias assessment for in hospital mortality in ventilated neonates.


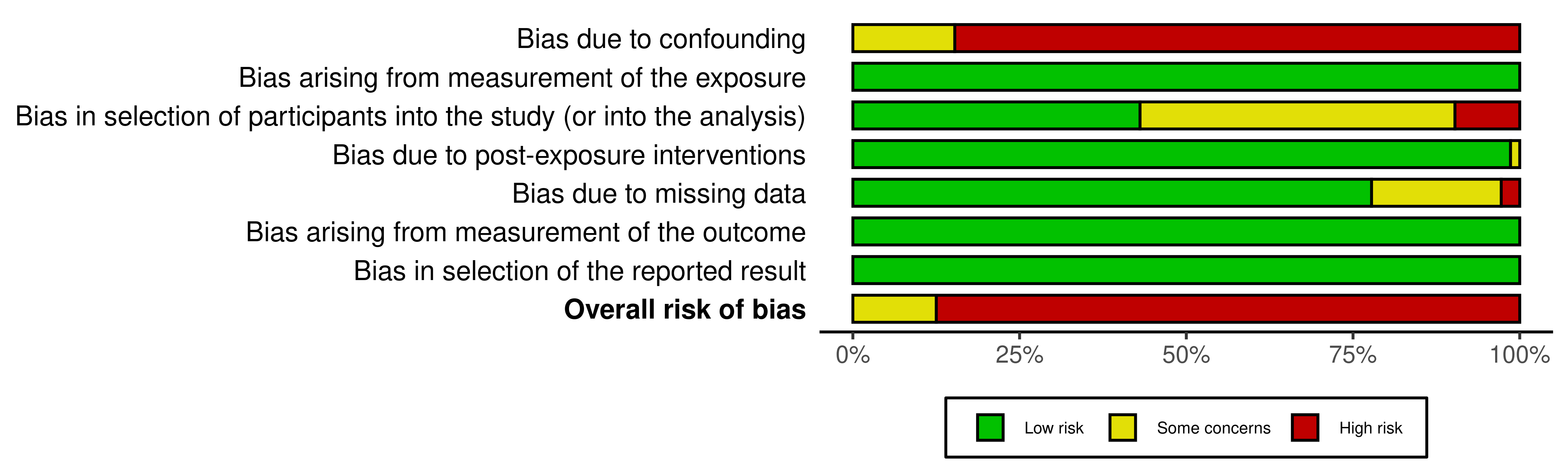


Figure S2: ROBINS-E risk of bias assessment of individual studies for bronchopulmonary dysplasia (BPD) among ventilated neonates.


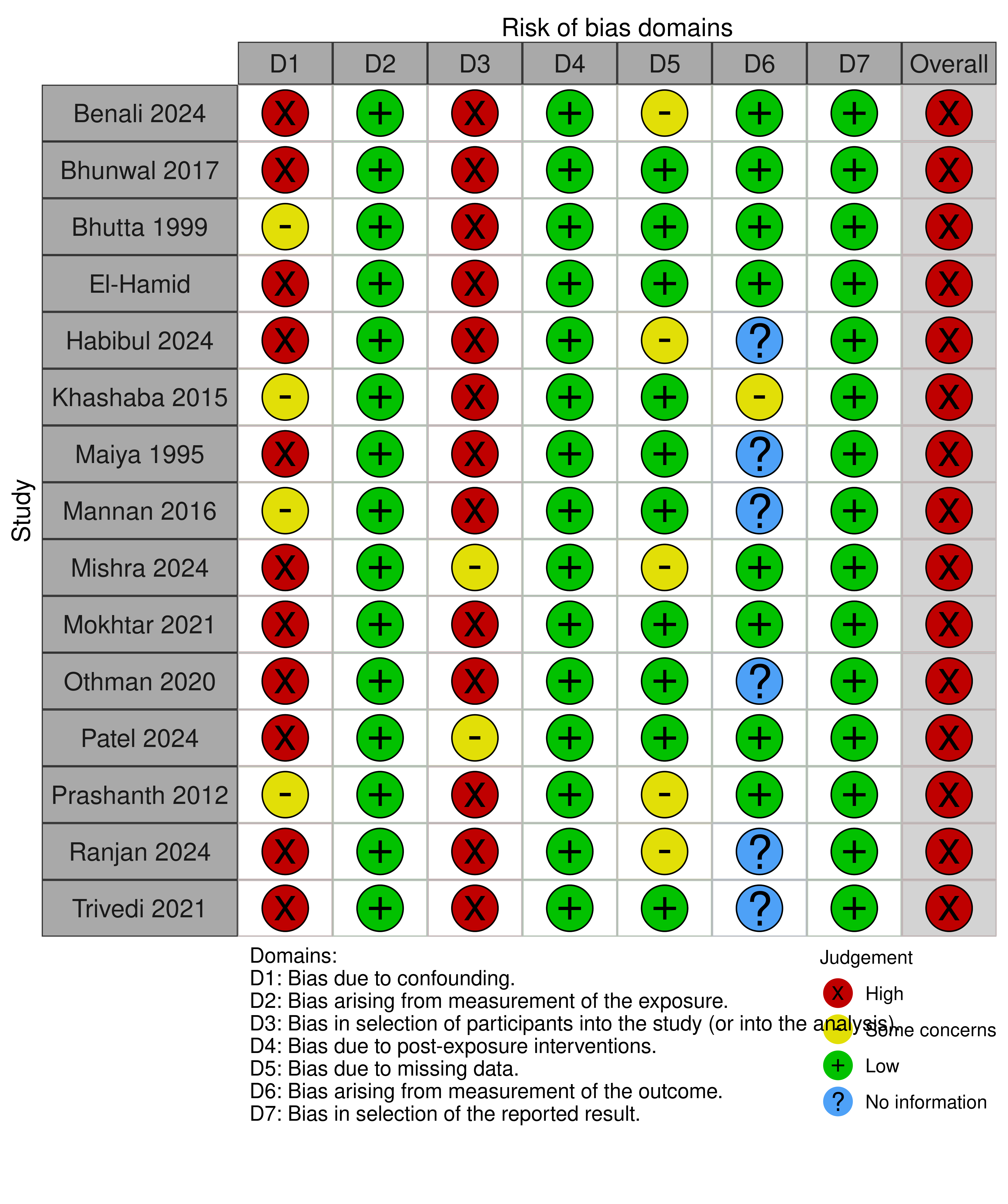


Figure S3: Summary ROBINS-E risk of bias assessment for bronchopulmonary dysplasia (BPD) in ventilated neonates


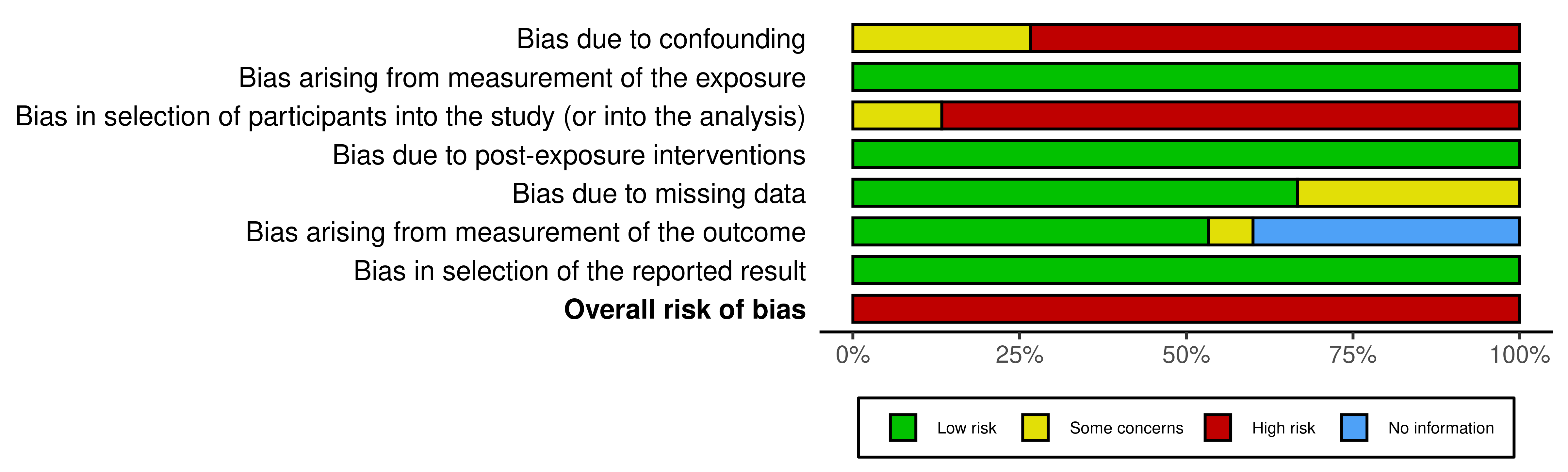


Figure S4: ROBINS-E risk of bias assessment of individual studies for intraventricular hemorrhage (IVH) among ventilated neonates.


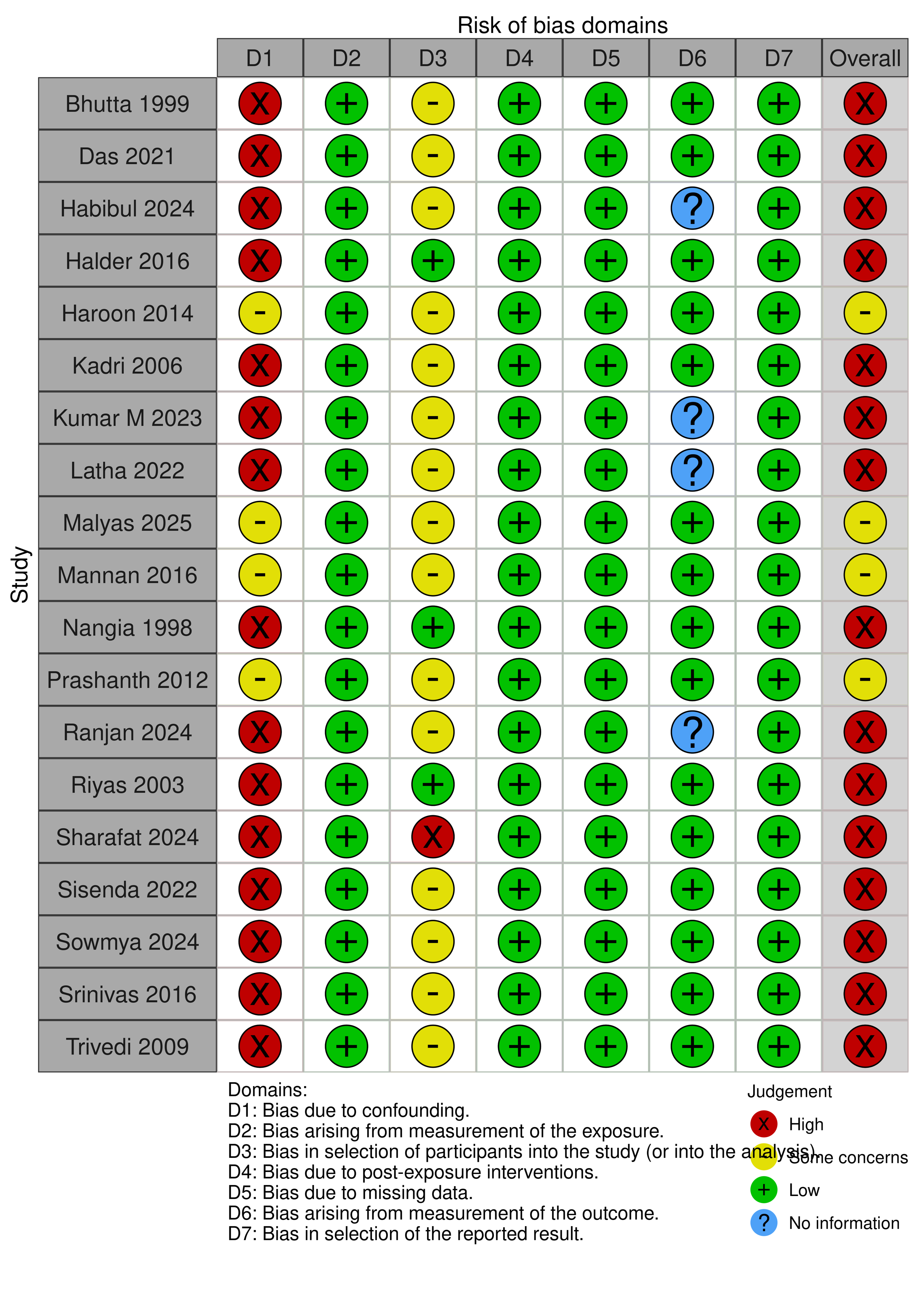


Figure S5: Summary ROBINS-E risk of bias assessment for intraventricular hemorrhage (IVH) in ventilated neonates.


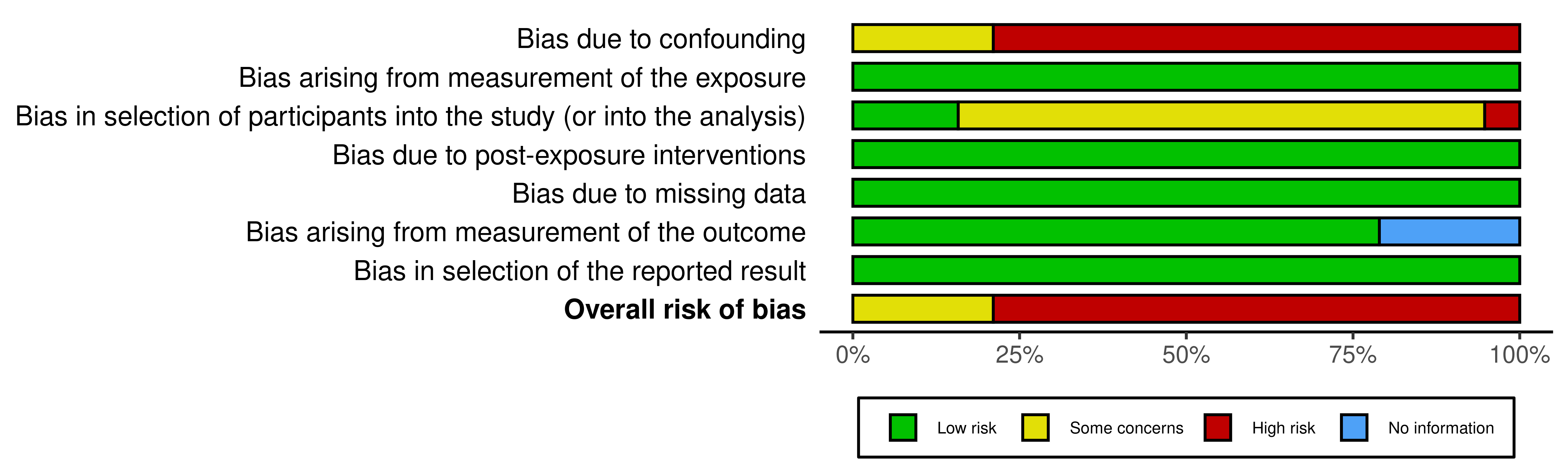


Figure S6: ROBINS-E risk-of-bias assessment of individual studies for necrotizing enterocolitis (NEC) among ventilated neonates


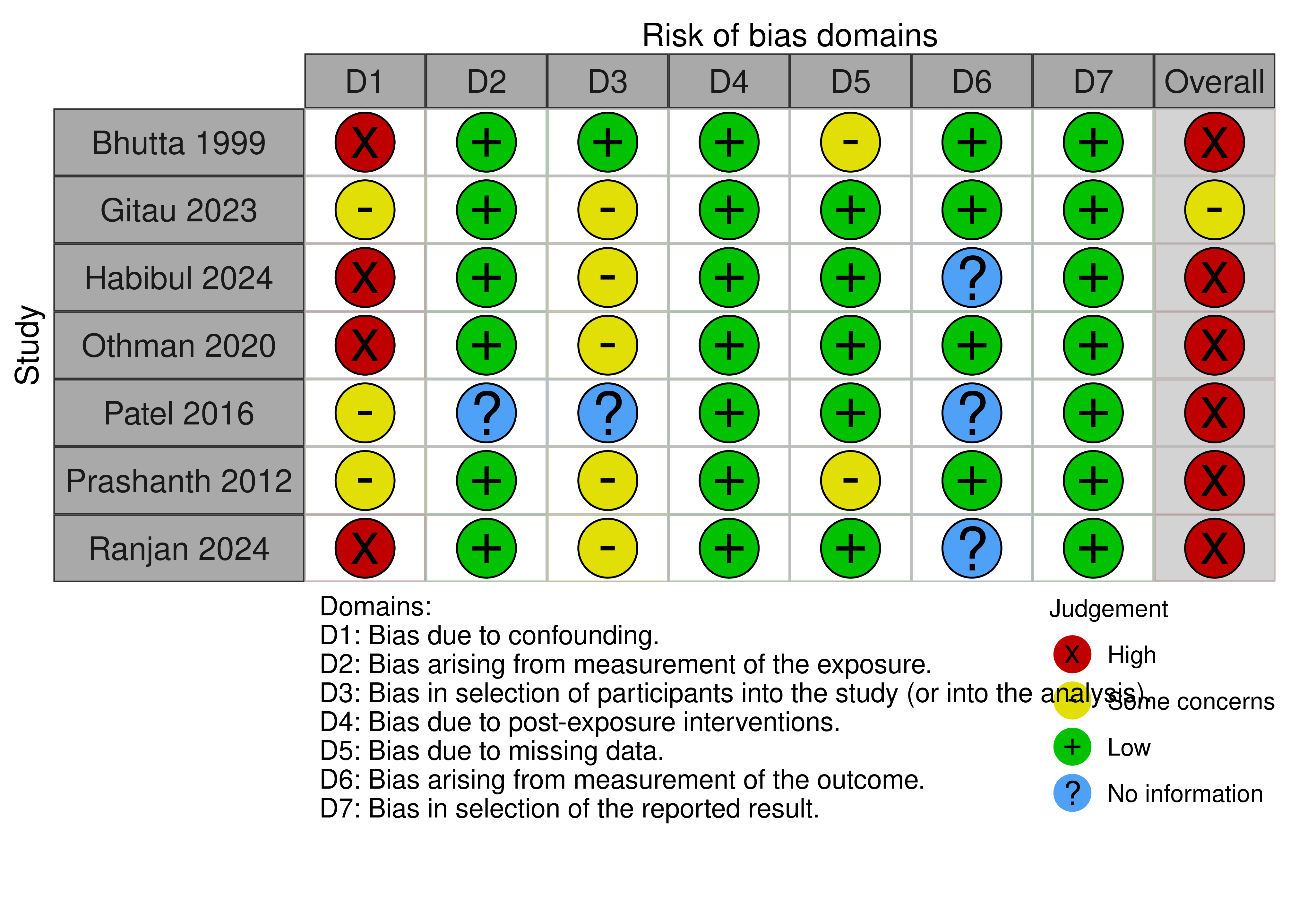


Figure S7: Summary ROBINS-E risk of bias assessment for necrotizing enterocolitis (NEC) in ventilated neonates.


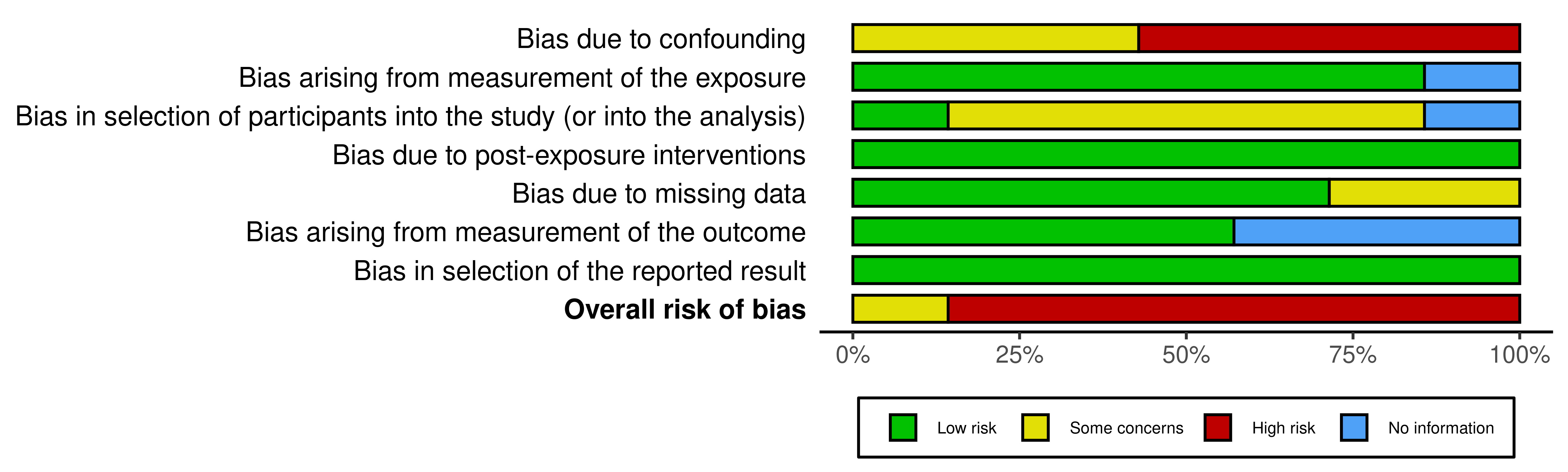


Figure S8: ROBINS-E risk of bias assessment of individual studies for retinopathy of prematurity (ROP) among ventilated neonates.


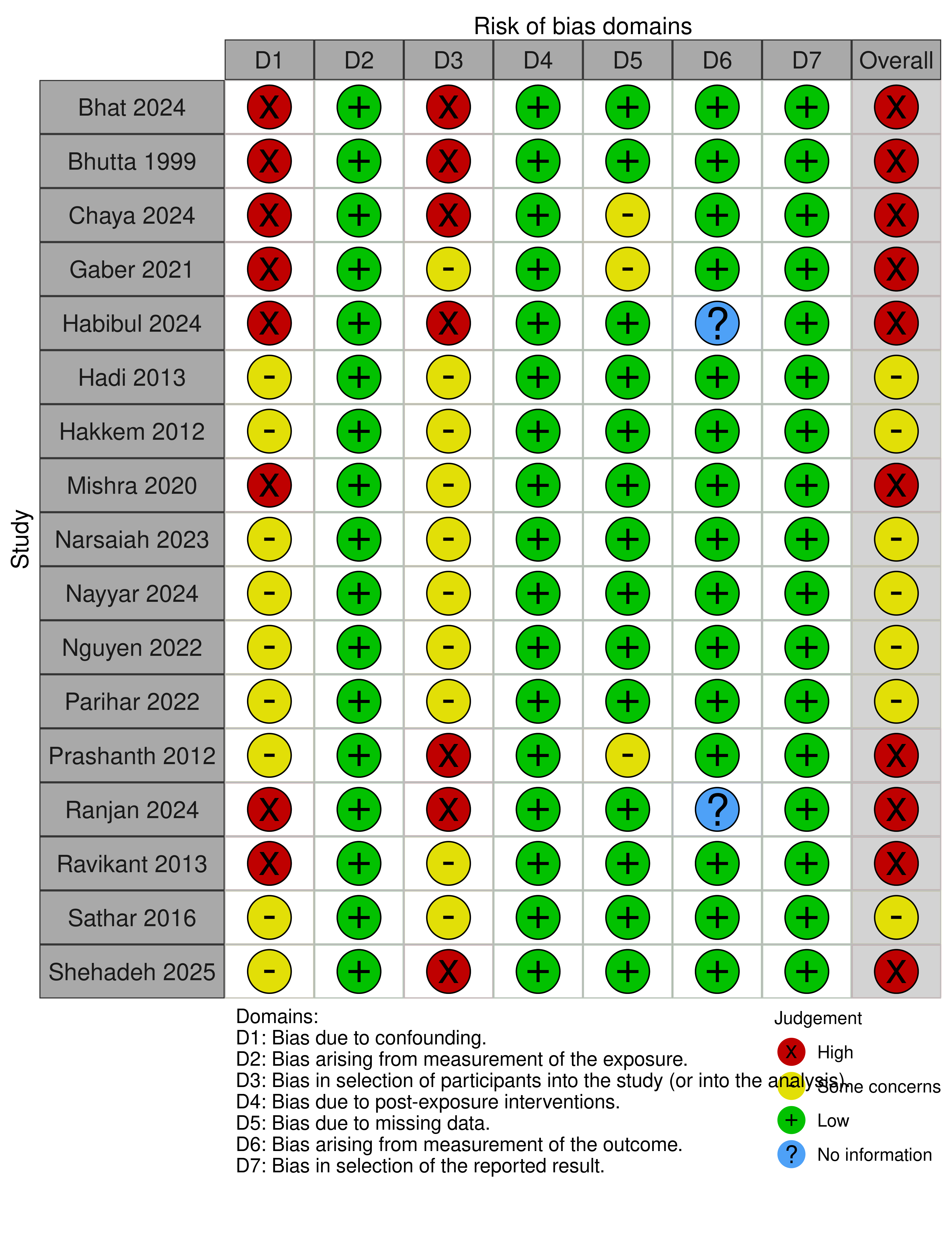


Figure S9: Summary ROBINS-E risk of bias assessment for retinopathy of prematurity (ROP) in ventilated neonates.


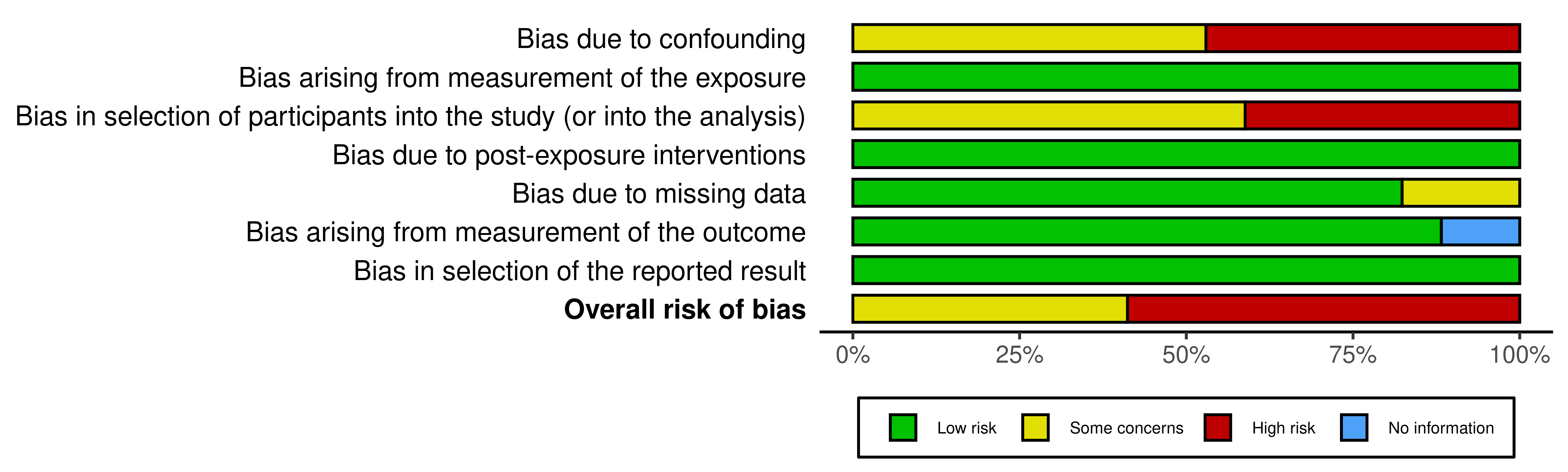


Figure S10: ROBINS-E risk of bias assessment of individual studies for ventilatory associated pneumonia (VAP) among ventilated neonates.


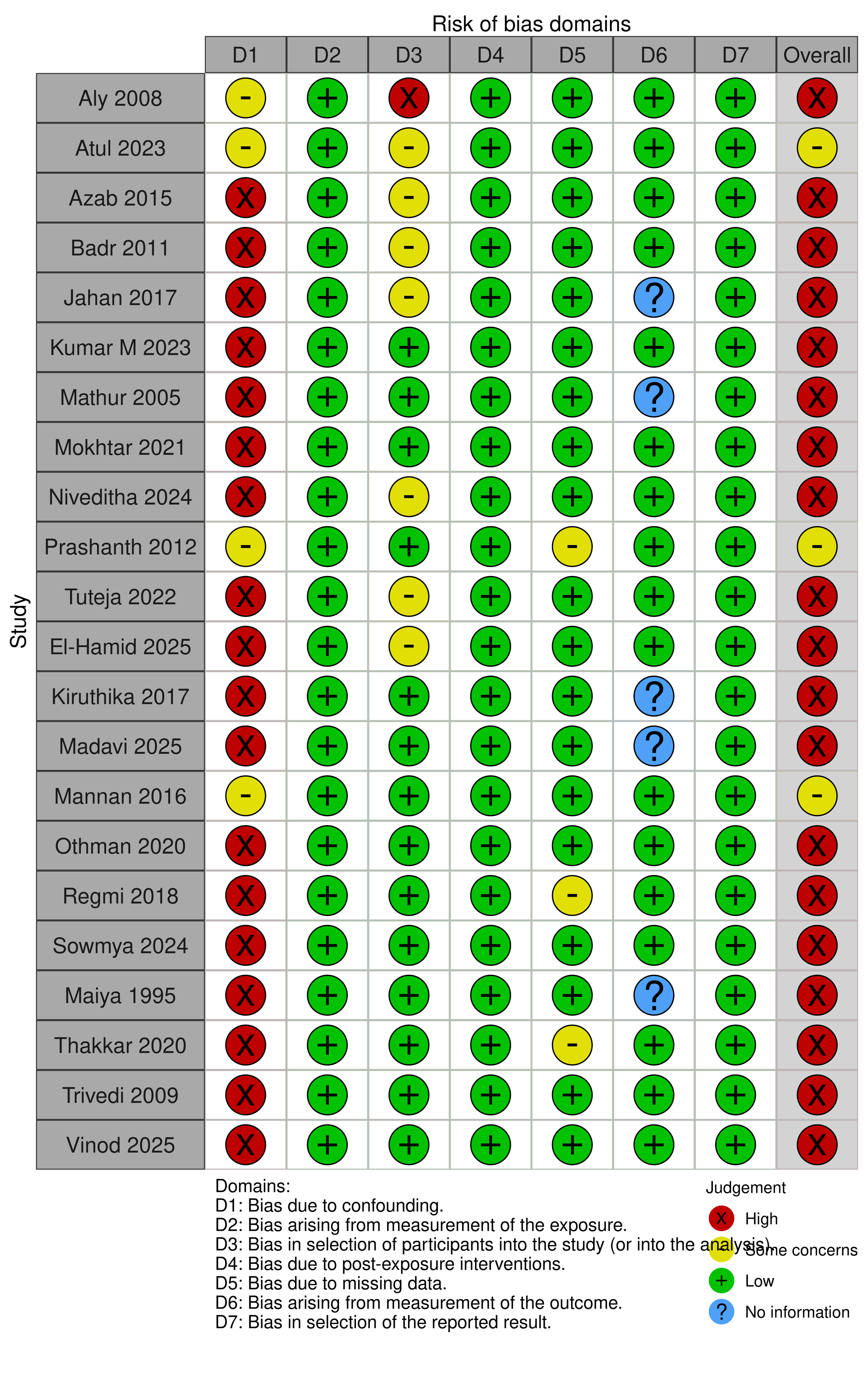


Figure S11: Summary ROBINS-E risk of bias assessment for ventilatory associated pneumonia (VAP) in ventilated neonates


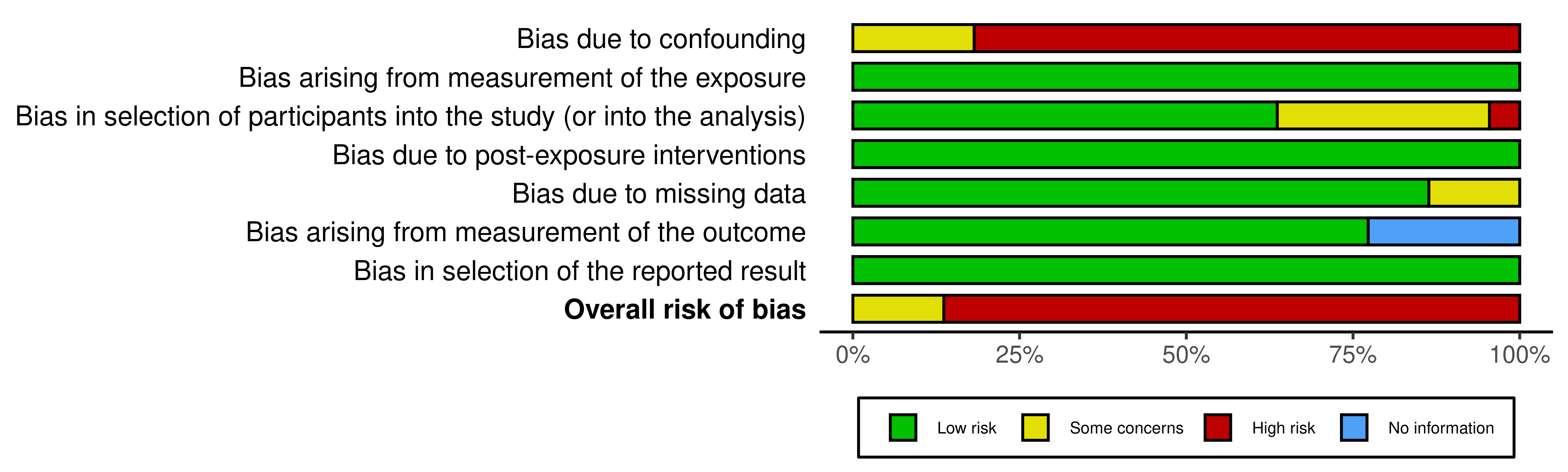


Figure S12: ROBINS-E risk of bias assessment of individual studies for sepsis among ventilated neonates.


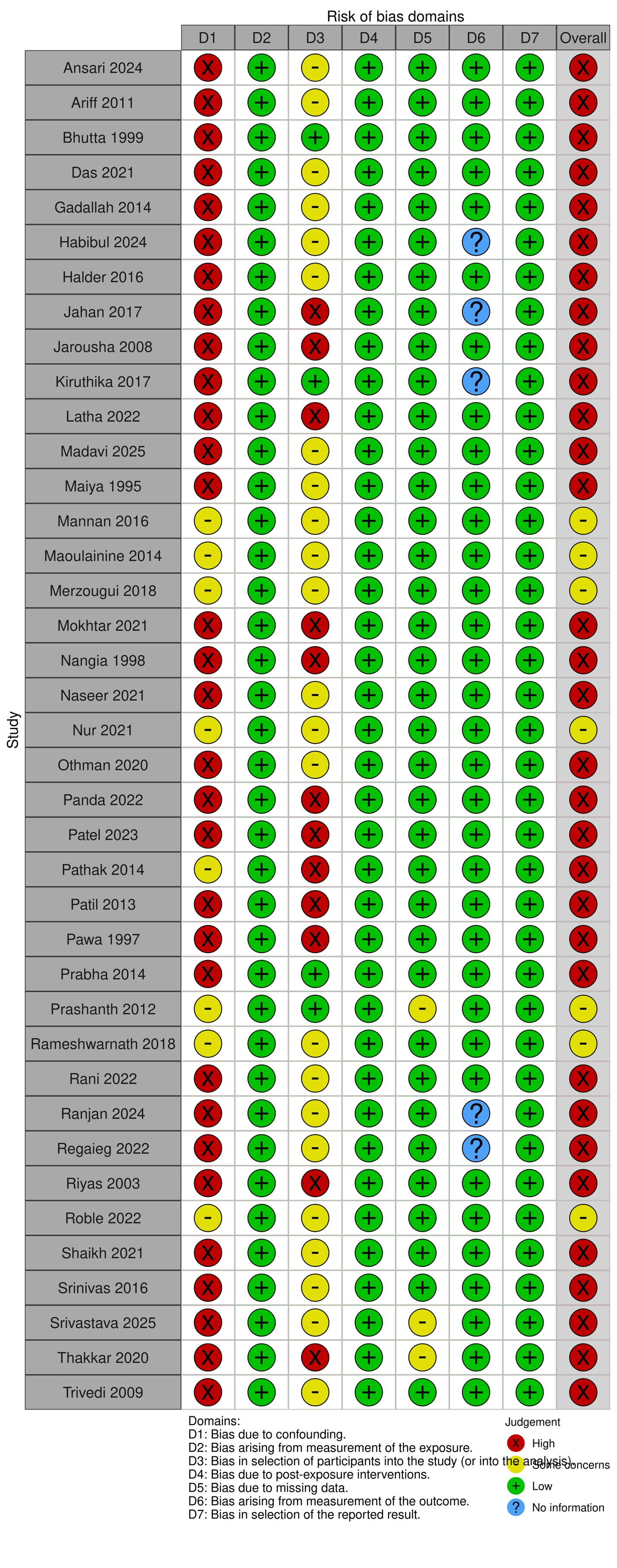


Figure S13: Summary ROBINS-E risk of bias assessment for sepsis in ventilated neonates.


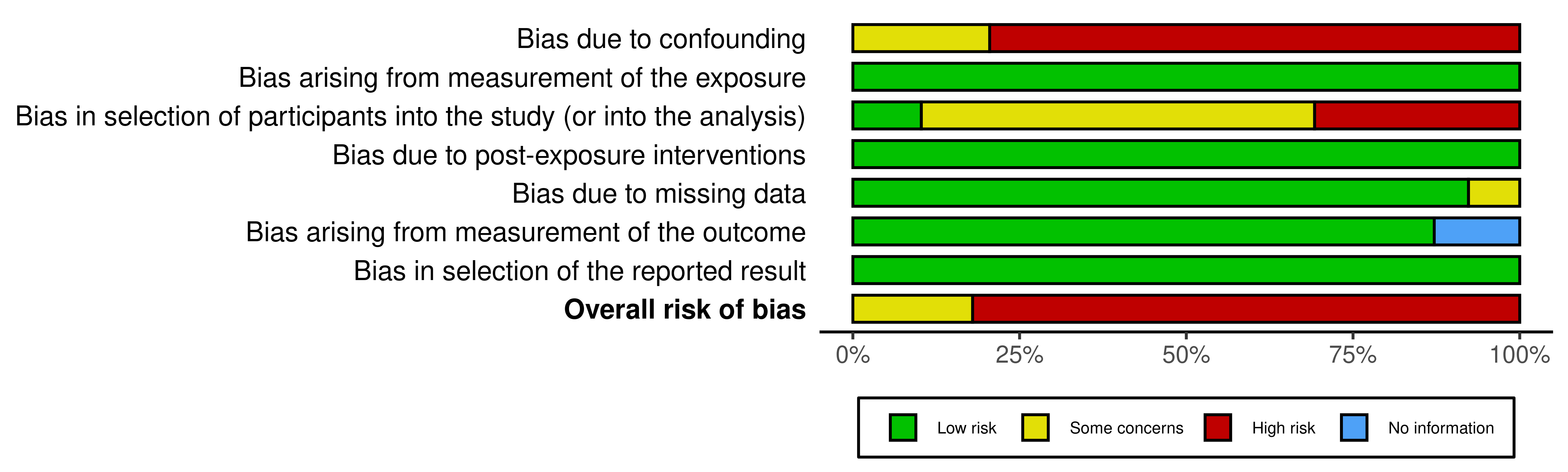


Figure S14: ROBINS-E risk of bias assessment of individual studies for pulmonary hemorrhage

among ventilated neonates.


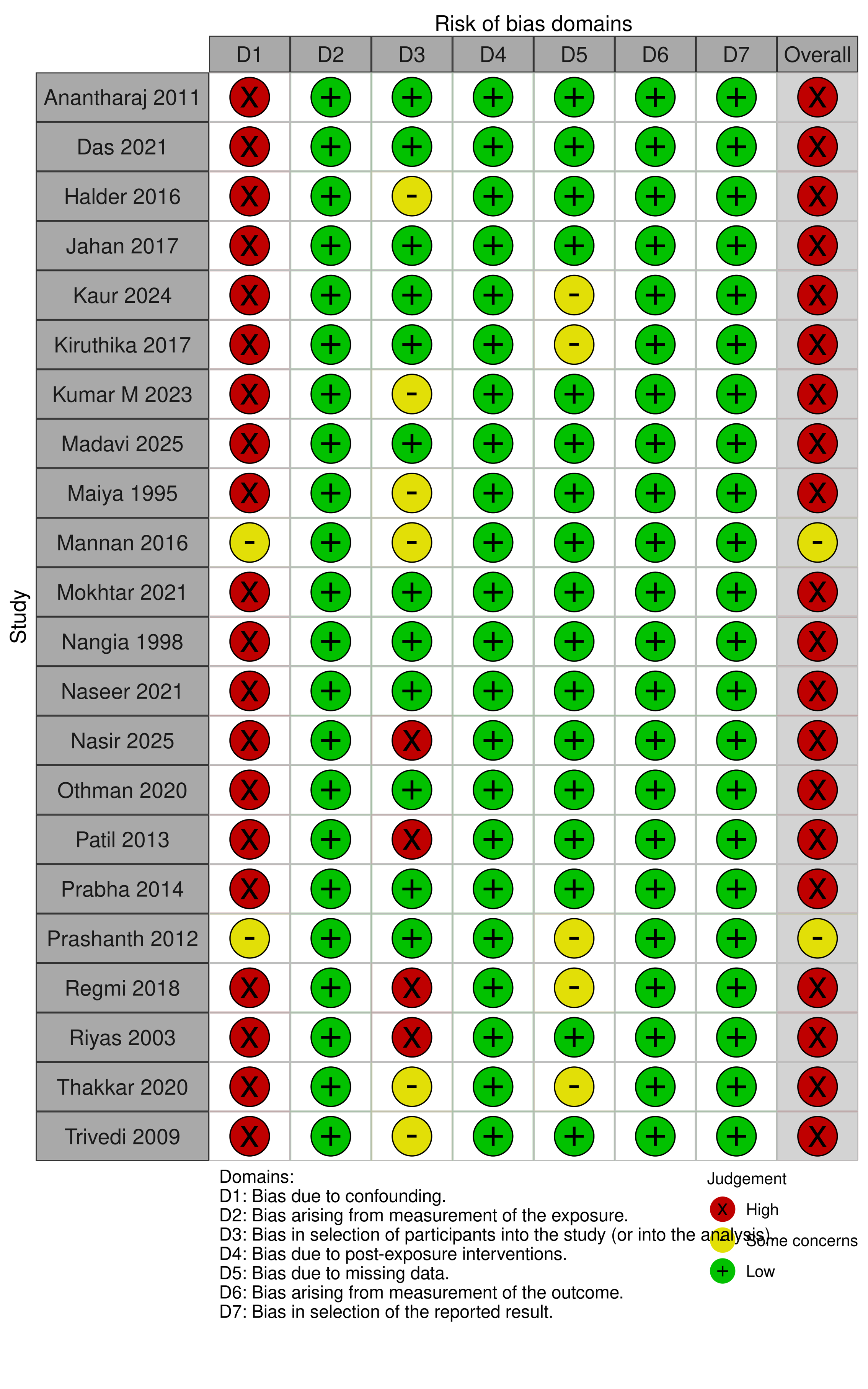


Figure S15: Summary ROBINS-E risk of bias assessment for pulmonary hemorrhage in ventilated neonates


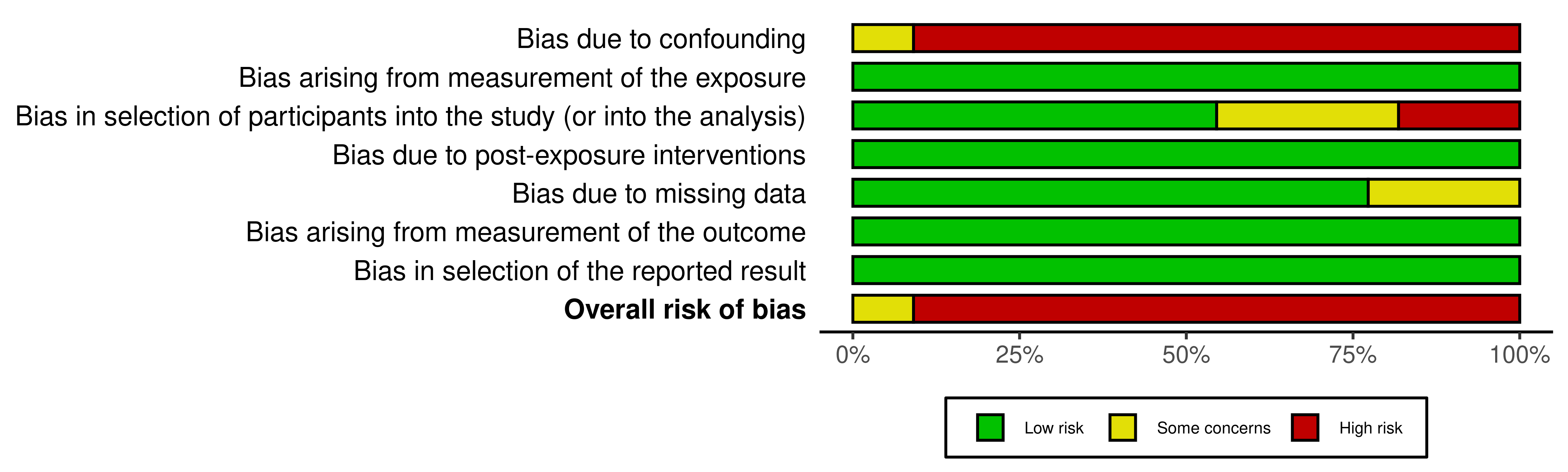

Supplement: Supplementary file 1 — Supplementary file1 (DOCX 11562 KB) [file 431_2026_7016_MOESM1_ESM.docx]
