# Supplementary material for: Prognosis of neonates receiving invasive mechanical ventilation in low-resource settings: a systematic review and prognostic meta-analysis
Source: Eur J Pediatr. 2026 May 7;185(6):360. doi: 10.1007/s00431-026-07016-z (PMC13152968; doi:10.1007/s00431-026-07016-z)
Supplement: Supplementary file 2 — Supplementary file2 (DOCX 19308 KB) [file 431_2026_7016_MOESM2_ESM.docx]

**Supplementary File 2**

**Title: Prognosis of Neonates Receiving Invasive Mechanical Ventilation in Low-Resource Settings: A Systematic Review and Prognostic Meta-Analysis**

**FIGURES**

Figure S1: Pooled proportion of intraventricular haemorrhage (IVH) as per grade among ventilated neonates.

Figure S2: Pooled adjusted odds ratio for any garde intraventricular haemorrhage (IVH) among ventilated neonates.

Figure S3: Pooled proportion of necrotising enterocolitis (NEC) among ventilated neonates, as per grade.

Figure S4: Pooled proportion of retinopathy of prematurity (ROP) among ventilated neonates as per stage.

Figure S5: Pooled proportion of ventilator associated pneumonia (VAP) among ventilated neonates.

Figure S6: Pooled proportion of sepsis among ventilated neonates.

Figure S7: Pooled adjusted odds ratio for sepsis among ventilated neonates.

Figure S8: Pooled proportion of pulmonary haemorrhage among ventilated neonates.

Figure S9: Subgroup analysis for pooled proportion of mortality before discharge among ventilated neonates, based on country of origin.

Figure S10: Subgroup analysis for pooled proportion of mortality before discharge among ventilated neonates, based on geographic region.

Figure S11: Subgroup analysis for pooled proportion of mortality before discharge among ventilated neonates, based on gestational age.

Figure S12: Subgroup analysis for pooled proportion of mortality before discharge among ventilated neonates, based on aetiology.

Figure S13: Sensitivity analysis for pooled proportions of mortality, after exclusion of studies with small sample size

Figure S14: Sensitivity analysis for pooled proportions of mortality, after exclusion of studies with high risk of bias

Figure S15: Sensitivity analysis for pooled proportions of mortality, for two epoch (Before 2010 vs After 2010)

Figure S16: Publication bias for mortality before discharge among ventilated neonates

Figure S17: Publication bias for bronchopulmonary dysplasia (BPD) among ventilated neonates

Figure S18: Publication bias for intraventricular haemorrhage (IVH) among ventilated neonates.

Figure S19: Publication bias for necrotising enterocolitis (NEC) among ventilated neonates

Figure S20: Publication bias for retinopathy of prematurity (ROP) among ventilated neonates

Figure S21: Publication bias for ventilator associated pneumonia (VAP) among ventilated neonates

Figure S22: Publication bias for sepsis among ventilated neonates

Figure S23: Publication bias for pulmonary haemorrhage among ventilated neonates

**TABLE**

Table S1: Characteristics of included studies.

Table S2: GRADE certainty of evidence for the secondary outcomes based on pooled adjusted odds ratio.

**Appendix 1:**  Literature Search

**Appendix 2:**  Subgroup analysis for primary outcome in-hospital mortality

Figure S1: Pooled proportion of intraventricular haemorrhage (IVH) as per grade among ventilated neonates.


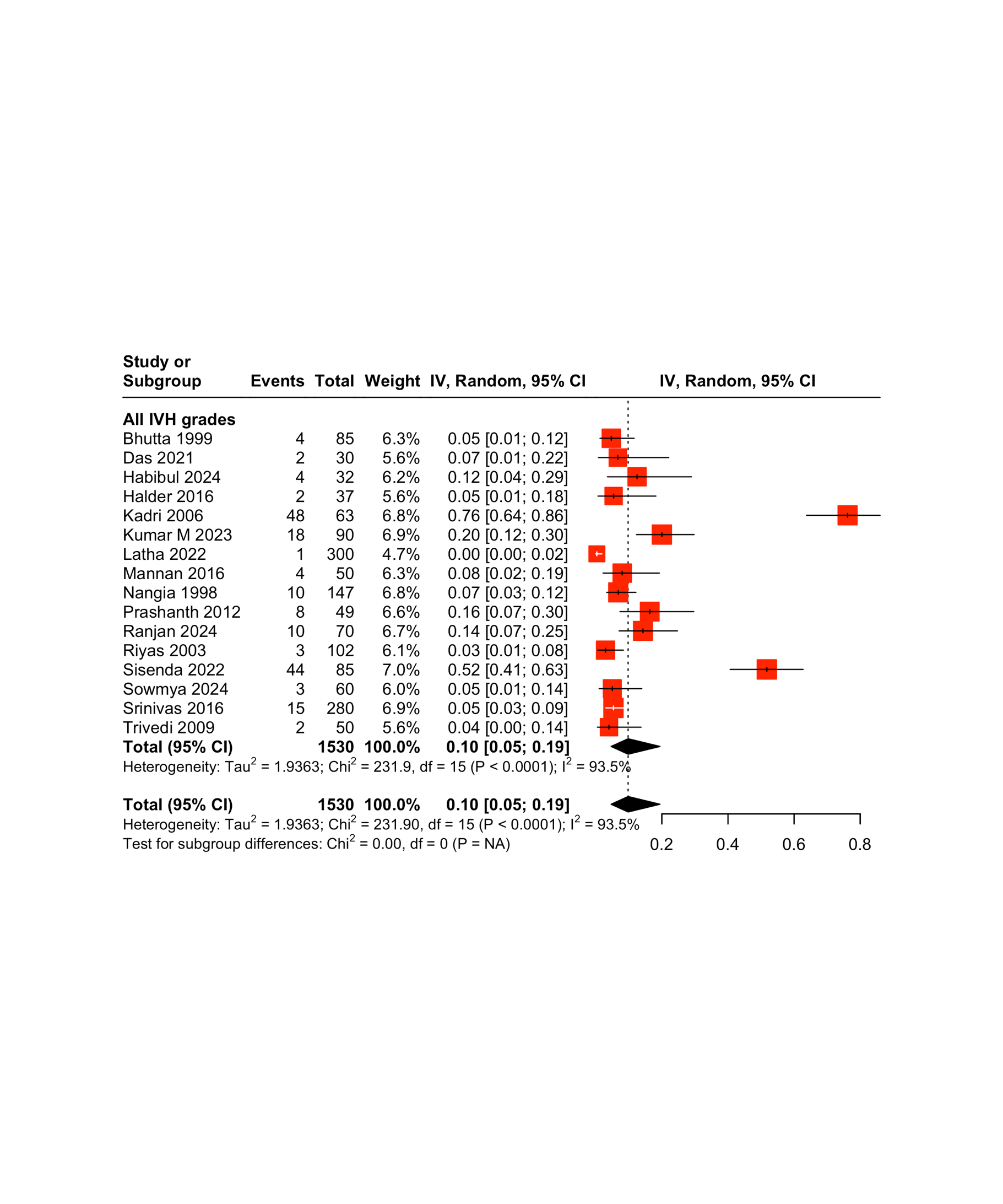


Figure S2: Pooled adjusted odds ratio for any garde intraventricular haemorrhage (IVH) among ventilated neonates.


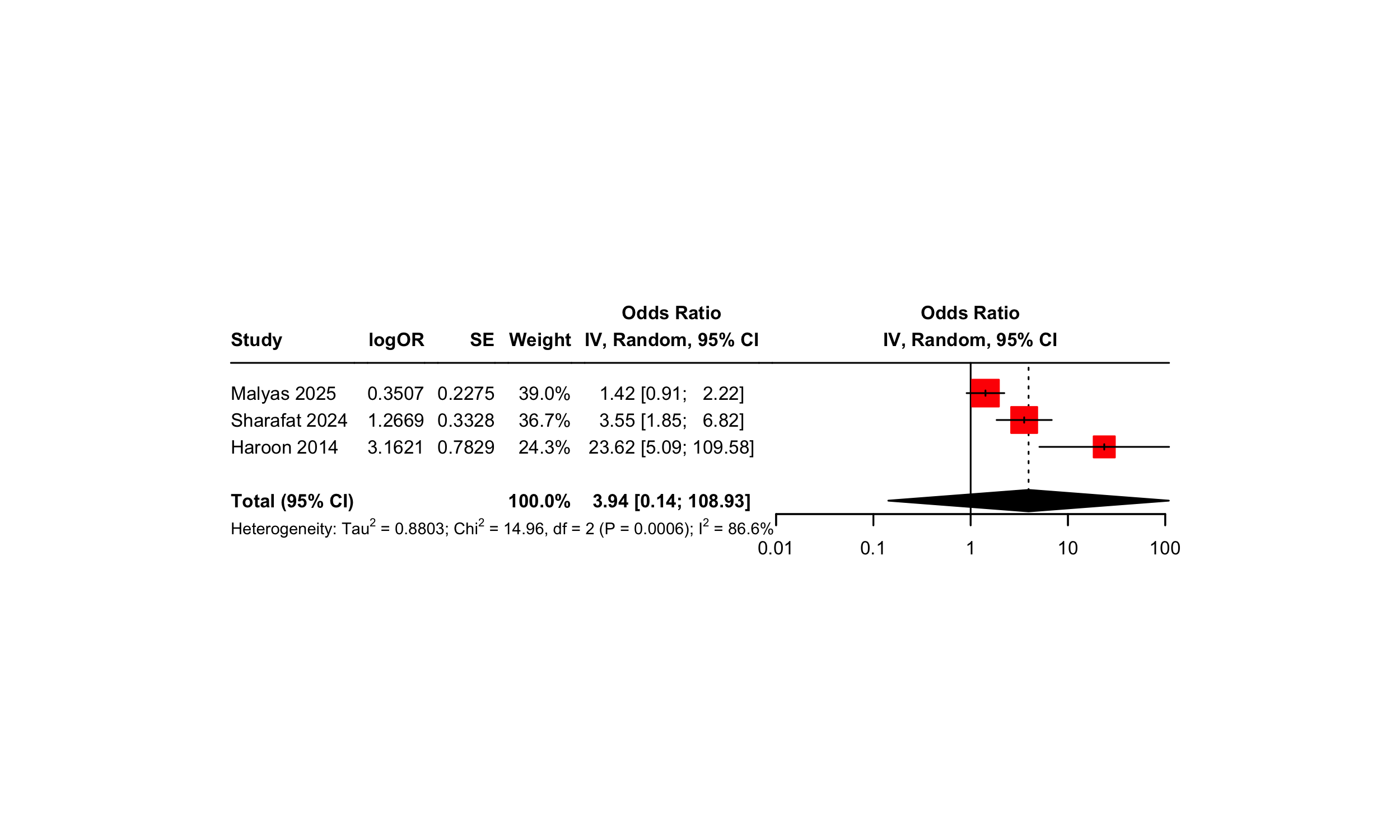


Figure S3: Pooled proportion of necrotising enterocolitis (NEC) among ventilated neonates, as per grade.


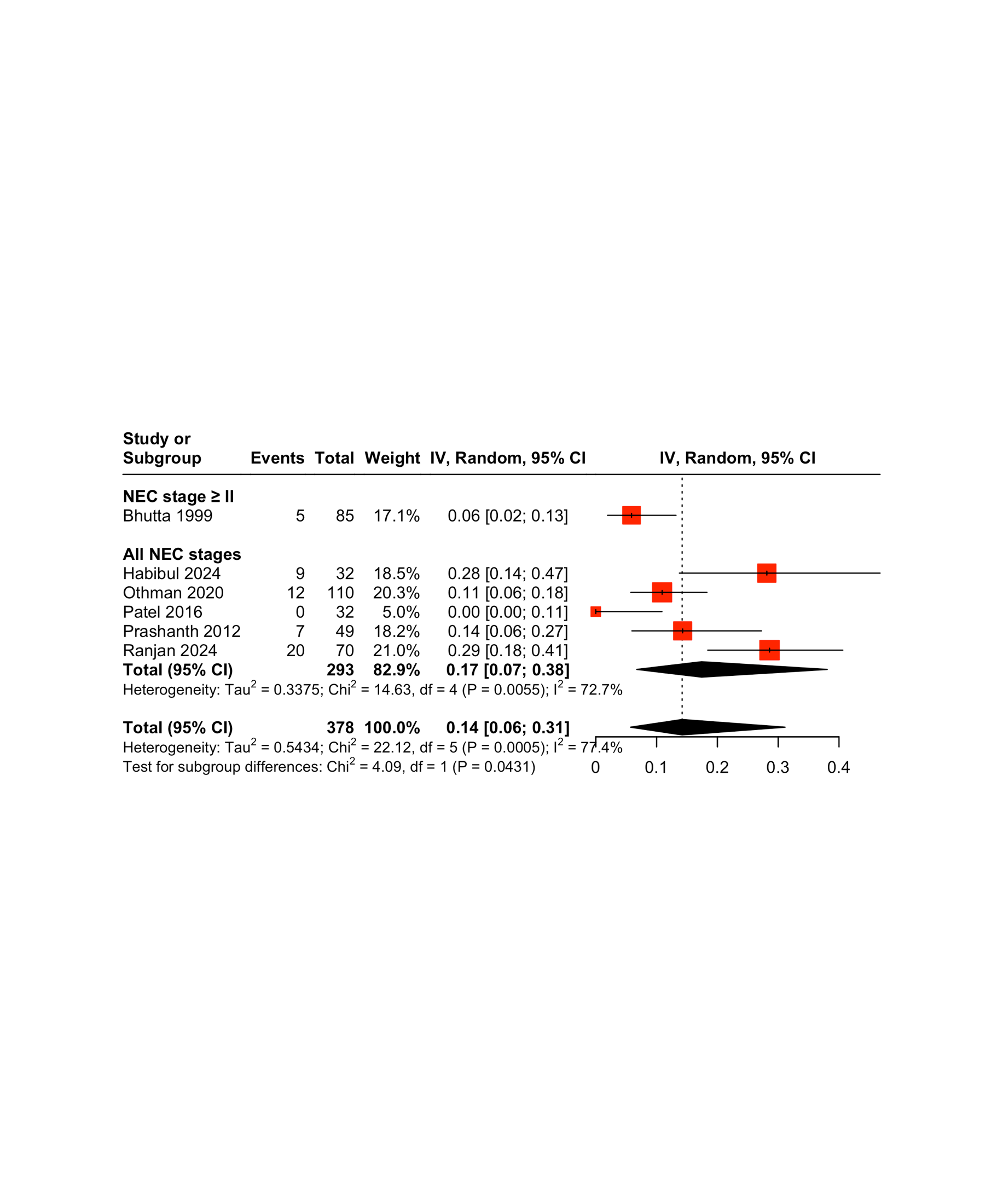


Figure S4: Pooled proportion of retinopathy of prematurity (ROP) among ventilated neonates


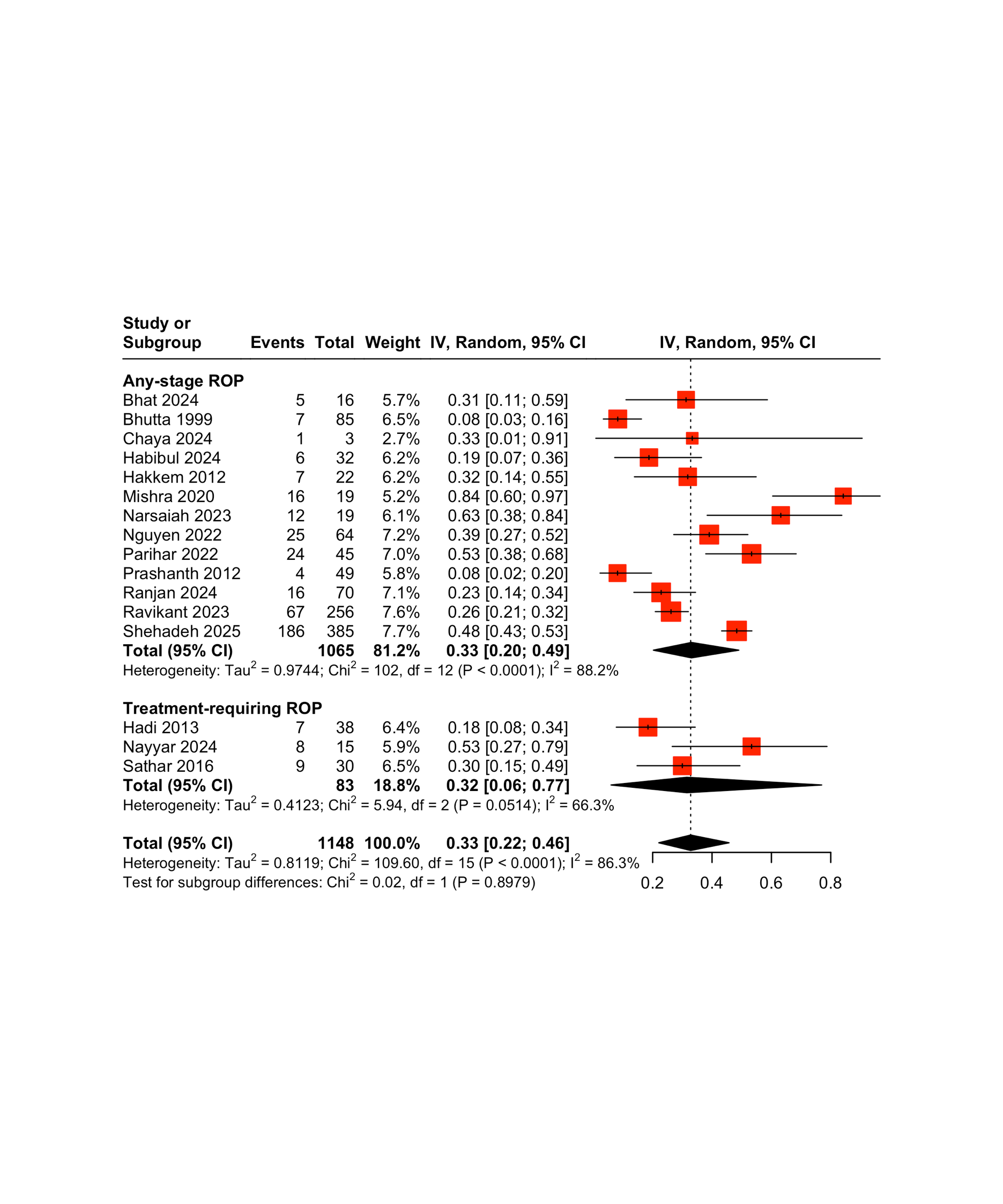


Figure S5: Pooled proportion of ventilator associated pneumonia (VAP) among ventilated neonates.


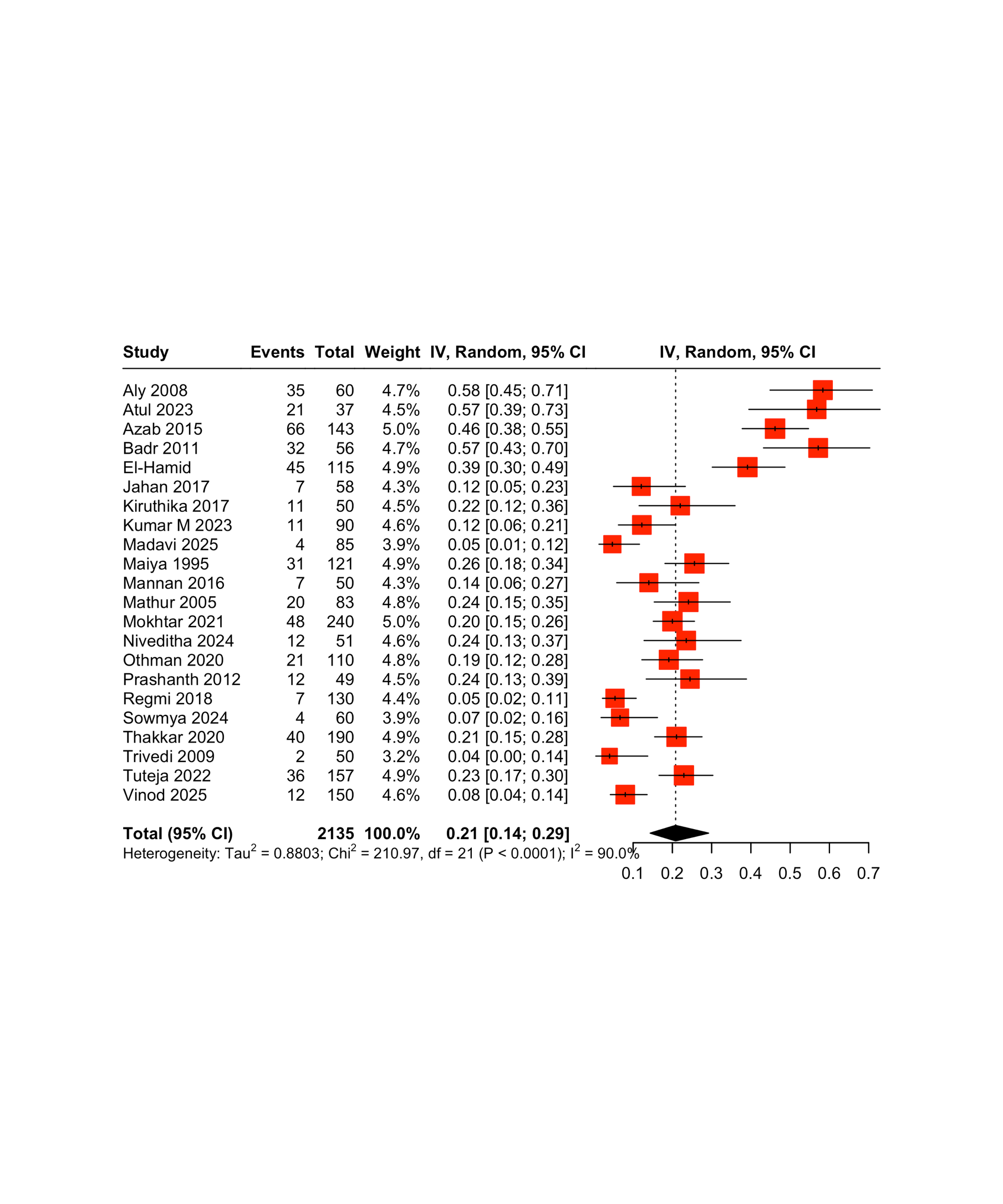


Figure S6: Pooled proportion of sepsis among ventilated neonates.


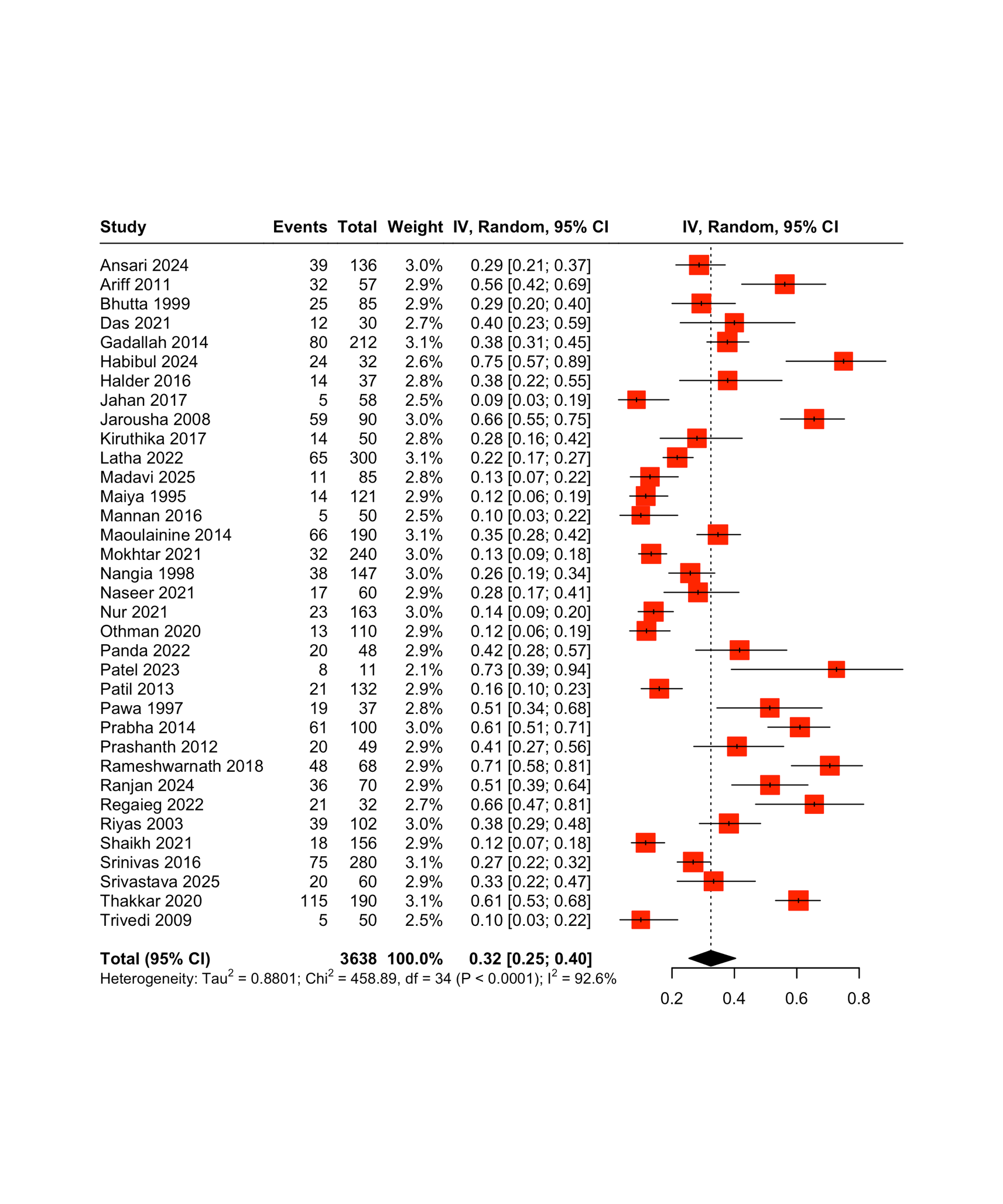


Figure S7: Pooled odds ratio for sepsis among ventilated neonates.


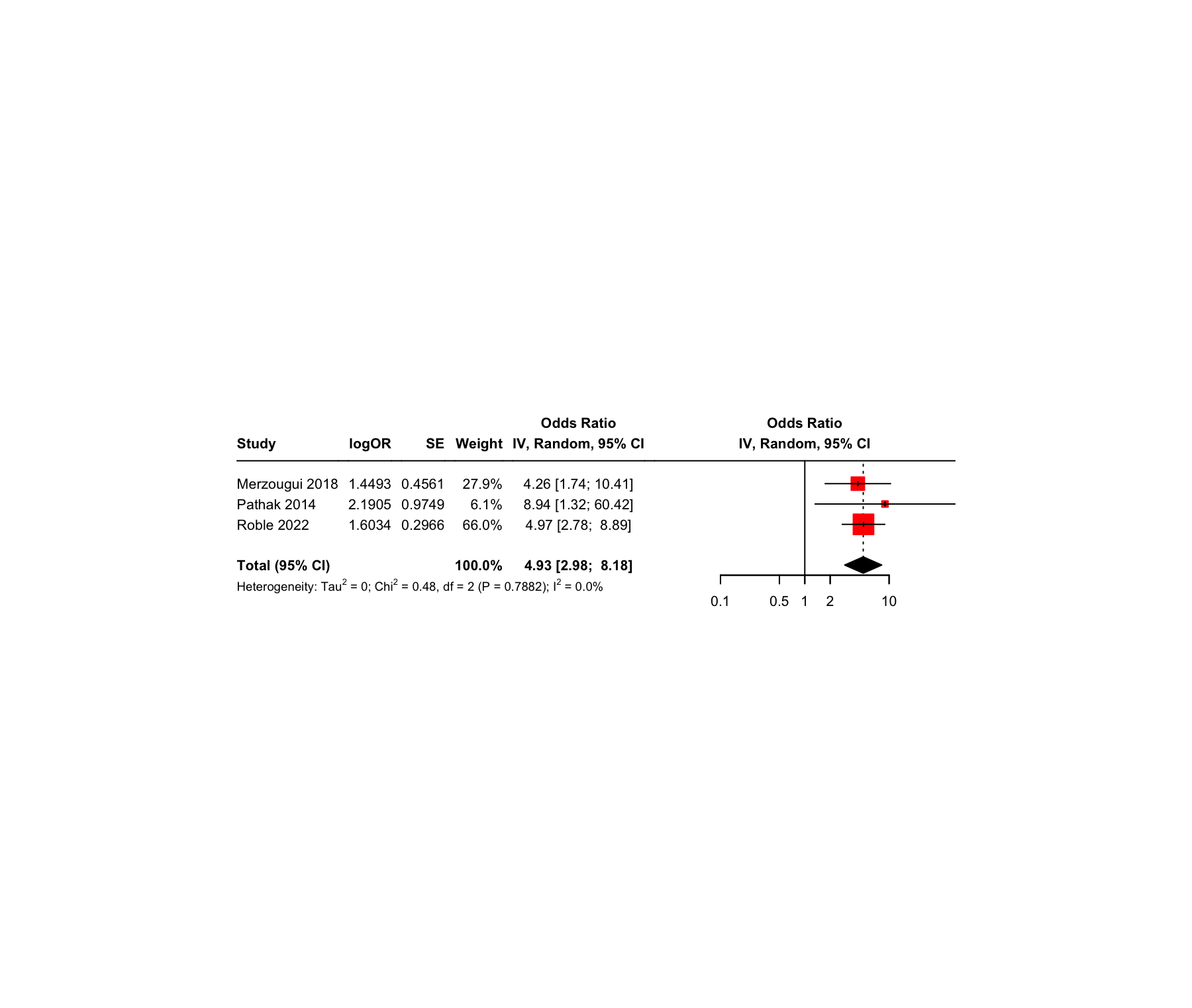


Figure S8: Pooled proportion of pulmonary haemorrhage among ventilated neonates.


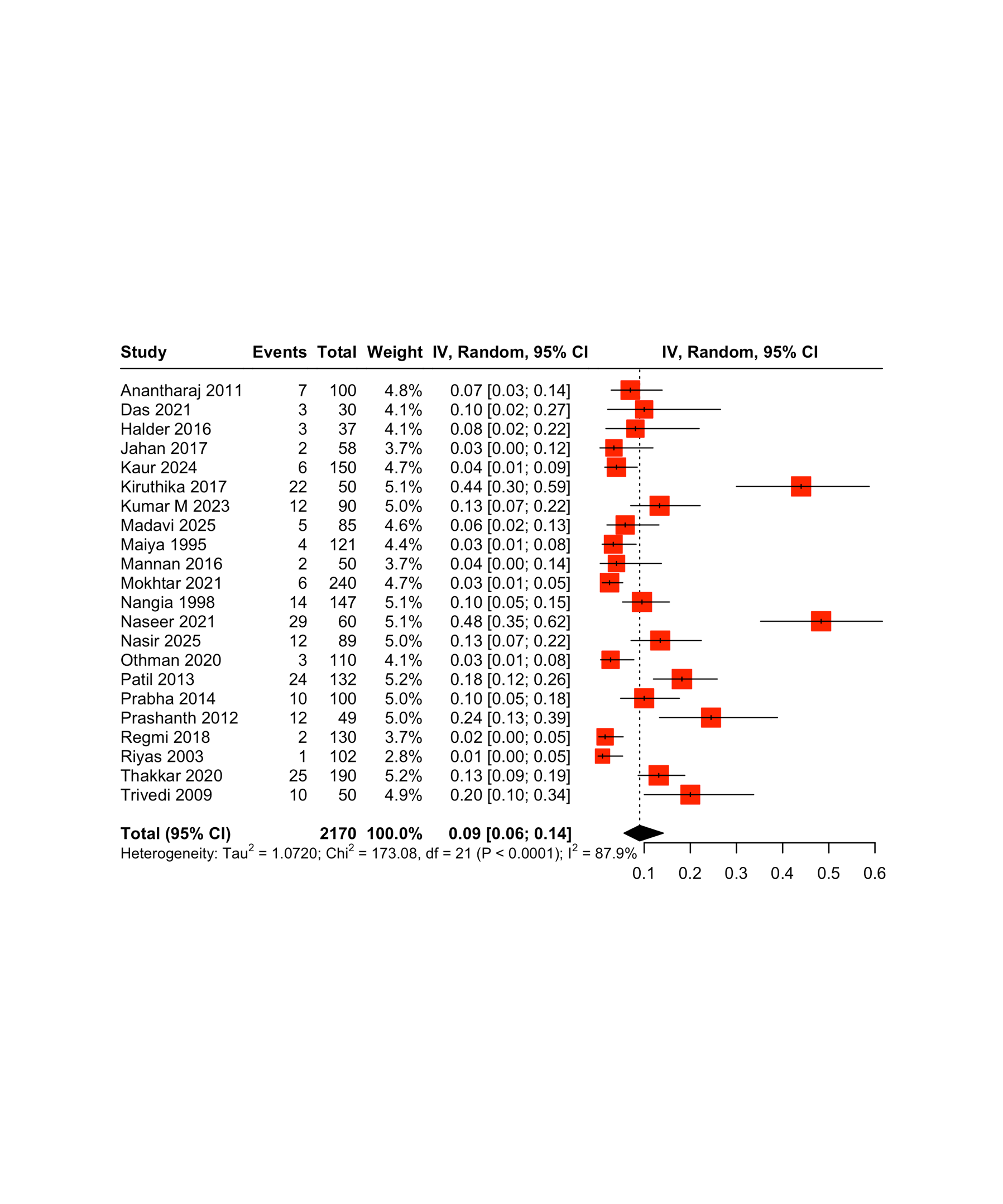


Figure S9: Subgroup analysis for pooled proportion of mortality before discharge among ventilated neonates, based on country of origin.


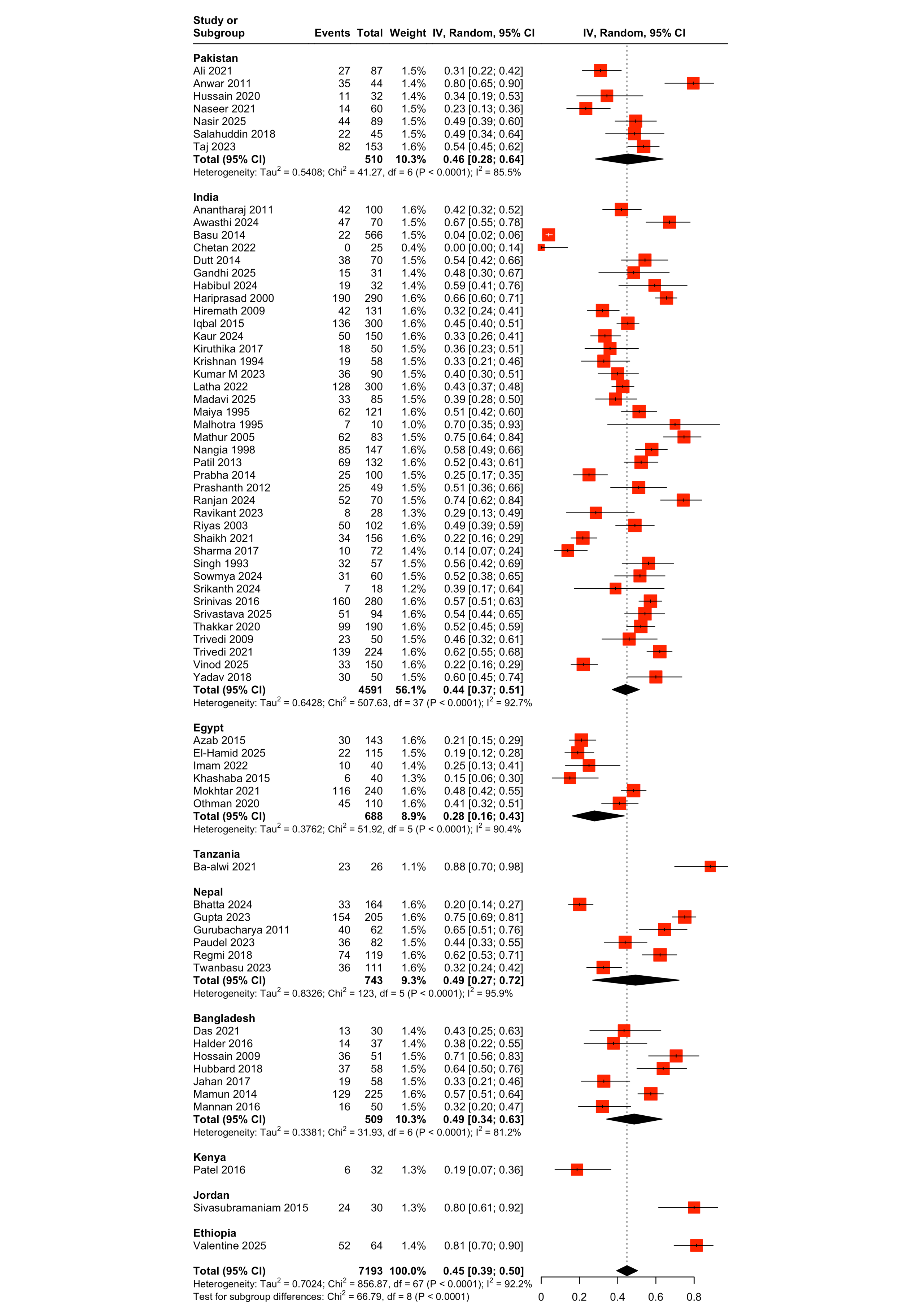


Figure S10: Subgroup analysis for pooled proportion of mortality before discharge among ventilated neonates, based on geographic area.


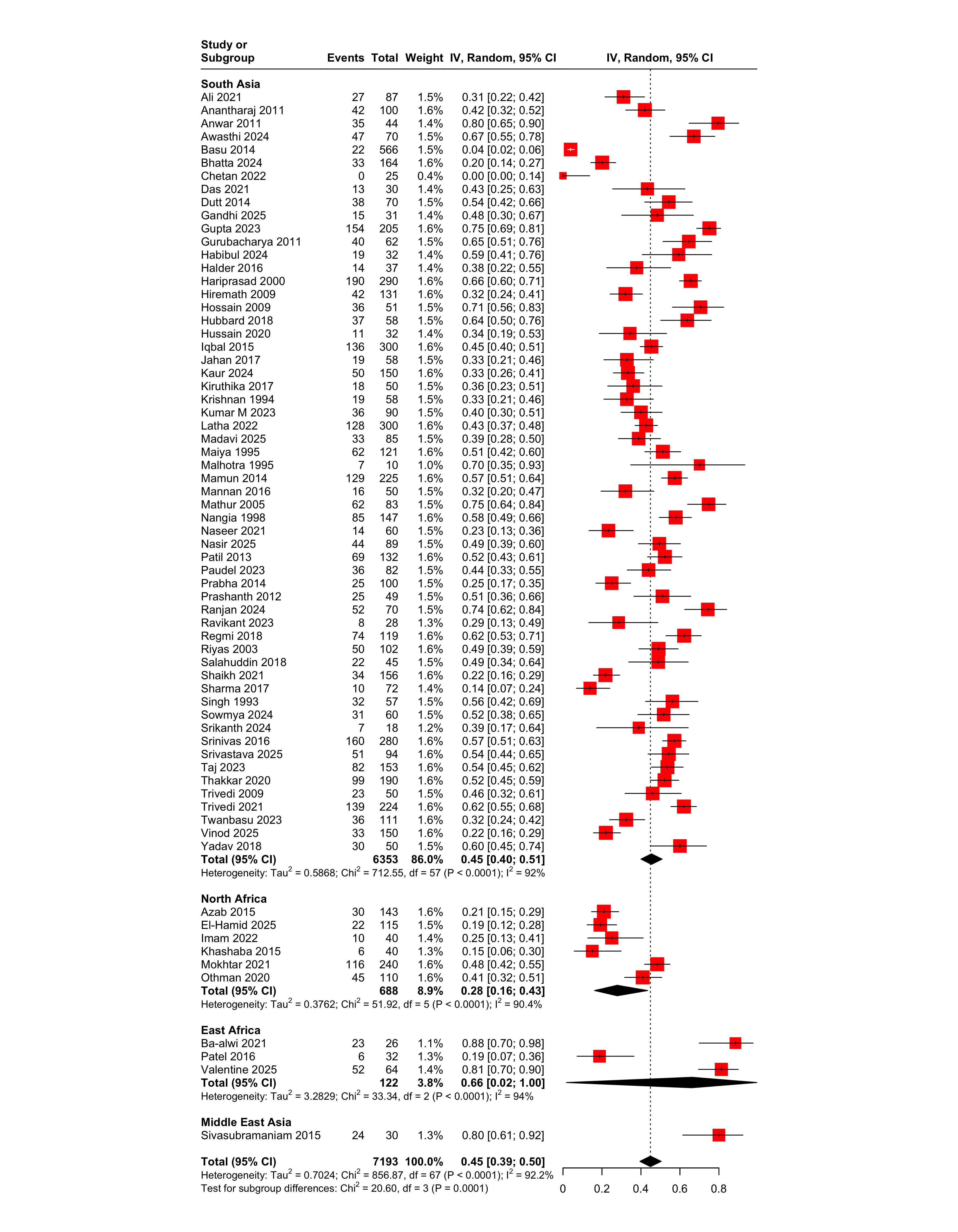


Figure S11: Subgroup analysis for pooled proportion of mortality before discharge among ventilated neonates, based on gestational age.


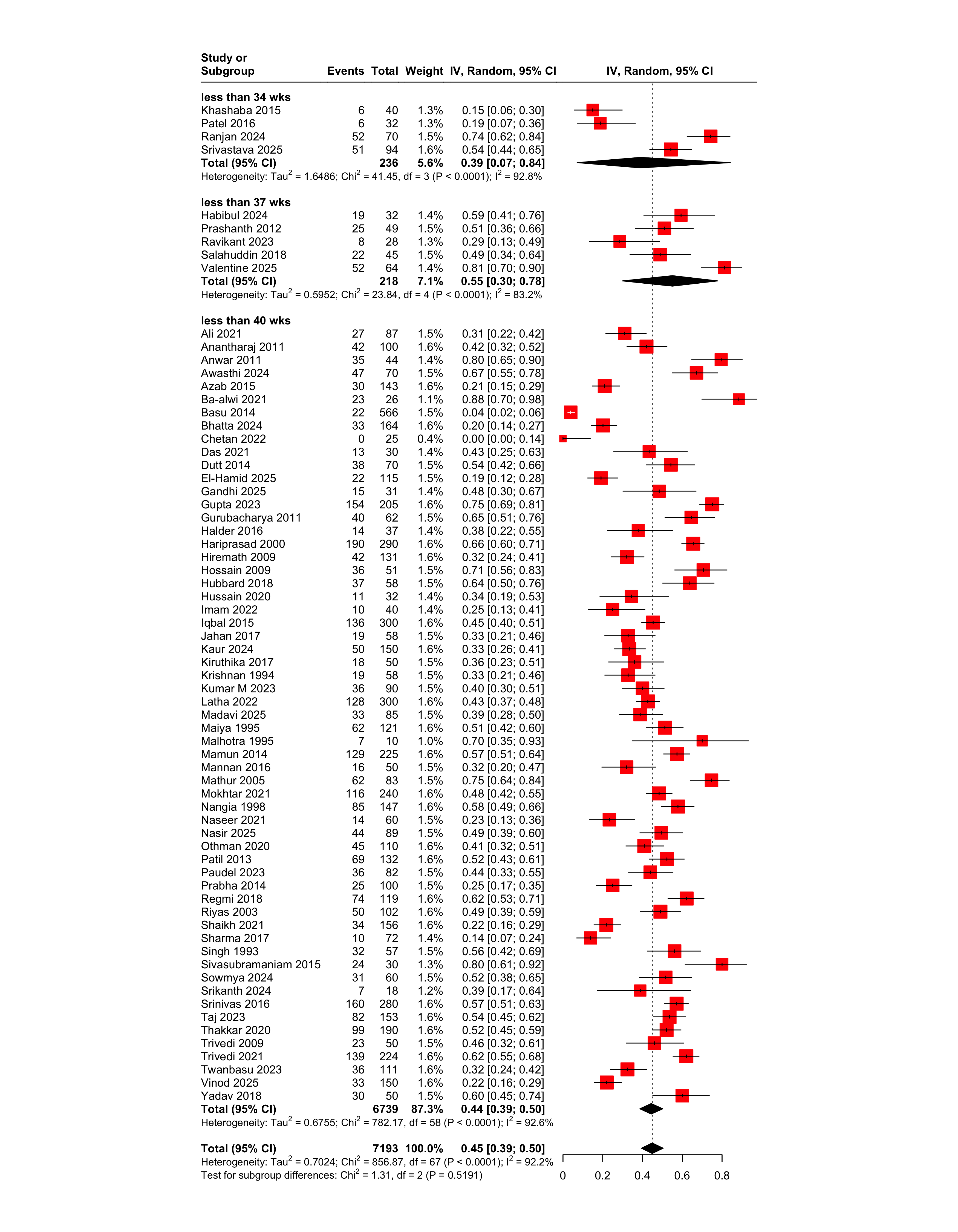


Figure S12: Subgroup analysis for pooled proportion of mortality before discharge among ventilated neonates, based on aetiology.


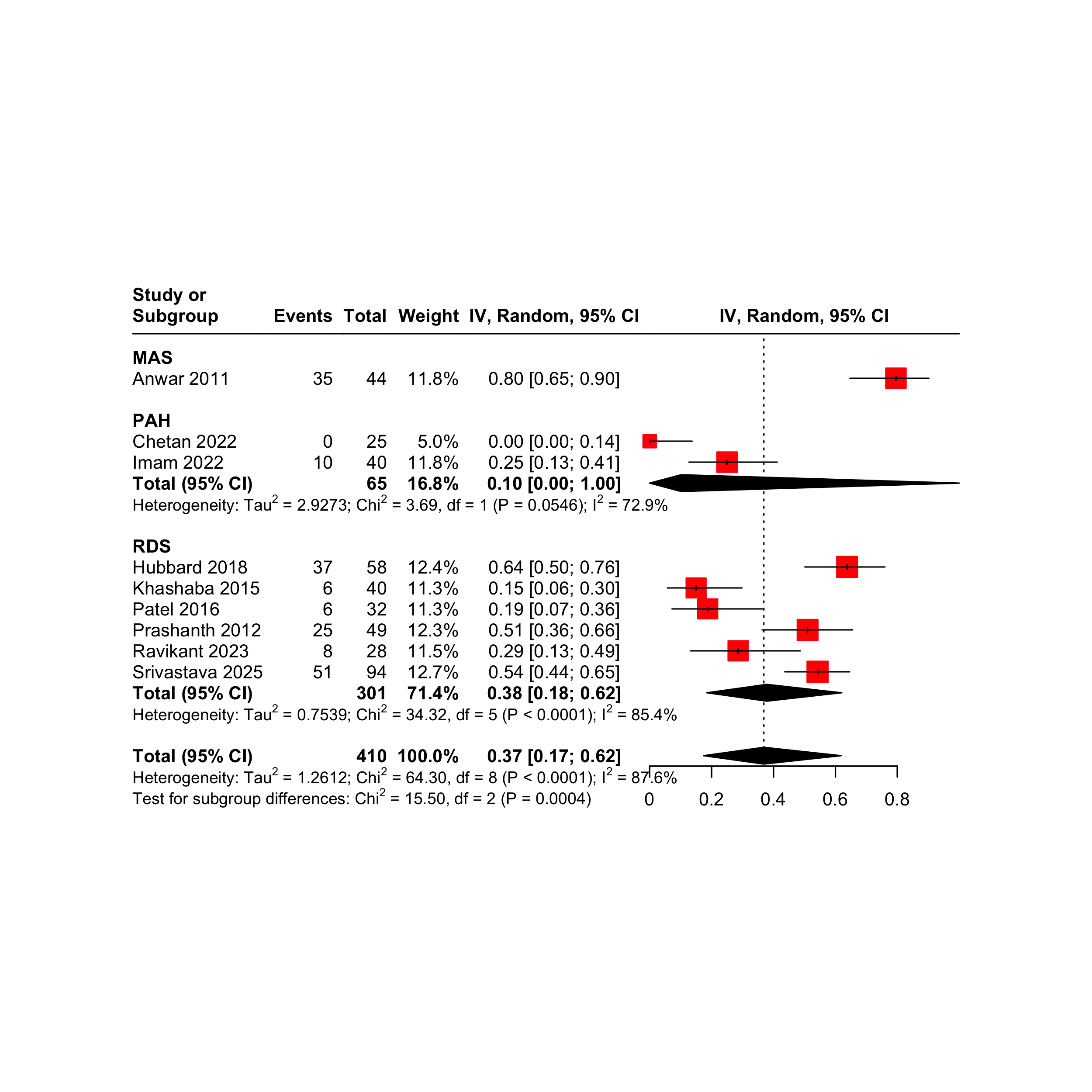


Figure S13: Sensitivity analysis for pooled proportions of mortality, after exclusion of studies with small sample size


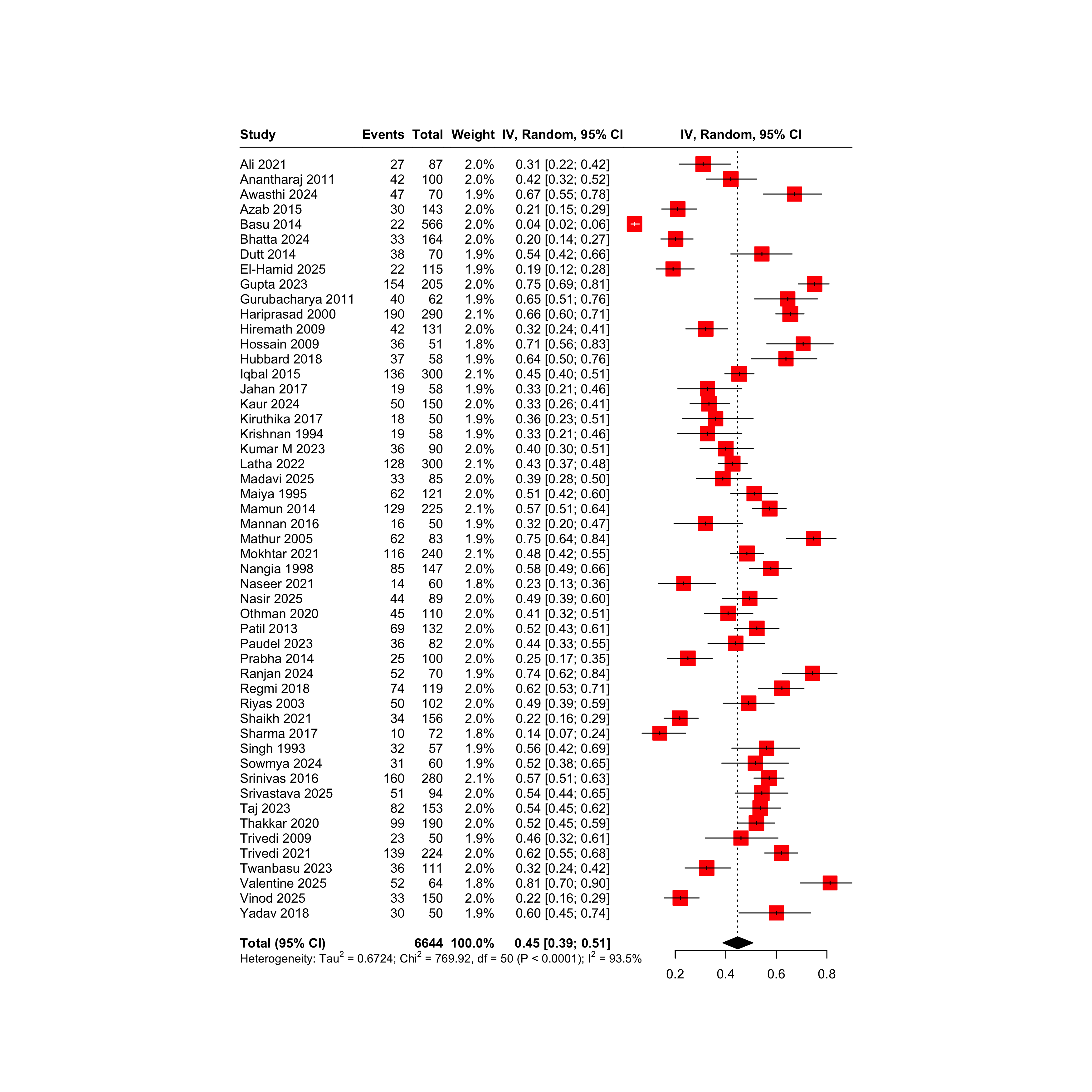


Figure S14: Sensitivity analysis for pooled proportions of mortality, after exclusion of studies with high risk of bias


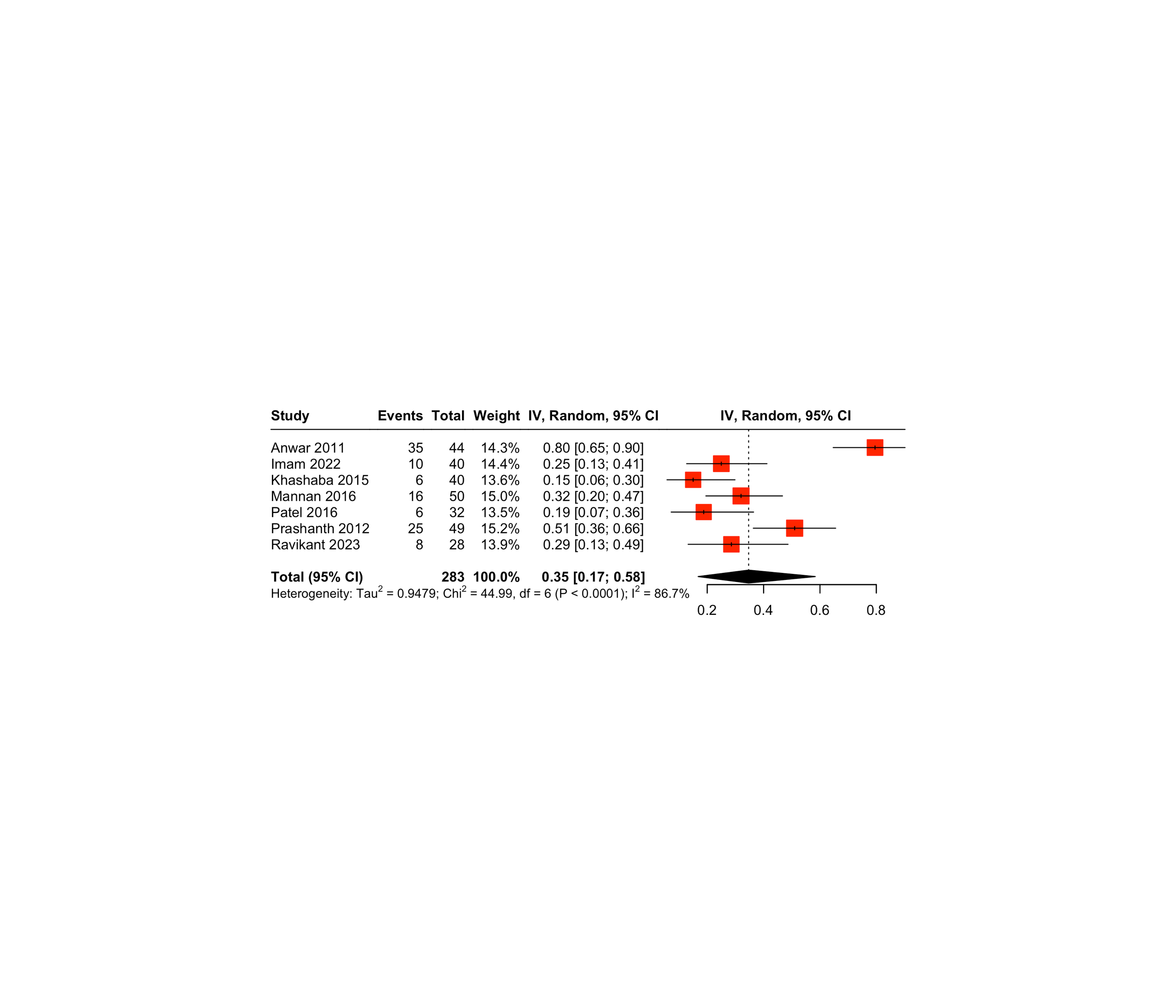


Figure S15: Sensitivity analysis for pooled proportions of mortality, for two epoch (Before 2010 vs After 2010)


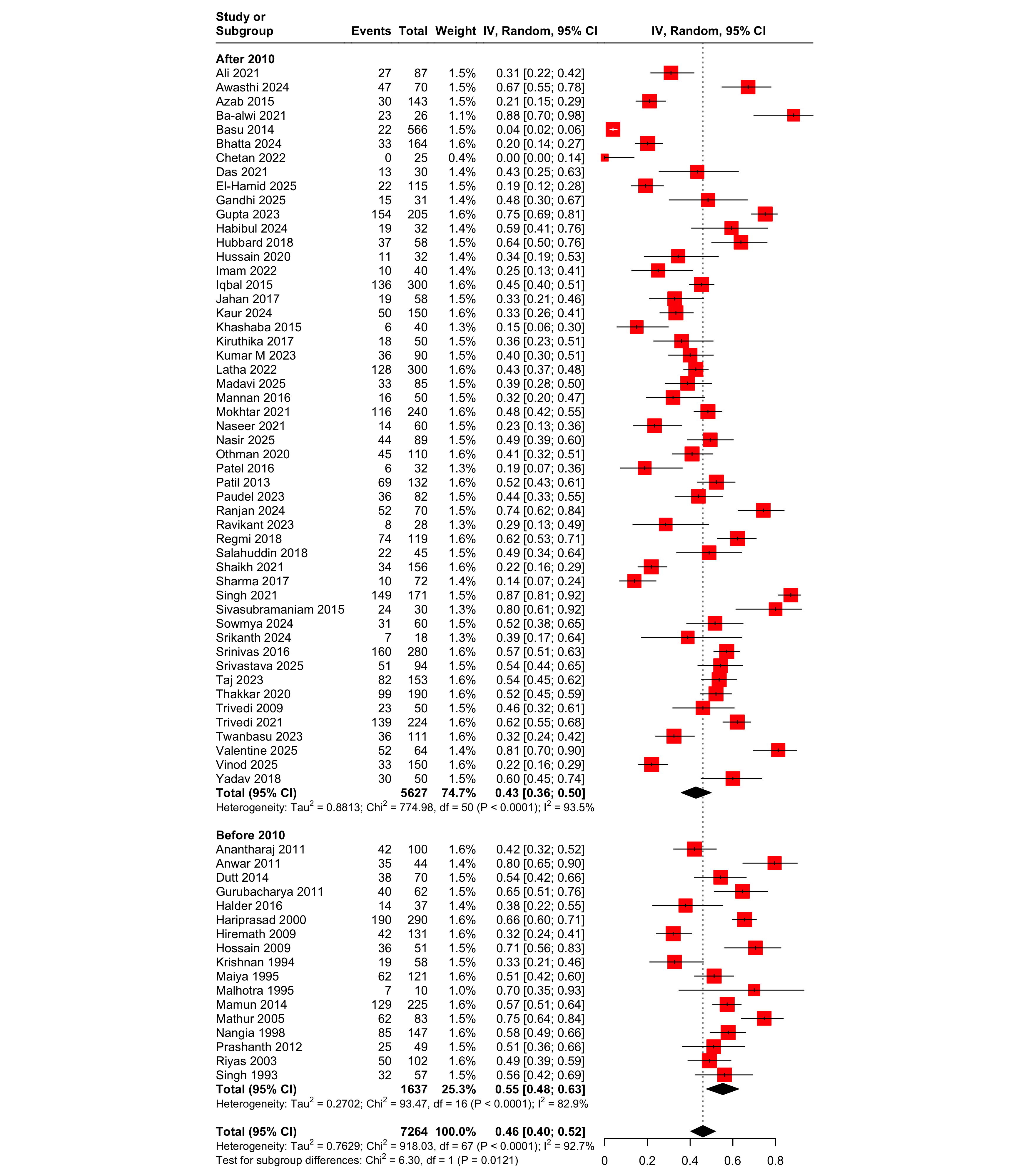


Figure S16: Publication bias for in-hospital mortality before discharge among ventilated neonates


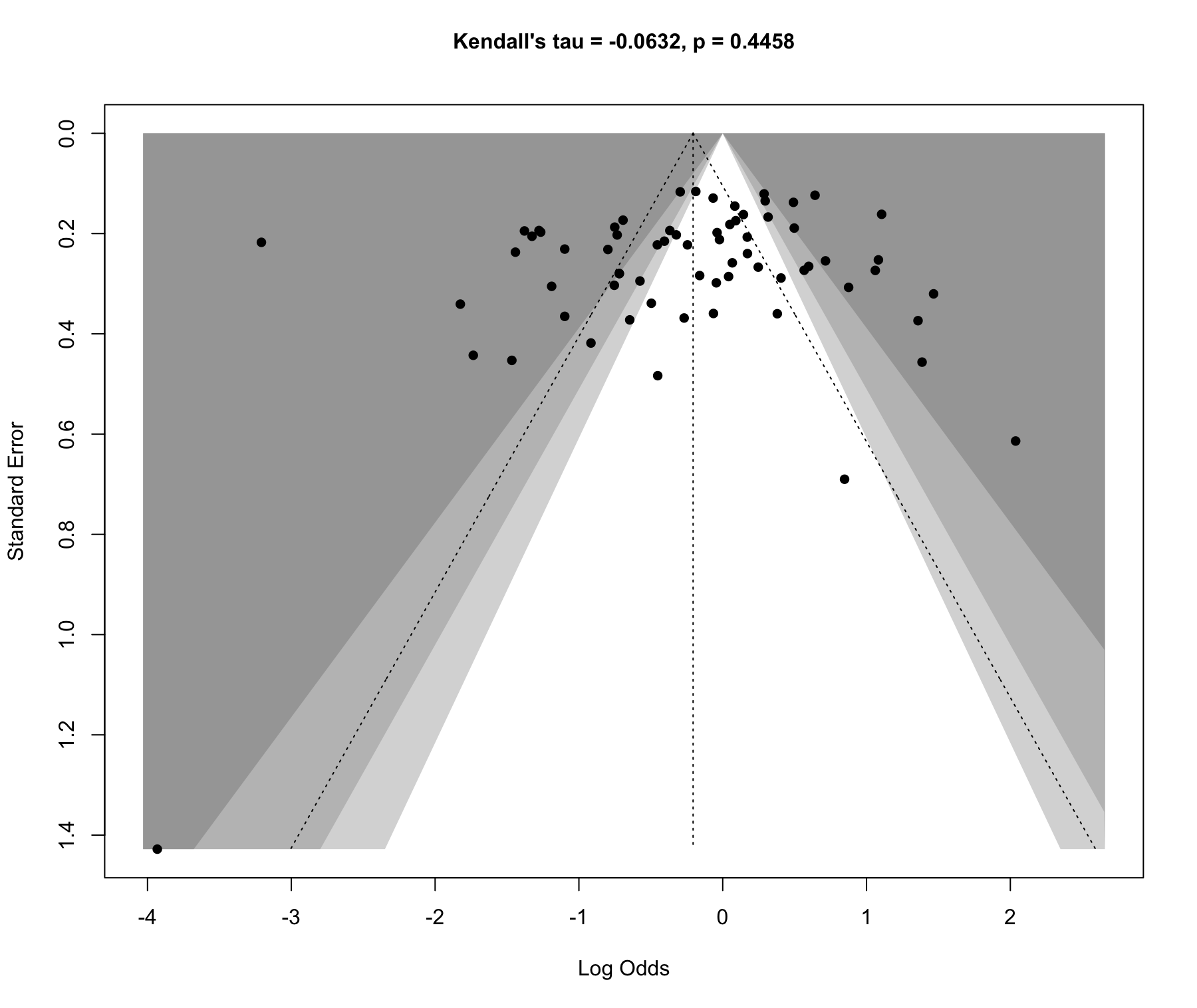


Figure S17: Publication bias for bronchopulmonary dysplasia (BPD) among ventilated neonates


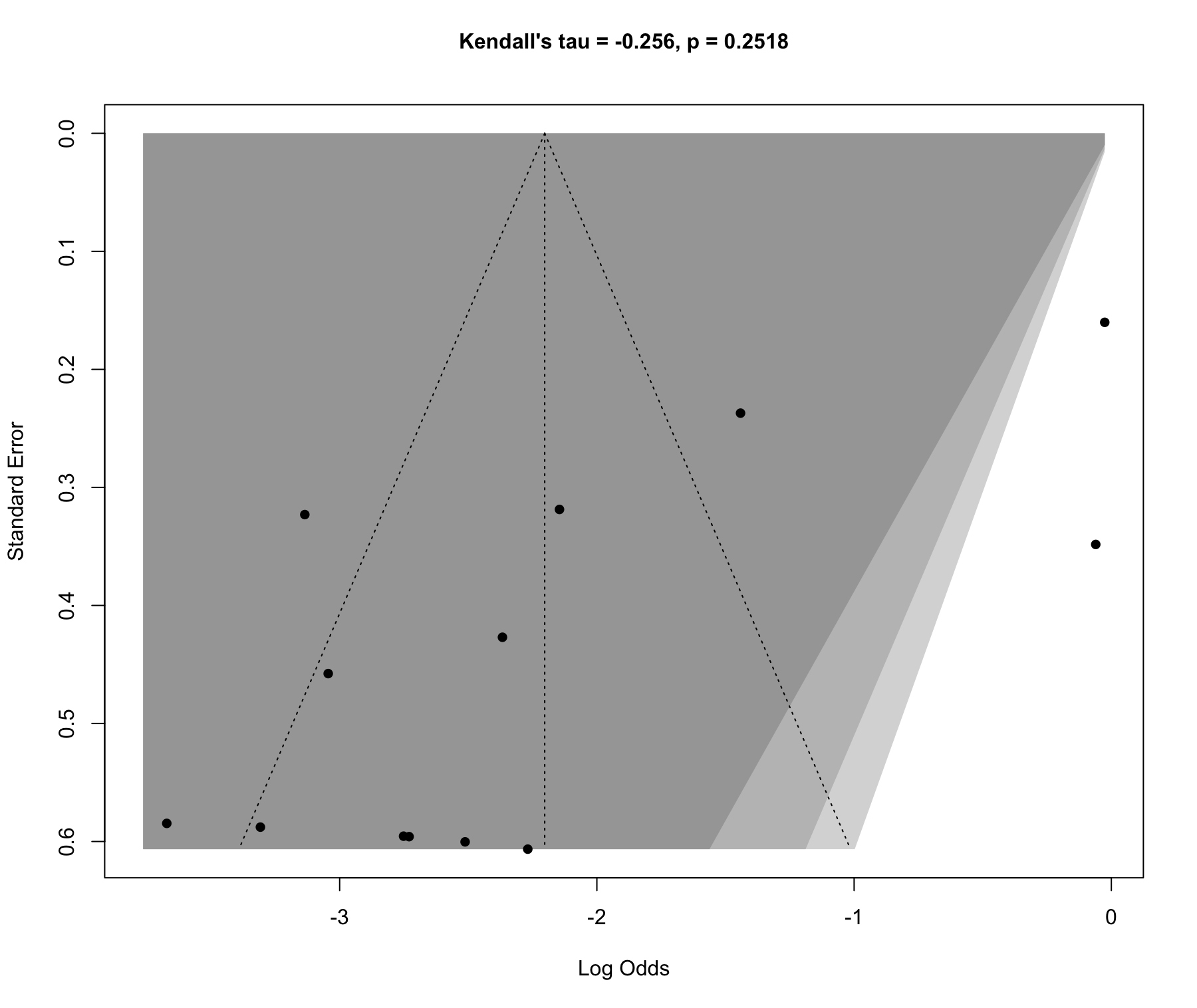


Figure S18: Publication bias for intraventricular haemorrhage (IVH) among ventilated neonates.


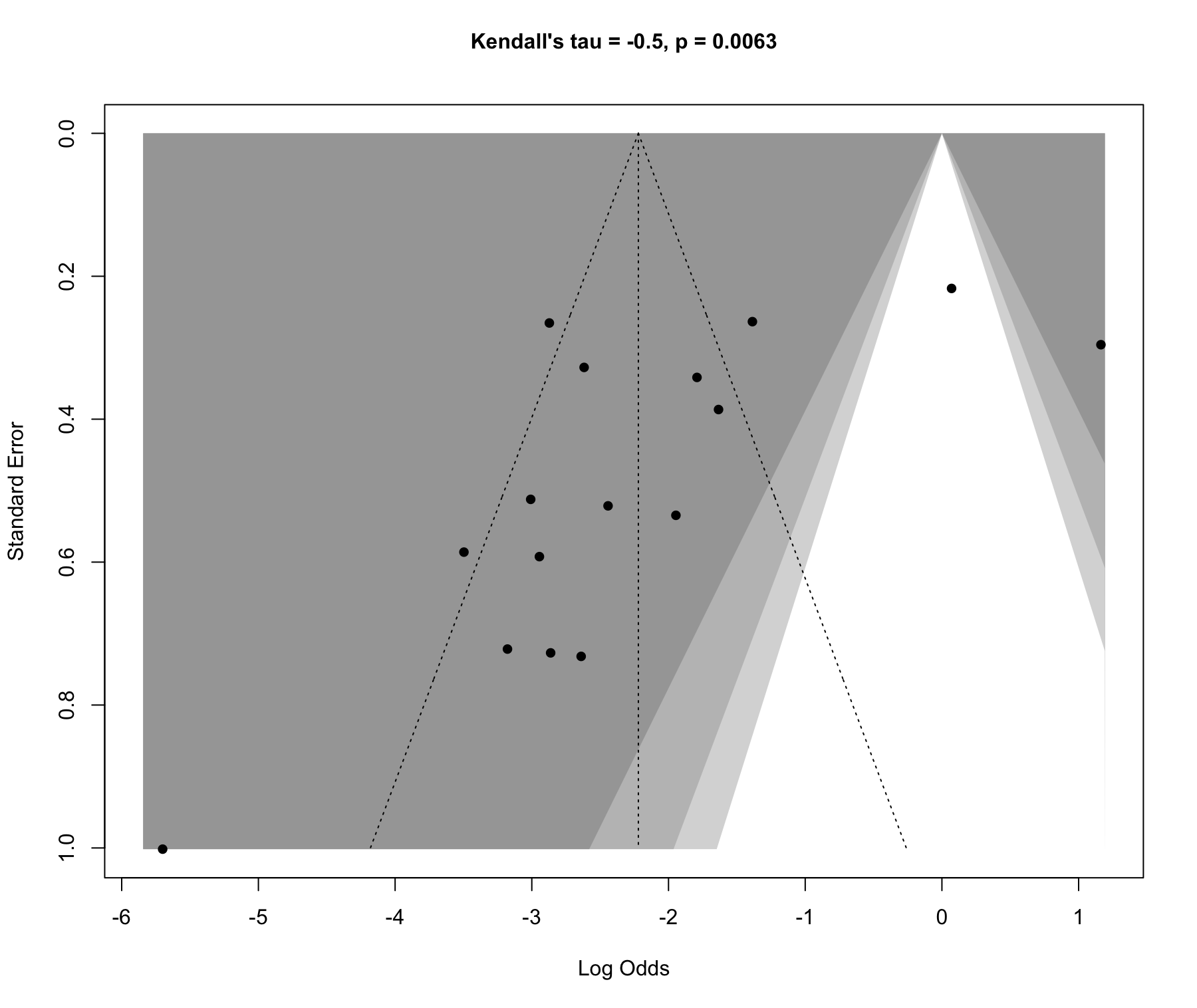


Figure S19: Publication bias for necrotising enterocolitis (NEC) among ventilated neonates


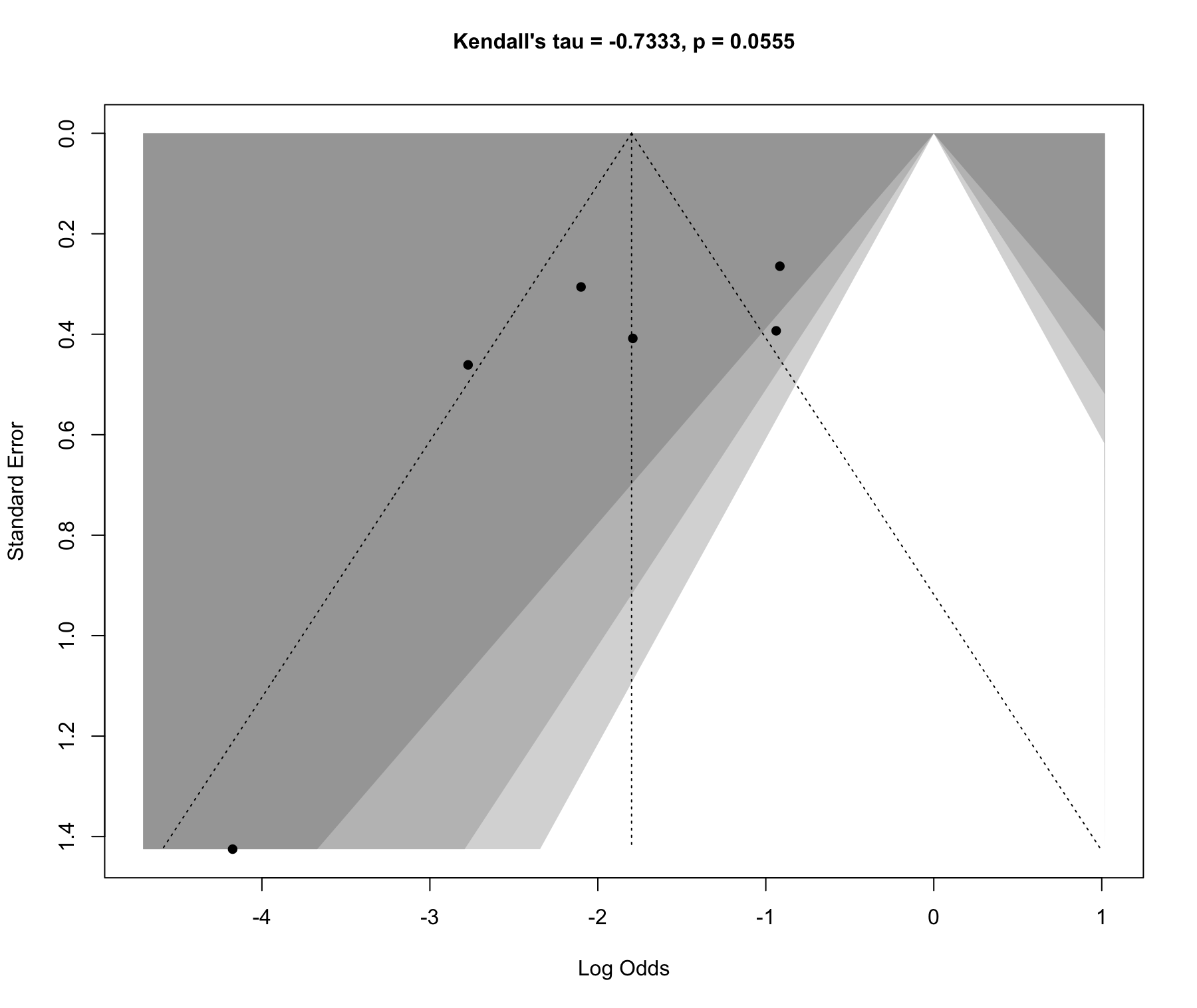


Figure S20: Publication bias for retinopathy of prematurity (ROP) among ventilated neonates


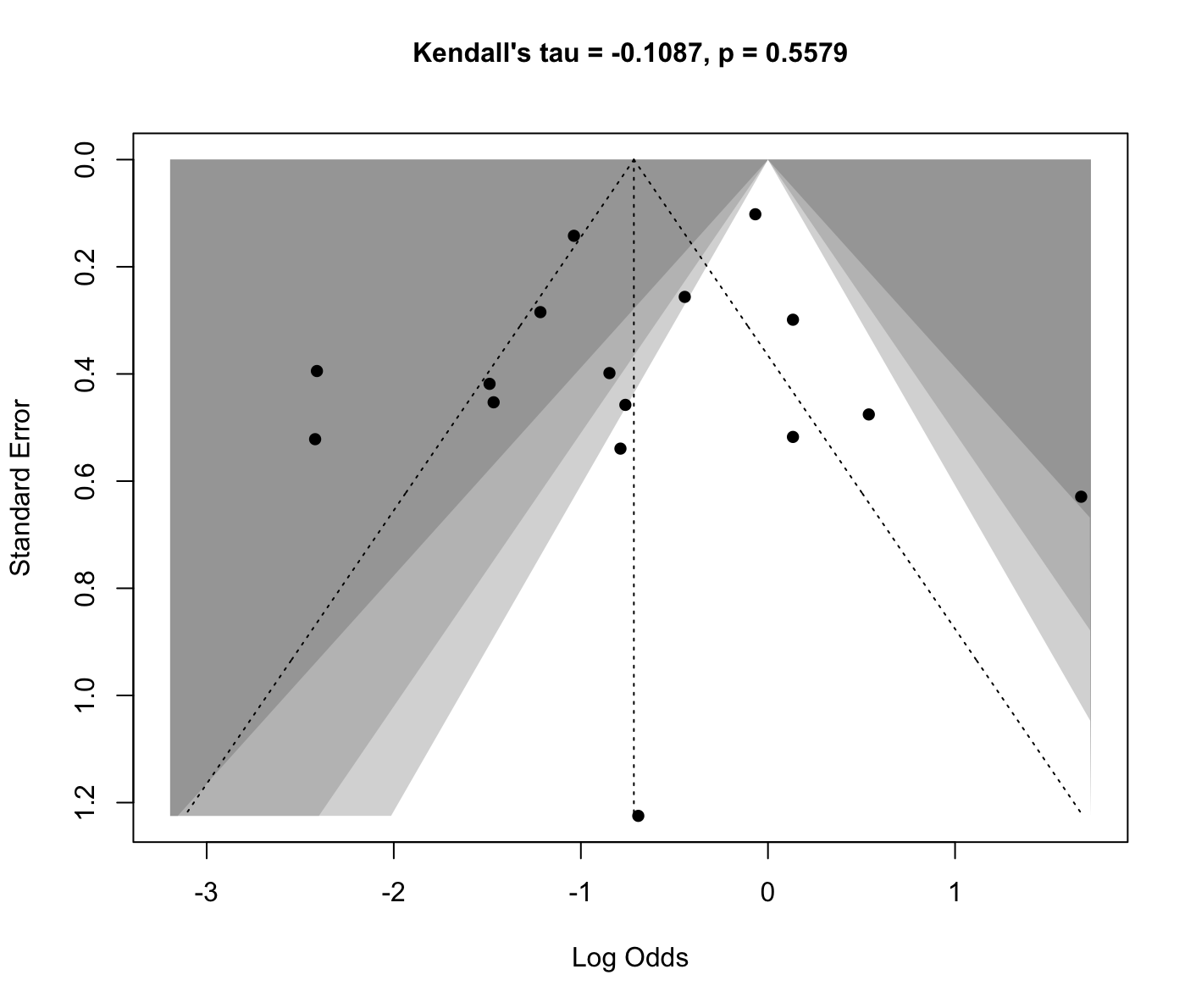


Figure S21: Publication bias for ventilator associated pneumonia (VAP) among ventilated neonates


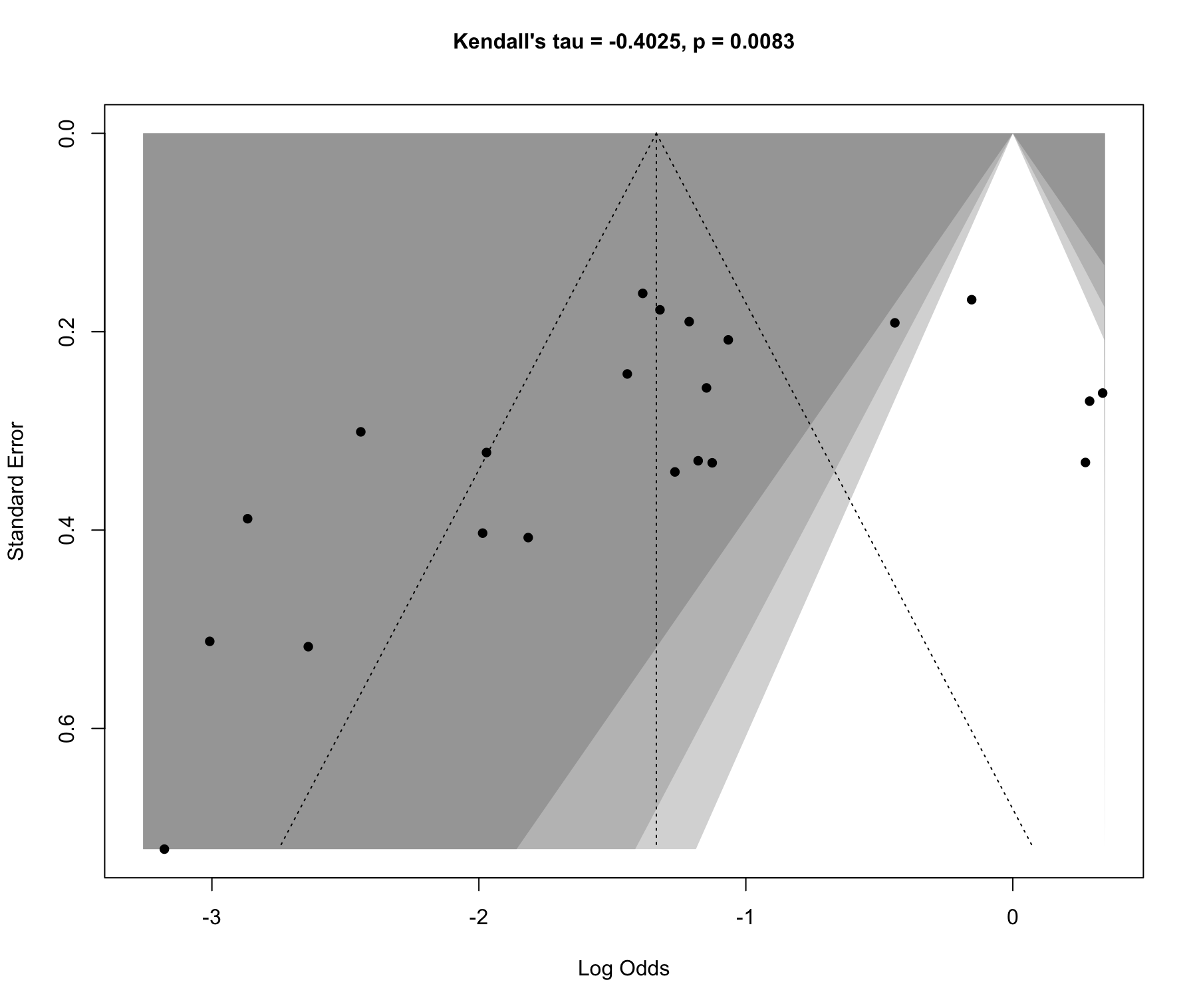


Figure S22: Publication bias for sepsis among ventilated neonates


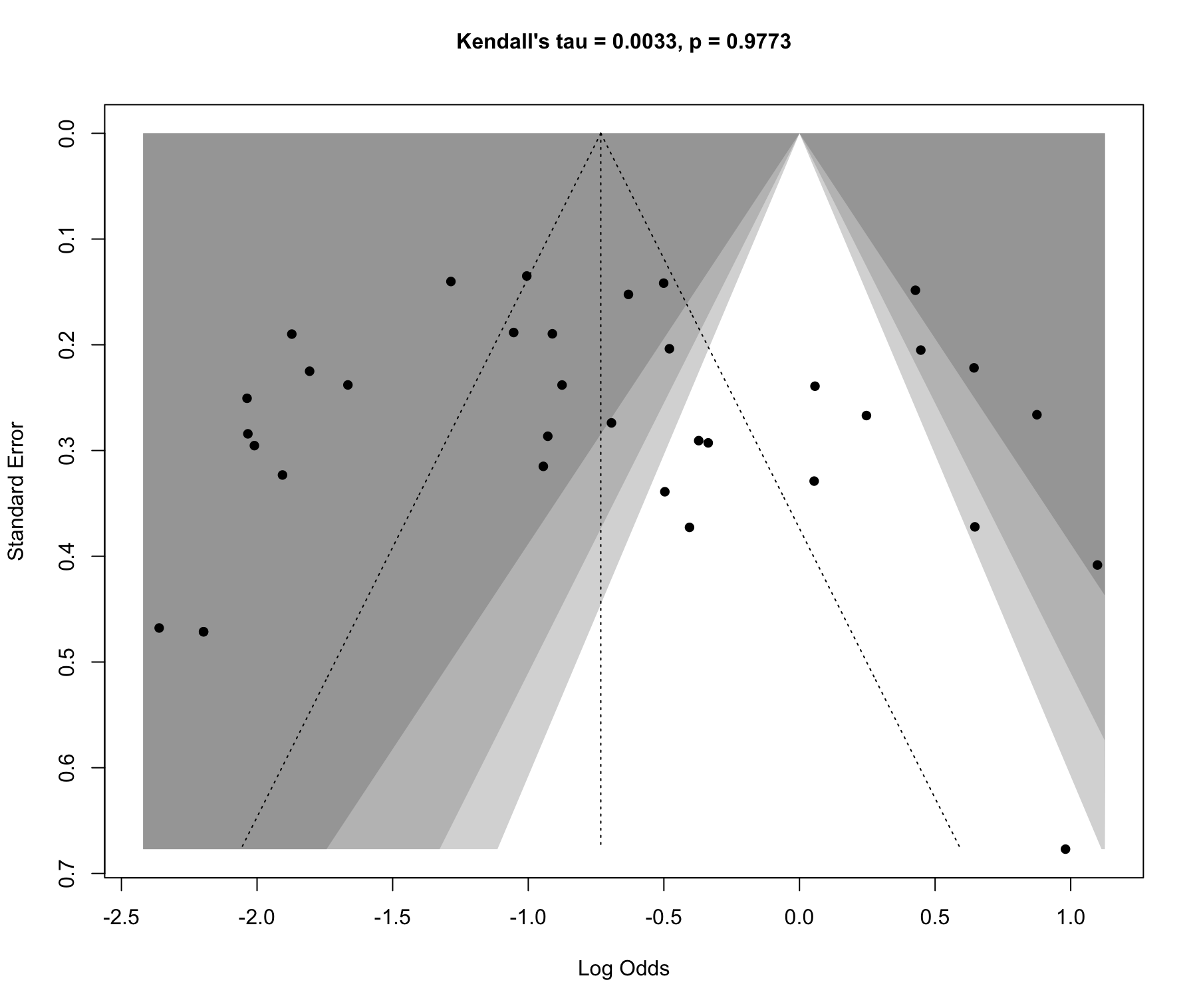


Figure S23: Publication bias for pulmonary haemorrhage among ventilated neonates


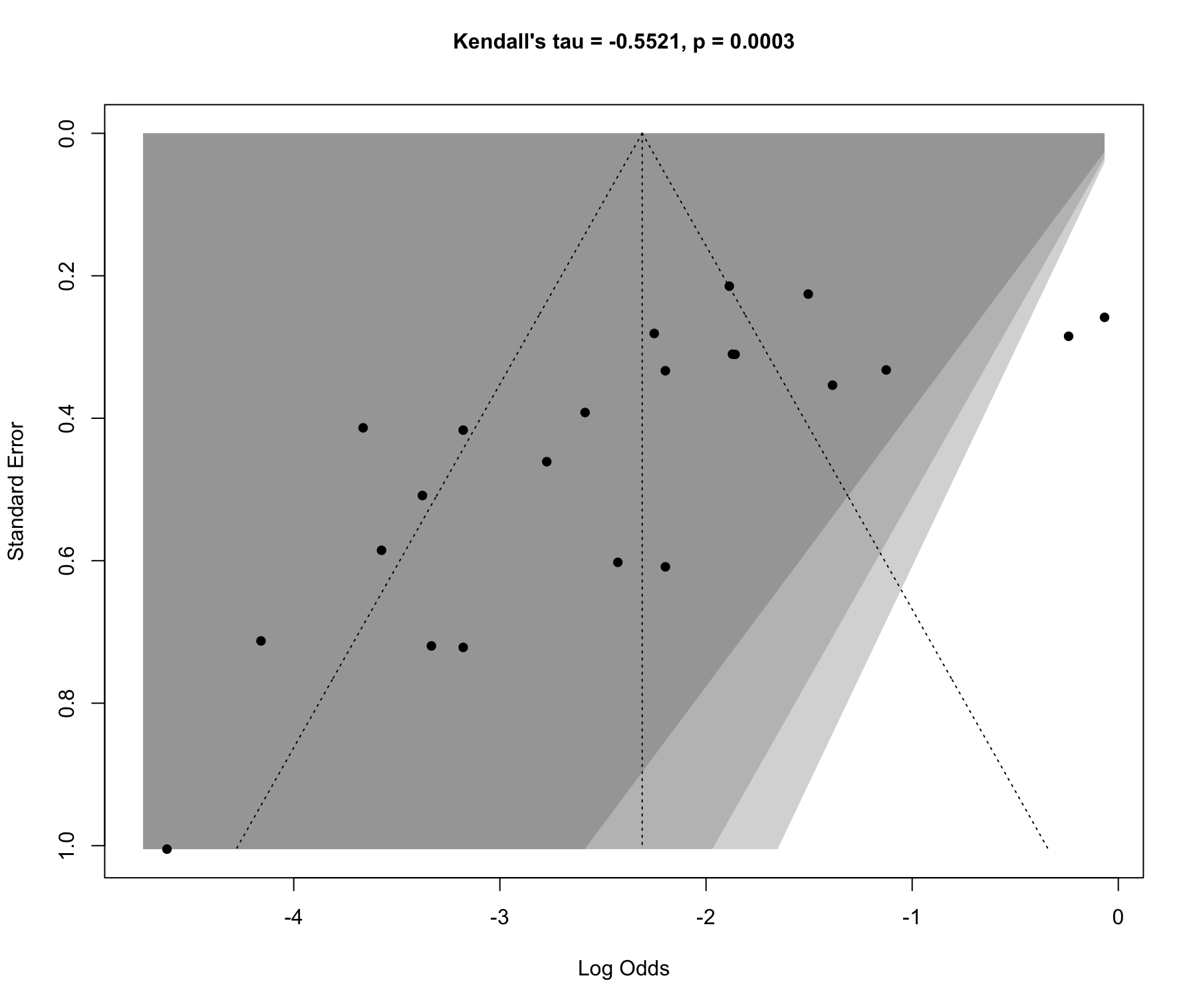


**Table S1:** Characteristics of included studies

| **Author/ year** | **Study Period** | **Study Design** | **Region** | **Country** | **Income level** | **GA/ BW inclusion criteria** | **Ventilated Neonates (n)** |
| --- | --- | --- | --- | --- | --- | --- | --- |
| Malhotra 1995 | 1992-1993 | Prospective cohort | South Asia | India | LMIC | < 40 wks | 10 |
| Pawa 1997 | NS | Prospective cohort | South Asia | India | LMIC | < 40 wks | 37 |
| Nangia 1998 | 1995 | Prospective cohort | South Asia | India | LMIC | < 40 wks | 147 |
| Riyas 2003 | 2000-2002 | Prospective cohort | South Asia | India | LMIC | < 40 wks | 102 |
| Aly 2008 | 2005 | Prospective cohort | North Africa | Egypt | LMIC | < 40 wks | 60 |
| Hiremath 2009 | NS | Prospective cohort | South Asia | India | LMIC | < 40 wks | 131 |
| Hossain 2009 | 2006 | Prospective cohort | South Asia | Bangladesh | LMIC | < 40 wks | 51 |
| Trivedi 2009 | 2007-2008 | Prospective cohort | South Asia | India | LMIC | < 40 wks | 50 |
| Anantharaj 2011 | 2007-2009 | Prospective cohort | South Asia | India | LMIC | < 40 wks | 100 |
| Anwar 2011 | 2008-2009 | Prospective cohort | South Asia | Pakistan | LMIC | < 40 wks | 44 |
| Gurubacharya 2011 | 2009 | Prospective cohort | South Asia | Nepal | LMIC | < 40 wks | 62 |
| Hakkem 2012 | 2009-2010 | Prospective cohort | North Africa | Egypt | LMIC | < 34 wks/ < 2000 gm | 22 |
| Prashanth 2012 | 2007-2008 | Prospective cohort | South Asia | India | LMIC | 26-36 wks | 49 |
| Zakariya 2012 | 2004-2006 | Prospective cohort | South Asia | India | LMIC | < 40 wks | NS |
| Hadi 2013 | 2010-2012 | Prospective cohort | North Africa | Egypt | LMIC | <32 wks/<1250 gm | 38 |
| Mukhopadhyay 2013 | 2009-2011 | Prospective cohort | South Asia | India | LMIC | <1000 gm | NS |
| Patil 2013 | 2009-2011 | Prospective cohort | South Asia | India | LMIC | < 40 wks | 132 |
| Dutt 2014 | 2009-2010 | Prospective cohort | South Asia | India | LMIC | < 40 wks | 79 |
| Gadallah 2014 | 2012 | Prospective cohort | North Africa | Egypt | LMIC | < 40 wks | 212 |
| Haroon 2014 | 2004-2009 | Prospective cohort | South Asia | Pakistan | LMIC | <35 wks | 62 |
| Mamun 2014 | 2006-2009 | Prospective cohort | South Asia | Bangladesh | LMIC | < 40 wks | 225 |
| Maoulainine 2014 | 2009-2010 | Prospective cohort | North Africa | Morrocco | LMIC | < 40 wks | 190 |
| Pathak 2014 | 2010-2012 | Prospective cohort | South Asia | India | LMIC | < 40 wks | NS |
| Azab 2015 | 2013-2014 | Prospective cohort | North Africa | Egypt | LMIC | < 40 wks | 143 |
| Iqbal 2015 | 2011-2013 | Prospective cohort | South Asia | India | LMIC | < 40 wks | 300 |
| Khashaba 2015 | 2013-2015 | Prospective cohort | North Africa | Egypt | LMIC | < 34 wks | 40 |
| Sivasubramaniam 2015 | 2010-2011 | Prospective cohort | Middle East Asia | Jordan | LMIC | < 40 wks | 30 |
| Halder 2016 | 2009-2010 | Prospective cohort | South Asia | Bangladesh | LMIC | < 40 wks | 37 |
| Sathar 2016 | 2013-2015 | Prospective cohort | South Asia | India | LMIC | <32 wks/< 1500 gm | 30 |
| Bhunwal 2017 | 2012-2013 | Prospective cohort | South Asia | India | LMIC | < 33 wks | 33 |
| Kiruthika 2017 | 2014-2015 | Prospective cohort | South Asia | India | LMIC | < 40 wks | 50 |
| Yadav 2018 | 2013-2014 | Prospective cohort | South Asia | India | LMIC | < 40 wks | 50 |
| Baseer 2020 | 2017-2018 | Prospective cohort | North Africa | Egypt | LMIC | < 40 wks | NS |
| Mishra 2020 | 2017-2019 | Prospective cohort | South Asia | India | LMIC | <34 wks /<2000 gm | 19 |
| Othman 2020 | 2019-2020 | Prospective cohort | North Africa | Egypt | LMIC | < 40 wks | 110 |
| Thakkar 2020 | 2015-2016 | Prospective cohort | South Asia | India | LMIC | < 40 wks | 285 |
| Ali 2021 | 2020 | Prospective cohort | South Asia | Pakistan | LMIC | < 40 wks | 87 |
| Das 2021 | 2016 | Prospective cohort | South Asia | Bangladesh | LMIC | < 40 wks | 30 |
| Mokhtar 2021 | 2017-2018 | Prospective cohort | North Africa | Egypt | LMIC | < 40 wks | 240 |
| Naseer 2021 | NS | Prospective cohort | South Asia | Pakistan | LMIC | < 40 wks | 60 |
| Shaikh 2021 | 2019-2020 | Prospective cohort | South Asia | India | LMIC | < 40 wks | 156 |
| Singh 2021 | 2016 | Prospective cohort | South Asia | India | LMIC | < 40 wks | 171 |
| Trivedi 2021 | 2019 | Prospective cohort | South Asia | India | LMIC | < 40 wks | 224 |
| Chetan 2022 | 2019-2020 | Prospective cohort | South Asia | India | LMIC | < 40 wks | 25 |
| Imam 2022 | NS | Prospective cohort | North Africa | Egypt | LMIC | < 40 wks | 40 |
| Latha 2022 | 2018-2020 | Prospective cohort | South Asia | India | LMIC | > 28 wks/ > 1000 gm | 300 |
| Nguyen 2022 | 2019-2021 | Prospective cohort | Southeast Asia | Vietnam | LMIC | <36 wks/<2000 gm | 64 |
| Panda 2022 | 2017-2020 | Prospective cohort | South Asia | India | LMIC | < 40 wks | 48 |
| Parihar 2022 | 2018-2021 | Prospective cohort | South Asia | India | LMIC | <36 wks/< 2000 gm | 45 |
| Sisenda 2022 | 2020-2021 | Prospective cohort | East Africa | kenya | LMIC | <37 wks | 85 |
| Tuteja 2022 | 2018-2020 | Prospective cohort | South Asia | India | LMIC | < 40 wks | 157 |
| Ba-alwi 2022 | 2019-2020 | Retrospective | East Africa | Tanzania | LMIC | < 40 wks | 26 |
| Atul 2023 | 2019 | Prospective cohort | South Asia | India | LMIC | < 37 wks | 37 |
| Gupta 2023 | 2022 | Prospective cohort | South Asia | Nepal | LMIC | < 40 wks | 216 |
| Narsaiah 2023 | 2021-2023 | Prospective cohort | South Asia | India | LMIC | <34 wks /<2000 gm | 19 |
| Paudel 2023 | 2021 | Prospective cohort | South Asia | Nepal | LMIC | < 40 wks | 82 |
| Ravikant 2023 | 2022-2023 | Prospective cohort | South Asia | India | LMIC | <37 wks | 256 |
| Ravikant 2023 | 2022-2023 | Prospective cohort | South Asia | India | LMIC | <37 wks | 28 |
| Twanbasu 2023 | 2021-2022 | Prospective cohort | South Asia | Nepal | LMIC | < 40 wks | 111 |
| Ansari 2024 | 2022-2024 | Prospective cohort | South Asia | India | LMIC | < 40 wks | 136 |
| Awasthi 2024 | 2020 | Prospective cohort | South Asia | India | LMIC | < 40 wks | 70 |
| Basu 2014 | NS | Prospective cohort | South Asia | India | LMIC | < 40 wks | 566 |
| Chaya 2024 | 2012-2013 | Prospective cohort | South Asia | India | LMIC | < 34 wks | 3 |
| Kaur 2024 | NS | Prospective cohort | South Asia | India | LMIC | < 40 wks | 150 |
| Nayyar 2024 | 2021-2022 | Prospective cohort | South Asia | India | LMIC | <36 wks/<2000 gm | 15 |
| Niveditha 2024 | NS | Prospective cohort | South Asia | India | LMIC | < 40 wks | 51 |
| Sowmya 2024 | NS | Prospective cohort | South Asia | India | LMIC | < 40 wks | 60 |
| Srikanth 2024 | 2018-2020 | Prospective cohort | South Asia | India | LMIC | < 40 wks | 18 |
| Gandhi 2025 | 2024-2025 | Prospective cohort | South Asia | India | LMIC | < 40 wks | 31 |
| Madavi 2025 | 2022-2024 | Prospective cohort | South Asia | India | LMIC | < 40 wks | 85 |
| Malyas 2025 | 2021-2022 | Prospective cohort | East Africa | Tanzania | LMIC | <1500 gm | 99 |
| Srivastava 2025 | NS | Prospective cohort | South Asia | India | LMIC | <1500 gm | 94 |
| Vinod 2025 | 2020-2021 | Prospective cohort | South Asia | India | LMIC | < 40 wks | 150 |
| Singh 1993 | 1989-1991 | Retrospective | South Asia | India | LMIC | < 40 wks | 57 |
| Krishnan 1994 | 1991-1992 | Retrospective | South Asia | India | LMIC | < 40 wks | 58 |
| Maiya 1995 | 1991-1993 | Retrospective | South Asia | India | LMIC | < 40 wks | 121 |
| Bhutta 1999 | 1987-1993 | Retrospective | South Asia | Pakistan | LMIC | NA | 85 |
| Hariprasad 2000 | 1997-1999 | Retrospective | South Asia | India | LMIC | < 40 wks | 290 |
| Kadri 2006 | 2002 | Retrospective | Middle East Asia | Syria | LMIC | < 37 wks | 63 |
| Prabha 2014 | 2008-2013 | Retrospective | South Asia | India | LMIC | < 40 wks | 100 |
| Mannan 2016 | 2013-2014 | Retrospective | South Asia | Bangladesh | LMIC | < 40 wks | 50 |
| Patel 2016 | 2012-2015 | Retrospective | East Africa | Kenya | LMIC | <1500 gm | 32 |
| Srinivas 2016 | 2013-2015 | Retrospective | South Asia | India | LMIC | < 40 wks | 280 |
| Jahan 2017 | 2012-2013 | Retrospective | South Asia | Bangladesh | LMIC | < 40 wks | 58 |
| Hubbard 2018 | 2015-2016 | Retrospective | South Asia | Bangladesh | LMIC | < 40 wks | 58 |
| Gaber 2021 | 2018-2020 | Retrospective | North Africa | Egypt | LMIC | < 34 wks / < 2000 gm | NS |
| Regaieg 2022 | 2018-2019 | Retrospective | North Africa | Tunisia | LMIC | < 40 wks | 32 |
| Shah 2022 | 2017-2018 | Retrospective | South Asia | India | LMIC | < 40 wks | 23 |
| Gitau 2023 | 2009-2019 | Retrospective | East Africa | Kenya | LMIC | < 32 wks /< 1500 gm | NS |
| Patel 2023 | 2019-2020 | Retrospective | South Asia | India | LMIC | < 40 wks | 11 |
| Taj 2023 | 2022-2023 | Retrospective | South Asia | Pakistan | LMIC | < 40 wks | 153 |
| Benali 2024 | 2017-2021 | Retrospective | North Africa | Tunisia | LMIC | 26-31 wks | 156 |
| Bhat 2024 | 2019-2022 | Retrospective | South Asia | India | LMIC | >1500 gm | 16 |
| Habibul 2024 | 2024 | Retrospective | South Asia | India | LMIC | < 37 wks | 32 |
| Ranjan 2024 | NS | Retrospective | South Asia | India | LMIC | 28-34 wks | 70 |
| Shehadeh 2025 | 2020-2023 | Retrospective | Middle East Asia | Palestine | LMIC | <37 wks | 385 |
| Valentine 2025 | 2022-2023 | Retrospective | East Africa | Ethiopia | LIC | <37 wks | 64 |
| Regmi 2018 | 2015-2017 | Retrospective | South Asia | Nepal | LMIC | < 40 wks | 130 |
| Mathur 2005 | 2001 | Cross sectional | South Asia | India | LMIC | < 40 wks | 83 |
| Sharma 2017 | 2012-2013 | Cross sectional | South Asia | India | LMIC | < 40 wks | 72 |
| Salahuddin 2018 | 2015-2016 | Cross sectional | South Asia | Pakistan | LMIC | <37 wks | 45 |
| Nur 2021 | 2017-2018 | Cross sectional | East Africa | Ethiopia | LIC | < 40 wks | 163 |
| Roble 2022 | 2019 | Cross sectional | East Africa | Ethiopia | LIC | < 40 wks | 130 |
| Bhatta 2024 | 2018-2019 | Cross sectional | South Asia | Nepal | LMIC | < 40 wks | 164 |
| Sharafat 2024 | 2021-2023 | Cross sectional | South Asia | Pakistan | LMIC | <28 wks | 127 |
| Nasir 2025 | 2024-2024 | Cross sectional | South Asia | Pakistan | LMIC | < 40 wks | 89 |
| Jarousha 2008 | 2005 | Case control | Middle East | Palestine | LMIC | < 40 wks | 90 |
| Ariff 2011 | 1996-2006 | Case control | South Asia | Pakistan | LMIC | < 40 wks | 57 |
| Badr 2011 | 2010 | Case control | North Africa | Egypt | LMIC | < 40 wks | 56 |
| Merzougui 2018 | 2013-2015 | Case control | North Africa | Tunisia | LMIC | < 40 wks | 90 |
| Rameshwarnath 2018 | 2014-2015 | Case control | South Asia | India | LMIC | < 40 wks | 68 |
| Hussain 2020 | 2015-2018 | Case control | South Asia | Pakistan | LMIC | < 40 wks | 32 |
| Rani 2022 | 2016-2018 | Case control | South Asia | India | LMIC | < 40 wks | 106 |
| Kumar M 2023 | 2020 | Case control | South Asia | India | LMIC | < 40 wks | 90 |
| Mishra 2024 | 2021-2022 | Case control | South Asia | India | LMIC | <32 wks | 31 |
| Patel 2024 | NS | Case control | South Asia | India | LMIC | <32 wks/ <1500 gm | 111 |
| El-Hamid 2025 | 2022-2024 | Case control | North Africa | Egypt | LMIC | < 40 wks | 115 |

Abbreviations: BW- birth weight, GA- gestational age, n – sample size, wk- weeks, gm -grams, LIC-low-income countries, LMIC- low-middle income countries, NS-not specified.

**Table 2:** GRADE certainty of evidence for the secondary outcomes based on pooled adjusted odds ratio.

| **Certainty assessment** | | | | | | |  |
| --- | --- | --- | --- | --- | --- | --- | --- |
| **Participants (studies) Follow-up** | **Risk of bias** | **Inconsistency** | **Indirectness** | **Imprecision** | **Publication bias** | **Overall certainty of evidence** | **Relative effect (95% CI)** |
|  |  |  |  |  |  |  |  |
| Intraventricular Haemorrhage (IVH) | | | | | | | |
| 288 (3 non-randomised studies) | serious^a^ | serious^b^ | not serious | serious^c^ | none | ⨁◯◯◯ Very low^a,b,c^ | **OR 3.94** (0.14 to 108.9) |
|  |  |  |  |  |  |  |  |
| Sepsis | | | | | | | |
| 690 (3 non-randomised studies) | serious^a^ | not serious | not serious | not serious | none | ⨁⨁⨁◯ Moderate^a^ | **OR 4.93** (2.98 to 8.18) |
|  |  |  |  |  |  |  |  |

CI: confidence interval; OR: odds ratio

**Explanations**

a. Of the three studies, 1 study was assessed to be of high ROB, and 2 studies had some concerns.

b. Effect estimates varied widely across studies, with odds ratios ranging from 1.42 to 23.6 and substantial heterogeneity (I² = 86.6%).

c. The pooled estimate confidence interval was wide and crossed the line of no effect.

**Appendix 1 : Literature Search**

**Ovid MEDLINE(R) ALL**1946 to August 22, 2025

| **#** | **Searches** | **Results** |
| --- | --- | --- |
| 1 | exp respiration, artificial/ or exp high-frequency ventilation/ or exp high-frequency jet ventilation/ or exp interactive ventilatory support/ or exp liquid ventilation/ or exp noninvasive ventilation/ or exp one-lung ventilation/ or exp positive-pressure respiration/ or exp continuous positive airway pressure/ or exp intermittent positive-pressure breathing/ or exp intermittent positive-pressure ventilation/ or exp ventilator weaning/ or *Respiratory Insufficiency/mo [Mortality] | 94702 |
| 2 | (respirat* or ventilat* or resuscita* or (airway adj2 pressure) or (pressure adj2 breath*)).ti. | 290318 |
| 3 | (((respirat* or ventilat* or breath*) adj2 (mechanical or support*)) or persistent pulmonary hypertension).ti,ab,kf. | 88696 |
| 4 | 1 or 2 or 3 | 377390 |
| 5 | exp Infant, Newborn/ | 708021 |
| 6 | (newborn* or new* born* or neonate* or infant* or baby or babies or premie or preterm* or premature or "Low birth weight" or "Low birth weights" or "Low birthweight" or "Low birthweights" or Infant* or "Small gestational age" or SGA or "Extremely premature").ab,ti,kf. | 942424 |
| 7 | 5 or 6 | 1277896 |
| 8 | 4 and 7 | 48273 |
| 9 | (afghan* or africa* or albania* or algeria* or angola* or antigua* or barbuda* or argentin* or armenia* or aruba* or azerbaijan* or bahrain* or bangladesh* or bengal* or bangal* or barbados* or barbadian* or bajan or bajans or belarus* or belorus* or byelarus* or byelorus* or belize* or benin* or dahomey or bhutan* or bolivia* or bosnia* or herzegovin* or botswan* or batswan* or bechuanaland* or brazil* or brasil* or bulgaria* or burkina* or burkinese* or upper volta* or burundi* or urundi* or cabo verde* or cape verde* or cambodia* or kampuchea* or khmer* or cameroon* or cameroun* or ubangi shari* or chad* or chile* or china* or chinese or colombia* or comoro* or comore* or comorian* or mayotte* or congo* or zaire* or costa rica* or "cote d'ivoir*" or "cote d' ivoir*" or cote divoir* or cote d ivoir* or ivory coast* or ivorian* or croatia* or cuba or cuban or cubans or "cuba's" or cyprus* or cypriot* or czech* or djibouti* or french somaliland* or dominica* or ecuador* or egypt* or united arab republic* or el salvador* or salvadoran* or guinea* or equatoguinea* or eritrea* or estonia* or eswatini* or swaziland* or swazi* or swati* or ethiopia* or fiji* or gabon* or gabonese* or gabonaise* or gambia* or ((georgia or georgian or georgians) not (atlanta or california or florida)) or ghana* or gibraltar* or greece* or greek* or grecian* or grenada* or grenadian* or guam* or guatemala* or guyana* or guiana* or guyanese* or haiti* or hispaniola* or hondura* or hungary* or hungarian* or india* or indonesia* or iran* or iraq* or jamaica* or jordan* or kazakh* or kenya* or karabati* or korea* or kosovo* or kosova* or kyrgyz* or kirgiz* or kirghiz* or laos or lao or laotian* or latvia* or lebanon* or lebanese* or lesotho* or lesothan* or lesothonian* or basutoland* or mosotho* or basotho* or liberia* or libya* or jamahiriya* or lithuania* or macedonia* or madagasca* or malagasy* or malawi* or nyasaland* or malaysia* or malay* federation or maldives* or maldivian* or indian ocean or mali or malian* or "mali's" or malta or maltese* or "malta's" or micronesia* or marshallese* or kiribati* or marshall island* or nauru or nauran or nauruans or "naurian's" or mariana or marianas or palau or paluan* or tuvalu* or mauritania* or mauritan* or mauritius* or mexico* or mexican* or moldova* or moldovia* or mongol* or montenegr* or morocco* or moroccan* or ifni or mozambique* or mozambican* or myanmar* or burma* or burmese or namibia* or nepal* or new caledonia* or netherlands antill* or nicaragua* or niger* or oman or omani or omanis or "oman's" or pakistan* or palestin* or gaza* or west bank* or panama* or paraguay* or peru or peruvian* or "peru's" or philippine* or philipine* or phillipine* or phillippine* or filipino* or filipina* or poland* or polish or pole or poles or portugal* or portuguese or puerto ric* or romania* or russia* or ussr* or soviet* or rwanda* or rwandese or ruanda* or ruandese or samoa* or navigator island* or pacific island* or polynesia* or "sao tome and principe*" or sao tomean* or santomean* or saudi arabia* or saudi or saudis or senegal* or serbia* or seychell* or sierra leone* or slovak* or sloven* or melanesia* or solomon island* or norfolk island* or somali* or sri lanka* or ceylon* or "saint kitts and nevis*" or "st kitts and nevis*" or kittian* or nevisian* or saint lucia* or st lucia* or saint vincent* or st vincent* or vincentian* or grenadine* or sudan* or surinam* or syria* or tajik* or tadjik* or tadzhik* or tanzania* or tanganyika* or thai* or timor leste* or east timor* or timorese* or togo or togoles* or "togo's" or tonga* or trinidad* or tobago* or tunisia* or turkiy* or turkey* or turk or turks or turkish or turkmen* or uganda* or ukrain* or uruguay* or uzbek* or vanuatu* or new hebrides* or venezuela* or vietnam* or viet nam* or yemen* or yugoslav* or zambia* or zimbabwe* or rhodesia* or arab* countr* or middle east* or global south or sahara* or subsahara* or magreb* or maghrib* or west indies* or caribbean* or central america* or latin america* or south america* or central asia* or north asia* or northern asia* or southeastern asia* or south eastern asia* or southeast asia* or south east asia* or west asia* or western asia* or east europe* or eastern europe* or developing countr* or developing nation* or developing population* or developing world or less developed countr* or less developed nation* or less developed world or lesser developed countr* or lesser developed nation* or lesser developed world or under developed countr* or under developed nation* or under developed world or underdeveloped countr* or underdeveloped nation* or underdeveloped world or middle income countr* or middle income nation* or middle income population* or low income countr* or low income nation* or low income population* or lower income countr* or lower income nation* or lower income population* or underserved countr* or underserved nation* or underserved population* or under served population* or under served nation* or under served population* or deprived countr* or deprived population* or high burden countr* or high burden nation* or countdown countr* or countdown nation* or poor countr* or poor nation* or poor population* or poor world or poorer countr* or poorer nation* or poorer population* or poorer world or developing econom* or less developed econom* or underdeveloped econom* or under developed econom* or middle income econom* or low income econom* or lower income econom* or low gdp or low gnp or low gross domestic or low gross national or lower gdp or lower gnp or lower gross domestic or lower gross national or lmic or lmics or third world or lami countr* or transitional countr* or emerging econom* or emerging nation*).ti,ab,hw,kf. | 3651873 |
| 10 | 8 and 9 | 5372 |
| 11 | *morbidity/ or *mortality/ or *fatal outcome/ or *infant mortality/ or *survival rate/ | 48044 |
| 12 | (Mortalit* or morbidit* or died or death or die).ab,kf,ti. | 2382693 |
| 13 | (pulmonary adj2 (complication* or hypertension* or hypotension* or air leak*)).ab,kf,ti. | 78800 |
| 14 | (Bronchopulmonary dysplasia or (chronic adj2 lung adj2 disease) or Pneumothorax or lung injur* or atelectasis or Intraventricular hemorrhag* or Intraventricular haemorrhag* or Periventricular leukomalacia or ((Neurodevelopmental or neurological) adj2 disabilit*) or ((visual or vision or hearing) adj2 loss) or Cardiovascular complication* or Patent ductus arteriosus or Systemic hypotension or Readmission* or Pulmonary hemorrhage* or Pulmonary haemorrhage* or Shock* or hypotension or hypoperfusion*).ab,kf,ti. | 612875 |
| 15 | ((hypertension adj2 secondary) or sepsis or Ventilator-associated pneumonia).ab,kf,ti. | 151926 |
| 16 | ((Feeding or gastrointestinal) adj3 (complication* or intolerance)).ab,kf,ti. | 8327 |
| 17 | (Necrotizing enterocolitis or retinopathy of prematurity or Growth failur*).ab,kf,ti. | 22108 |
| 18 | ((kidney adj2 injur*) or EUGR).ab,kf,ti. | 60116 |
| 19 | or/11-18 | 3051149 |
| 20 | 10 and 19 | 2816 |

**Embase Classic+Embase**1947 to 2025 October 09

| **#** | **Searches** | **Results** |
| --- | --- | --- |
| 1 | exp artificial ventilation/ or exp respiratory failure/ or exp ventilator weaning/ | 469130 |
| 2 | (respirat* or ventilat* or resuscita* or (airway adj2 pressure) or (pressure adj2 breath*)).ti. | 400166 |
| 3 | (((respirat* or ventilat* or breath*) adj2 (mechanical or support*)) or persistent pulmonary hypertension).ti,ab,kf. | 154777 |
| 4 | 1 or 2 or 3 | 771897 |
| 5 | exp newborn/ | 764848 |
| 6 | (newborn* or new* born* or neonate* or infant* or baby or babies or premie or preterm* or premature or "Low birth weight" or "Low birth weights" or "Low birthweight" or "Low birthweights" or Infant* or "Small gestational age" or SGA or "Extremely premature").ab,ti,kf. | 1302102 |
| 7 | 5 or 6 | 1656829 |
| 8 | 4 and 7 | 87930 |
| 9 | (afghan* or africa* or albania* or algeria* or angola* or antigua* or barbuda* or argentin* or armenia* or aruba* or azerbaijan* or bahrain* or bangladesh* or bengal* or bangal* or barbados* or barbadian* or bajan or bajans or belarus* or belorus* or byelarus* or byelorus* or belize* or benin* or dahomey or bhutan* or bolivia* or bosnia* or herzegovin* or botswan* or batswan* or bechuanaland* or brazil* or brasil* or bulgaria* or burkina* or burkinese* or upper volta* or burundi* or urundi* or cabo verde* or cape verde* or cambodia* or kampuchea* or khmer* or cameroon* or cameroun* or ubangi shari* or chad* or chile* or china* or chinese or colombia* or comoro* or comore* or comorian* or mayotte* or congo* or zaire* or costa rica* or "cote d'ivoir*" or "cote d' ivoir*" or cote divoir* or cote d ivoir* or ivory coast* or ivorian* or croatia* or cuba or cuban or cubans or "cuba's" or cyprus* or cypriot* or czech* or djibouti* or french somaliland* or dominica* or ecuador* or egypt* or united arab republic* or el salvador* or salvadoran* or guinea* or equatoguinea* or eritrea* or estonia* or eswatini* or swaziland* or swazi* or swati* or ethiopia* or fiji* or gabon* or gabonese* or gabonaise* or gambia* or ((georgia or georgian or georgians) not (atlanta or california or florida)) or ghana* or gibraltar* or greece* or greek* or grecian* or grenada* or grenadian* or guam* or guatemala* or guyana* or guiana* or guyanese* or haiti* or hispaniola* or hondura* or hungary* or hungarian* or india* or indonesia* or iran* or iraq* or jamaica* or jordan* or kazakh* or kenya* or karabati* or korea* or kosovo* or kosova* or kyrgyz* or kirgiz* or kirghiz* or laos or lao or laotian* or latvia* or lebanon* or lebanese* or lesotho* or lesothan* or lesothonian* or basutoland* or mosotho* or basotho* or liberia* or libya* or jamahiriya* or lithuania* or macedonia* or madagasca* or malagasy* or malawi* or nyasaland* or malaysia* or malay* federation or maldives* or maldivian* or indian ocean or mali or malian* or "mali's" or malta or maltese* or "malta's" or micronesia* or marshallese* or kiribati* or marshall island* or nauru or nauran or nauruans or "naurian's" or mariana or marianas or palau or paluan* or tuvalu* or mauritania* or mauritan* or mauritius* or mexico* or mexican* or moldova* or moldovia* or mongol* or montenegr* or morocco* or moroccan* or ifni or mozambique* or mozambican* or myanmar* or burma* or burmese or namibia* or nepal* or new caledonia* or netherlands antill* or nicaragua* or niger* or oman or omani or omanis or "oman's" or pakistan* or palestin* or gaza* or west bank* or panama* or paraguay* or peru or peruvian* or "peru's" or philippine* or philipine* or phillipine* or phillippine* or filipino* or filipina* or poland* or polish or pole or poles or portugal* or portuguese or puerto ric* or romania* or russia* or ussr* or soviet* or rwanda* or rwandese or ruanda* or ruandese or samoa* or navigator island* or pacific island* or polynesia* or "sao tome and principe*" or sao tomean* or santomean* or saudi arabia* or saudi or saudis or senegal* or serbia* or seychell* or sierra leone* or slovak* or sloven* or melanesia* or solomon island* or norfolk island* or somali* or sri lanka* or ceylon* or "saint kitts and nevis*" or "st kitts and nevis*" or kittian* or nevisian* or saint lucia* or st lucia* or saint vincent* or st vincent* or vincentian* or grenadine* or sudan* or surinam* or syria* or tajik* or tadjik* or tadzhik* or tanzania* or tanganyika* or thai* or timor leste* or east timor* or timorese* or togo or togoles* or "togo's" or tonga* or trinidad* or tobago* or tunisia* or turkiy* or turkey* or turk or turks or turkish or turkmen* or uganda* or ukrain* or uruguay* or uzbek* or vanuatu* or new hebrides* or venezuela* or vietnam* or viet nam* or yemen* or yugoslav* or zambia* or zimbabwe* or rhodesia* or arab* countr* or middle east* or global south or sahara* or subsahara* or magreb* or maghrib* or west indies* or caribbean* or central america* or latin america* or south america* or central asia* or north asia* or northern asia* or southeastern asia* or south eastern asia* or southeast asia* or south east asia* or west asia* or western asia* or east europe* or eastern europe* or developing countr* or developing nation* or developing population* or developing world or less developed countr* or less developed nation* or less developed world or lesser developed countr* or lesser developed nation* or lesser developed world or under developed countr* or under developed nation* or under developed world or underdeveloped countr* or underdeveloped nation* or underdeveloped world or middle income countr* or middle income nation* or middle income population* or low income countr* or low income nation* or low income population* or lower income countr* or lower income nation* or lower income population* or underserved countr* or underserved nation* or underserved population* or under served population* or under served nation* or under served population* or deprived countr* or deprived population* or high burden countr* or high burden nation* or countdown countr* or countdown nation* or poor countr* or poor nation* or poor population* or poor world or poorer countr* or poorer nation* or poorer population* or poorer world or developing econom* or less developed econom* or underdeveloped econom* or under developed econom* or middle income econom* or low income econom* or lower income econom* or low gdp or low gnp or low gross domestic or low gross national or lower gdp or lower gnp or lower gross domestic or lower gross national or lmic or lmics or third world or lami countr* or transitional countr* or emerging econom* or emerging nation*).ti,ab,hw,kf. | 4796350 |
| 10 | 8 and 9 | 9506 |
| 11 | *morbidity/ or *mortality/ or *fatal outcome/ or *infant mortality/ or *survival rate/ | 199609 |
| 12 | (Mortalit* or morbidit* or died or death or die).ab,kf,ti. | 3688508 |
| 13 | (pulmonary adj2 (complication* or hypertension* or hypotension* or air leak*)).ab,kf,ti. | 137595 |
| 14 | (Bronchopulmonary dysplasia or (chronic adj2 lung adj2 disease) or Pneumothorax or lung injur* or atelectasis or Intraventricular hemorrhag* or Intraventricular haemorrhag* or Periventricular leukomalacia or ((Neurodevelopmental or neurological) adj2 disabilit*) or ((visual or vision or hearing) adj2 loss) or Cardiovascular complication* or Patent ductus arteriosus or Systemic hypotension or Readmission* or Pulmonary hemorrhage* or Pulmonary haemorrhage* or Shock* or hypotension or hypoperfusion*).ab,kf,ti. | 939525 |
| 15 | ((hypertension adj2 secondary) or sepsis or Ventilator-associated pneumonia).ab,kf,ti. | 252379 |
| 16 | ((Feeding or gastrointestinal) adj3 (complication* or intolerance)).ab,kf,ti. | 13241 |
| 17 | (Necrotizing enterocolitis or retinopathy of prematurity or Growth failur*).ab,kf,ti. | 32121 |
| 18 | ((kidney adj2 injur*) or EUGR).ab,kf,ti. | 104253 |
| 19 | or/11-18 | 4694540 |
| 20 | 10 and 19 | 5481 |
| 21 | limit 20 to "remove medline records" | 2618 |

CENTRAL (Cochrane) Date Run:12/10/2025 22:57:31

| **ID** | **Search** | **Hits** |
| --- | --- | --- |
| #1 | MeSH descriptor: [Infant, Newborn] explode all trees | 23948 |
| #2 | MeSH descriptor: [Infant, Premature] explode all trees | 5806 |
| #3 | MeSH descriptor: [Infant, Low Birth Weight] explode all trees | 2951 |
| #4 | (Newborn* or infant* or preemies or Preterm* or premature or "Low birth weight" or lbw or vlbw or elbw or "Low birth weights" or "Low birthweight" or "Low birthweights" or "pre-terms" or "Pre-term" or "Extremely premature"):ti,ab,kw (Word variations have been searched) | 104294 |
| #5 | {OR #1-#4} | 104294 |
| #6 | MeSH descriptor: [Respiration, Artificial] explode all trees | 9398 |
| #7 | MeSH descriptor: [Respiratory Insufficiency] this term only | 2479 |
| #8 | (respirat* or ventilat* or resuscita* or (airway NEAR/2 pressure) or (pressure NEAR/2 breath*)):ti (Word variations have been searched) | 34720 |
| #9 | (((respirat* or ventilat* or breath*) NEAR/2 (mechanical or support*)) or persistent pulmonary hypertension):ti,ab | 19439 |
| #10 | {OR #6-#9} | 48726 |
| #11 | #5 AND #10 | 8478 |
| #12 | Afghanistan or Benin or "Burkina Faso" or Burundi or "Central African Republic" or Guinea or Chad or Comoros or "Congo Dem Rep" or Congo or Eritrea or Ethiopia or Gambia or "Guinea Bissau" or Haiti or Korea or "Dem Peoples Rep" or Somalia or Liberia or Madagascar or Malawi or Mali or Mozambique or Nepal or Niger or Rwanda or Senegal or "Sierra Leone" or "South Sudan" or Tanzania or Togo or Uganda or Zimbabwe | 41489 |
| #13 | Angola or Armenia or Bangladesh or Bhutan or Bolivia or "Cabo Verde" or Cambodia or Cameroon or "Cote d’Ivoire" or Djibouti or Egypt or "El Salvador" or Georgia or Ghana or Guatemala or Honduras or India or Indonesia or Jordan or Kenya or Kiribati or Kosovo or "Kyrgyz Republic" or "Lao PDR" or Lao or Lesotho or Mauritania or Micronesia or "Fed Sts" or Moldova or Mongolia or Morocco or Myanmar or Nicaragua or Nigeria or Pakistan or "Papua New Guinea" or Philippines or "Sao Tome" or Principe or "Solomon Islands" or "Sri Lanka" or Sudan or Swaziland or "Syrian Arab Republic" or syria or Tajikistan or "Timor Leste" or Tunisia or Ukraine or Uzbekistan or Vanuatu or Vietnam or "West Bank Gaza" or Yemen or Zambia | 93348 |
| #14 | Albania or Algeria or "American Samoa" or Argentina or Azerbaijan or Belarus or Belize or Bosnia or Herzegovina or Botswana or Brazil or Bulgaria or China or Colombia or "Costa Rica ORCroatia" or Cuba or Dominica or "Dominica Republic" or Ecuador or "Equatorial Guinea" or Fiji or Gabon or Grenada or Guyana or Iran or "Islamic Rep" or Iraq or Jamaica or Kazakhstan or Lebanon or Libya or Macedonia or FYR Malaysia or Maldives or "Marshall Islands" or Mauritius or Mexico or Montenegro or Namibia or Nauru or Panama or Paraguay or Peru or Romania or "Russian Federation" or Samoa or Serbia or "South Africa" or "St. Lucia" or "St. Vincent" or Grenadines or Suriname or Thailand or Tonga or Turkey or Turkmenistan or Tuvalu or Venezuela | 188547 |
| #15 | (Africa or Asia or Caribbean or "West Indies" or "South America" or "Latin America" or "Central America") | 26791 |
| #16 | ((developing or "less* developed" or "under developed" or underdeveloped or "middle income" or "low* income" or underserved or "under served" or deprived or poor*) NEAR (countr* or nation* or population* or world)) | 15310 |
| #17 | ((developing or "less* developed" or "under developed" or underdeveloped or "middle income" or "low* income") NEXT (economy or economies)) | 61 |
| #18 | low NEXT (GDP or GNP or "gross domestic" or "gross national") | 48 |
| #19 | (LMIC or LMICs or "third world" or "LAMI country" or "LAMI countries") | 1328 |
| #20 | ("transitional country" or "transitional countries") | 28 |
| #21 | {OR #12-#20} | 322526 |
| #22 | #11 AND #21 | 1611 |
| #23 | MeSH descriptor: [Morbidity] this term only | 1125 |
| #24 | MeSH descriptor: [Mortality] this term only | 992 |
| #25 | MeSH descriptor: [Fatal Outcome] this term only | 21 |
| #26 | MeSH descriptor: [Infant Mortality] this term only | 826 |
| #27 | MeSH descriptor: [Survival Rate] this term only | 13464 |
| #28 | (Mortalit* or morbidit* or died or death or die):ab,kw,ti | 216385 |
| #29 | (pulmonary NEAR/2 (complication* or hypertension* or hypotension* or air leak*)):ab,kw,ti | 8479 |
| #30 | (Bronchopulmonary dysplasia or (chronic NEAR/2 lung NEAR/2 disease) or Pneumothorax or lung injur* or atelectasis or Intraventricular hemorrhag* or Intraventricular haemorrhag* or Periventricular leukomalacia or ((Neurodevelopmental or neurological) NEAR/2 disabilit*) or ((visual or vision or hearing) NEAR/2 loss) or Cardiovascular complication* or Patent ductus arteriosus or Systemic hypotension or Readmission* or Pulmonary hemorrhage* or Pulmonary haemorrhage* or Shock* or hypotension or hypoperfusion*):ab,kw,ti | 97191 |
| #31 | ((hypertension NEAR/2 secondary) or sepsis or Ventilator-associated pneumonia):ab,kw,ti | 17584 |
| #32 | ((Feeding or gastrointestinal) NEAR/3 (complication* or intolerance)):ab,kw,ti | 2733 |
| #33 | (Necrotizing enterocolitis or retinopathy of prematurity or Growth failur*):ab,kw,ti | 7381 |
| #34 | ((kidney NEAR/2 injur*) or EUGR):ab,kw,ti | 7109 |
| #35 | {OR #23-#34} | 305546 |
| #36 | #22 AND #35 | 1080 |
| #37 | Removed Embase and PubMed citations (CT.GOV 104, ICTRP 32 and CINAHL 3) | 139 |

**Appendix 2:**  Subgroup analysis for primary outcome in-hospital mortality

Prespecified subgroup analyses were undertaken across country, geographical region, gestational age strata, and underlying respiratory aetiology.

**Country-specific:**

Mortality varied across countries. Studies from India (38 studies; 4,591 neonates) showed a pooled in-hospital mortality of 44% (95% CI: 37–51%; I² = 92.7%). Comparable estimates were observed in Pakistan (7 studies; 510 neonates), with a pooled mortality of 46% (95% CI: 28–64%; I² = 85.5%), Nepal (6 studies; 743 neonates) 49% (95% CI: 27–72%; I² = 95.9%) and Bangladesh (7 studies; 509 neonates) 49% (95% CI: 34–63%; I² = 81.2%). In contrast, lower pooled mortality was noted in Egypt (6 studies; 688 neonates) 28% (95% CI: 16–43%; I² =90.4%). Substantial heterogeneity was observed across all country-level analyses. The test for subgroup differences were statistically significant (Q = 66.79, df = 8, p < 0.0001). (**Figure S9 in Supplementary file 2**).

**Geographical region**

When subgroup analysis was performed by region, most evidence were from South Asia (58 studies; 6,353 neonates), with a pooled mortality of 45% (95% CI: 40–51%; I² =92%). Mortality was higher in East Africa (3 studies; 122 neonates) 66% (95% CI: 2–100%; I² = 94%) and the Middle East (1 study; 24) 80% (95% CI: 61–92%). In contrast, the mortality rate was lower in North Africa (6 studies; 688 neonates) of 28% (95% CI: 16–43%; I² =90.4%). Differences across regions were statistically significant (Q = 20.60, df = 3, p = 0.0001) (**Figure S10 in Supplementary file 2**).

**Gestational age strata**

Among studies with a inclusion of neonates with gestational age of <34 weeks (4 studies; 236 neonates), pooled mortality was 39% (95% CI: 7–84%; I² = 92.8%). In neonates with gestation age of less than 37 weeks (5 studies; 218 neonates) reported higher mortality of 55%, (95% CI: 30–78%; I² = 83.2%). The largest subgroup, were neonates of all gestation. i.e., below 40 weeks (59 studies; 6,739 neonates), showed a pooled mortality of 44% (95% CI: 39–50%; I² = 92.6% ). **(Figure S11 in Supplementary file 2).**

**Underlying respiratory aetiology**

Mortality also differed by respiratory aetiology. Neonates ventilated for respiratory distress syndrome (6 studies; 301 neonates) had a pooled mortality of 38% (95% CI: 18–62%;I^2^= 85.4%). Limited data were available for pulmonary arterial hypertension (2 studies; 65 neonates), with a mortality of 10% (95% CI: 0–100%; I^2^=72.9%). A single study of neonates needing ventilation for meconium aspiration syndrome (44 neonates) reported a high mortality of 80% (95% CI: 65–90%) **(Figure S12 in Supplementary file 2).**
